# Hidden species diversity in *Sylvirana nigrovittata* (Amphibia: Ranidae) highlights the importance of taxonomic revisions in biodiversity conservation

**DOI:** 10.1371/journal.pone.0192766

**Published:** 2018-03-14

**Authors:** Jennifer A. Sheridan, Bryan L. Stuart

**Affiliations:** 1 Division of Science, Yale-NUS College, Singapore, Singapore; 2 Section of Research & Collections, North Carolina Museum of Natural Sciences, Raleigh, NC United States of America; Universita degli Studi della Tuscia, ITALY

## Abstract

Accurately delimiting species and their geographic ranges is imperative for conservation, especially in areas experiencing rapid habitat loss. Southeast Asia currently has one of the highest rates of deforestation in the world, is home to multiple biodiversity hotspots, and the majority of its countries have developing economies with limited resources for biodiversity conservation. Thus, accurately delimiting species and their ranges is particularly important in this region. We examined genetic and morphological variation in the widespread frog species *Sylvirana nigrovittata* (and its long-treated junior synonym *S*. *mortenseni*) with the goal of clarifying its taxonomic content and geographic range boundaries for conservation. We present evidence that the current concept of *S*. *nigrovittata* contains at least eight species, two of which are each known from only two localities, but that *S*. *mortenseni* is more geographically widespread than currently realized. Five of these species are described as new to science.

## Introduction

Accurately delimiting species is essential for effective conservation of biodiversity [1]. It is not possible to assess the biodiversity conservation status of a given area without knowing which species it contains and how geographically widespread those species are. Despite increased attention on habitats and ecosystems [2–4], individual species remain the primary units of conservation. Funding agencies and government policy continue to focus on species rather than habitats, and therefore accurate understanding of species distributions is imperative for informing conservation policy. The world’s biodiversity hotspots are concentrated in the tropics [5], where large numbers of undescribed species, including undetected cryptic species, remain unrecognized [6–8].

Cryptic species are two or more distinct species that are erroneously classified (= hidden) under a single species name [6]. Cryptic species are taxonomically widespread, found throughout the animal kingdom [9–11], and represented by both terrestrial and aquatic organisms [12, 13]. Such cryptic species pose challenges to taxonomists and conservation managers alike. From a taxonomist’s perspective, it can be difficult to diagnose species that exhibit minimal morphological divergence, and non-visual traits such as genetics or vocalizations (usually unavailable from historical specimens) often become the primary means of distinguishing among members of these complexes [8, 14]. From a conservation manager’s perspective, a lack of clarity on diversity and distributions often requires that cryptic species be lumped as their single nominate species during threat assessments, thus down-grading the acute conservation needs of individual cryptic species [8, 15].

Amphibians have increasingly been shown to harbor cryptic species diversity [7, 8]. Given that amphibians are considered to be the world’s most threatened vertebrate group [16], it is essential to assess the extent of cryptic diversity in this group in order to prioritize conservation actions. Southeast Asia is a biodiversity hotspot [5] that simultaneously is experiencing the highest deforestation rate of all major tropical rainforest areas [17, 18]. This means that large numbers of amphibian species are threatened by habitat loss, and that species with restricted geographic ranges are especially at risk. Even amphibian species that are commonly found in human-modified habitats (e.g., the four-lined tree-frog [*Polypedates leucomystax*], the Asian common toad [*Duttaphrynus melanostictus*], the grass frog [*Fejervarya limnocharis*]) are now known or suspected to represent complexes of cryptic species [19–21].

*Sylvirana nigrovittata* (Blyth 1856) is a notable example of a Southeast Asian frog species that has long been suspected to be hiding cryptic species diversity. Members of this species (or its “species complex”) have a pale brown to dark brown dorsum; flank generally darker than dorsum; dorsolateral folds, with a black band (sometimes broken) of variable width extending from the tip of the snout to the groin along the lower margin of the dorsolateral fold; cream venter, sometimes with dark spotting; humeral glands in adult males; visible tympanum; and the upper one-third of the iris lighter in coloration than the lower two-thirds. Originally described from Myanmar, this species is currently recognized as having a wide geographic range in mainland Southeast Asia that extends from northeastern India to Peninsular Malaysia [22, 23]. Smith [24] provided evidence that *S*. *nigrovittata* is distinguishable from, but closely related to, *S*. *mortenseni* [25], a species described from Thailand and Myanmar. The following year, Smith [26] reversed his assessment and synonymized the two species, treating *S*. *mortenseni* as only a geographical variant of *S*. *nigrovittata*. In addition, Smith [26] recognized two additional, unnamed geographical variants of *S*. *nigrovittata*, one from peninsular Thailand and one from northeastern Thailand and Laos. Analyses of allozymic variation in samples of *S*. *nigrovittata* from across Thailand recovered two major genetic groups, one of which contained topotypic samples of *S*. *mortenseni* and therein justified its synonymy with *S*. *nigrovittata* [27]. Ohler et al. [28] removed *S*. *mortenseni* from the synonymy of *S*. *nigrovittata* on the basis of morphology, extended the range of *S*. *mortensensi* into mainland Cambodia and northeastern Thailand, and cited unpublished morphological data that suggested the presence of as many as five distinct species within the *S*. *nigrovittata* complex in Thailand and Indochina. Oliver et al. [29] found that six samples from Vietnam and Myanmar that morphologically resembled *S*. *nigrovittata* failed to form a monophyletic group within the genus in molecular phylogenetic analyses of mitochondrial and nuclear genes. Nearly a century of observation on morphological variation, and recent molecular analyses, together provide compelling evidence that *S*. *nigrovittata* harbors cryptic species. However, to date no comprehensive analysis of variation across the range of *S*. *nigrovittata* (herein including *S*. *mortenseni*) has been conducted, but such an analysis is required to resolve this long-standing taxonomic uncertainty [30]. The generic status of *S*. *nigrovittata* has been in flux [29], but we follow Frost et al. [31] and Oliver et al. [29] in recognizing *Sylvirana* Dubois, 1992 at the genus rank.

Herein, we present such an analysis of morphological and genetic variation (representing loci from both the mitochondrial and nuclear genomes) in samples of *S*. *nigrovittata* obtained from across mainland Southeast Asia that represent most of its currently recognized geographic range. We seek corroborated evidence from these multiple, independent datasets that *S*. *nigrovittata* consists of more than one distinct evolutionary lineage (= species). We further use these findings to clarify the taxonomy and geographic ranges of these species in an effort to facilitate a revised evaluation of the conservation needs of each contained species.

## Materials and methods

### Sampling

Specimens were collected in the field by the authors during 1998–2013, temporarily housed in individual plastic bags, humanely euthanized using immersion in tricaine methanesulfonate (MS-222; [32]), and fixed in 10% buffered formalin after preserving liver in 20% DMSO-salt saturated storage buffer, ≥95% ethanol, or RNAlater (Invitrogen). Specimens were later transferred to 70% ethanol for permanent storage. Specimens and tissue samples were deposited at the Field Museum of Natural History (FMNH), the North Carolina Museum of Natural Sciences (NCSM), and the Department of Biology, Faculty of Natural Science, National University of Laos (NUOL). Specimens and tissues were also borrowed from the holdings of these and other institutions (S1 & S2 Tables).

### DNA extraction, amplification, and sequencing

Whole genomic DNA was extracted from 349 liver or muscle samples (S1 Table) using the 5Prime ArchivePure DNA Cell/Tissue Kit (5Prime). A 467 bp fragment of mitochondrial DNA (mtDNA) that encodes part of the cytochrome oxidase c subunit III (COXIII) gene, the complete tRNA glycine (Gly), the complete NADH dehydrogenase subunit 3 (ND3) gene, and part of the tRNA arginine (Arg), a 596–606 bp fragment of mtDNA that encodes part of the 16S ribosomal RNA (16S) gene, and a 1,058–1,084 bp fragment of mtDNA that encodes part of the tRNA methionine (Met), the complete NADH deydrogenase subunit 2 (ND2) gene, and part of the tRNA tryptophan (Trp) gene were amplified by PCR (the polymerase chain reaction) following Stuart et al. [8]. A 607–613 bp fragment of nuclear DNA (nuDNA) that encodes part of the proopiomelanocortin (POMC) gene was amplified by PCR (94°C 45s, 57°C 30s, 72°C 1 min) for 35 cycles using the primer pair POMC-1 and POMC-2 [33]. A 996–1,002 bp fragment of nuDNA that encodes part of the sodium/calcium exchanger 1 (NCX1) gene was amplified by PCR (94°C 45s, 53°C 30s, 72°C 1 min) for 35 cycles using the primer pair NCX_1F and NCX_3R [34]. PCR products were cleaned using GELase (Epicentre Technologies) or ExoSAP-IT (Affymetrix), and sequenced in both directions by direct double strand cycle sequencing using Big Dye version 3 chemistry (Perkin Elmer) and the amplifying primers. Sequences were edited using Sequencher v. 4.1 (Genecodes) and deposited in GenBank under accession numbers MG605710–MG607352 (S1 Table).

### Phylogenetic analysis

Homologous sequences of the outgroup taxa *Babina holsti*, *Pelophylax nigromaculatus*, *Hydrophylax leptoglossa*, *Hylarana erythraea*, *Indosylvirana attigua*, *Sylvirana cubitalis*, and *S*. *maosonensis* [following 29, 35, 36] were downloaded from GenBank (S1 Table). These and the newly generated sequences were aligned using default settings in MAFFT [37], followed by visual verification. Alignments were partitioned by gene and by codon position (if relevant), and concatenated mtDNA and concatenated nuDNA datasets were analyzed separately.

Mixed-model Bayesian phylogenetic inference was performed using MrBayes 3.2.6 [38] on the Cyberinfrastructure for Phylogenetic Research (CIPRES) Science Gate Way 3.3 [39]. The best partitioning scheme, and best-fitting model of sequence evolution for each partition that is available for implementation in MrBayes 3.2.6, were selected using the Bayesian Information Criterion (BIC) in PartitionFinder 1.1.0 [40]. The six selected partitions and models in the mtDNA dataset were GTR+I+Γ for COXIII third codon position, Met, and ND2 first codon position; GTR+Γ for COXIII first codon position, ND2 second codon position, and ND3 second codon position; JC for COXIII second codon position; GTR+I+Γ for 16S, Gly, ND3 first codon position, and Trp; GTR+I+Γ for ND2 third codon position and ND3 third codon position; and K80 for Arg. The three selected partitions and models in the nuDNA dataset were SYM+I+Γ for NCX and POMC third codon positions; HKY+I+Γ for NCX and POMC first codon positions; and F81 for NCX and POMC second codon positions. Four independent Bayesian runs were performed on each dataset. For the mtDNA dataset, four chains ran for 10,000,000 generations using the default priors, the chain temperature was set to 0.1, trees were sampled every 2,000 generations, and the first 25% of trees were discarded as burn in. For the nuDNA dataset, four chains ran for 30,000,000 generations using the default priors, the chain temperature was set to 0.1, trees were sampled every 3,000 generations, and the first 50% of trees were discarded as burn in. Trace plots were viewed using Tracer 1.6 [41].

An unrooted phylogenetic network of the concatenated nuDNA dataset was constructed using SplitsTree v4.14.4 [42] with uncorrected ‘p’ genetic distances and the NeighborNet algorithm to visualize overall divergence patterns while accounting for reticulate (non-branching) relationships. Only samples having both POMC and NCX sequence data were included in the phylogenetic network analysis.

### Morphological analysis

Only sexually mature individuals were used in the morphological analyses, as determined by the presence of secondary sexual characteristics (vocal sac openings, nuptial pads, and/or humeral glands in males, and convoluted oviducts or mature ova in females). Morphometric data were taken only by the first author to minimize inter-observer bias [43, 44]. Twelve measurements were taken using Mitutoyo digital calipers, to the nearest 0.1 mm under a Meiji EMZ dissecting microscope: snout-vent length (SVL), head length from tip of snout to rear of jaws (HDL), maximum head width (HDW), snout length from tip of snout to anterior corner of eye (SNT), eye diameter (EYE), interorbital distance (IOD), internasal distance (IND), horizontal diameter of tympanum (TMP), shank length (SHK), thigh length (TGH), manus length from tip of third digit to base of outer palmar tubercle (HND), and pes length from tip of fourth toe to base of inner metatarsal tubercle (FTL). Manus length (HND) and FTL were measured on the left side of the body, and all remaining measurements were taken from the right side. Toe disc and phalanx width were also measured in holotypes and these measurements are reported to the nearest 0.01 mm. Reported errors around means are standard deviations (SD; Table 1). Raw morphological data are given in S1 Appendix.

**Table 1:** Measurements of adult *Sylvirana nigrovittata* complex in mm (range, mean ± SD). This table includes all individuals utilized for discriminant function analysis (DFA), which contains both sequenced and unsequenced individuals. Note that data are presented for females of *S*. *annamitica* sp. nov., *S*. *lacrima* sp. nov., and *S*. *roberti* sp. nov., even though there were too few individuals of each to include in DFA.

	*S*. *nigrovittata*		*S*. *mortenseni*		*S*. *montosa* sp. nov.		*S*. *annamitica* sp. nov.		*S*. *malayana* sp. nov.		*S*. *roberti* sp. nov.		*S*. *lacrima* sp. nov.	
	Males (n = 92)	Females (n = 51)	Males (n = 54)	Females (n = 17)	Males (n = 56)	Females (n = 35)	Males (n = 16)	Females (n = 5)	Males (n = 22)	Females (n = 7)	Males (n = 12)	Females (n = 1)	Males (n = 12)	Females (n = 6)
SVL	34.3–54.0 44.6 ± 4.2	42.3–55.6 49.3 ± 3.3	46.0–80.9 62.7 ± 8.7	47.7–69.2 60.3 ± 6.2	44.7–74.7 57.8 ± 6.2	49.1–69.0 59.1 ± 5.2	40.2–52.0 45.0 ± 2.9	49.5–53.7 51.2 ± 1.6	42.2–48.8 44.7 ± 2.0	47.2–56.8 52.7 ± 3.2	41.6–45.4 43.6 ± 1.3	49.2	38.5–45.5 42.1 ± 2.0	46.7–66.8 55.7 ± 8.1
HDL	12.9–21.8 17.6 ± 1.9	15.9–21.7 18.5 ± 1.3	17.9–31.3 25.0 ± 3.7	18.4–25.9 22.7 ± 2.2	17.9–28.5 23.0 ± 2.4	18.6–24.7 22.0 ± 1.8	15.2–19.0 17.6 ± 1.0	17.9–20.3 18.8 ± 1.2	14.8–18.2 16.6 ± 0.9	17.9–21.9 19.8 ± 1.3	16.0–18.0 17.0 ± 0.7	17.7	13.9–16.4 14.9 ± 0.8	15.8–23.4 19.6 ± 2.9
HDW	12.4–19.5 16.0 ± 1.6	13.6–19.2 16.7 ± 1.2	16.1–31.8 23.5 ± 3.8	17.3–25.3 20.8 ± 2.1	17.0–27.9 21.6 ± 2.3	17.0–24.0 20.4 ± 1.8	13.6–17.9 15.9 ± 1.3	16.3–18.4 17.3 ± 0.8	13.3–16.0 14.6 ± 0.8	14.9–18.0 16.6 ± 1.0	14.4–16.0 14.9 ± 0.4	15.9	12.9–14.5 13.7 ± 0.7	15.0–21.8 18.3 ± 2.8
SNT	5.1–8.3 6.6 ± 0.7	5.6–8.1 7.0 ± 0.6	6.2–12.9 9.5 ± 1.4	6.8–10.7 8.6 ± 1.0	6.6–11.2 8.5 ± 0.9	7.0–9.3 8.3 ± 0.7	5.8–7.5 6.6 ± 0.4	6.9–7.6 7.2 ± 0.3	6.0–7.5 6.7 ± 0.5	6.9–8.7 8.1 ± 0.6	6.3–7.2 6.8 ± 0.3	7.0	5.5–7.1 6.1 ± 0.4	6.5–9.7 8.0 ± 1.3
EYE	4.6–7.0 5.8 ± 0.6	5.1–7.2 6.2 ± 0.5	6.0–9.3 7.7 ± 0.9	5.4–8.9 7.3 ± 0.9	5.7–8.9 7.3 ± 0.7	5.8–8.4 7.2 ± 0.7	5.3–6.8 6.1 ± 0.4	6.3–7.1 6.5 ± 0.5	4.9–6.3 5.7 ± 0.3	6.0–7.0 6.5 ± 0.4	5.6–6.3 5.9 ± 0.2	6.1	5.0–6.4 5.5 ± 0.4	5.3–7.7 6.7 ± 0.8
IOD	3.3–5.6 4.0 ± 0.4	3.3–4.8 4.1 ± 0.4	3.2–7.9 5.4 ± 0.9	3.7–5.7 4.9 ± 0.6	3.7–6.6 5.1 ± 0.7	3.9–5.8 4.8 ± 0.5	3.2–5.4 4.3 ± 0.6	4.2–4.8 4.6 ± 0.3	3.4–4.9 4.0 ± 0.4	3.6–5.0 4.5 ± 0.5	3.6–4.3 3.9 ± 0.2	3.7	3.2–4.2 3.6 ± 0.3	3.9–5.4 4.6 ± 0.6
IND	3.7–5.7 4.6 ± 0.4	4.0–5.5 4.8 ± 0.3	4.3–8.0 6.1 ± 0.8	4.7–6.5 5.6 ± 0.5	4.5–6.9 5.7 ± 0.6	4.6–6.5 5.6 ± 0.6	4.1–5.4 4.8 ± 0.3	4.4–5.6 5.2 ± 0.5	4.2–5.2 4.5 ± 0.3	4.2–5.5 5.0 ± 0.4	4.2–5.0 4.5 ± 0.2	4.8	3.6–4.5 3.9 ± 0.3	4.4–6.3 5.1 ± 0.8
TMP	3.3–5.7 4.3 ± 0.5	3.6–5.0 4.2 ± 0.4	4.2–7.2 5.8 ± 0.7	4.8–6.4 5.3 ± 0.3	3.7–6.8 5.4 ± 0.6	4.0–6.5 5.2 ± 0.6	3.9–5.3 4.6 ± 0.4	4.0–4.8 4.4 ± 0.3	3.7–5.2 4.2 ± 0.3	4.3–5.3 4.5 ± 0.4	3.9–4.4 4.2 ± 0.1	4.6	3.0–4.3 3.5 ± 0.4	3.5–4.5 4.0 ± 0.4
SHK	18.6–26.4 23.5 ± 2.5	21.4–31.0 25.6 ± 2.3	21.7–44.8 33.2 ± 5.3	24.7–37.9 32.1 ± 4.1	23.6–37.8 30.7 ± 3.7	25.1–35.1 30.8 ± 3.0	21.5–26.4 23.6 ± 1.3	26.0–27.6 27.2 ± 0.9	21.2–27.2 24.2 ± 1.6	25.8–31.0 29.6 ± 1.9	21.4–25.2 23.8 ± 1.2	25.7	22.8–25.3 24.3 ± 0.8	27.5–37.7 32.9 ± 4.1
TGH	16.5–29.2 22.8 ± 2.7	19.4–28.8 24.5 ± 2.3	18.5–43.5 31.9 ± 6.1	21.4–36.7 29.6 ± 3.9	20.4–34.1 28.2 ± 3.1	24.0–34.1 28.6 ± 2.8	17.9–24.9 21.0 ± 2.4	22.8–25.5 23.8 ± 1.1	19.5–25.6 22.1 ± 1.8	21.7–28.3 26.4 ± 2.3	21.3–23.8 22.6 ± 0.7	24.6	21.3–24.9 22.6 ± 1.1	26.2–34.7 30.4 ± 3.5
HND	9.5–14.8 11.9 ± 1.3	10.8–16.1 12.8 ± 1.1	10.8–21.7 15.6 ± 2.4	12.2–17.4 14.5 ± 1.6	11.7–18.6 14.8 ± 1.6	12.2–17.6 14.7 ± 1.4	10.0–12.7 11.5 ± 0.9	12.2–13.7 13.0 ± 0.6	10.2–13.5 11.6 ± 0.8	11.7–15.1 13.5 ± 1.1	10.7–12.2 11.5 ± 0.5	12.2	9.0–10.7 9.8 ± 0.6	11.3–15.6 13.3 ± 1.7
FTL	18.5–29.6 23.6 ± 2.4	20.9–29.3 25.2 ± 2.0	21.7–45.0 31.8 ± 5.2	23.6–34.6 29.3 ± 3.6	22.8–36.9 29.7 ± 3.2	23.5–34.9 29.8 ± 3.0	20.1–26.3 23.2 ± 1.8	24.0–27.3 26.1 ± 1.2	20.5–25.3 22.5 ± 1.5	24.9–28.8 27.3 ± 1.4	21.8–24.7 22.8 ± 0.7	24.1	20.3–24.6 22.0 ± 1.3	23.5–33.2 28.5 ± 4.0

Measurements used in discriminant function analysis (DFA) were corrected for differences in ontogenetic composition [45] using the following allometric equation: X_adj_ = X– β(SVL–SVL_mean_), where X_adj_ is the adjusted value of the morphometric variable and X is the original value; SVL_mean_ is the overall mean SVL for a given clade, β is the within-clade coefficient of the linear regression of X against SVL [46, 47]. DFA was performed on X_adj_ and SVL values using JMP Pro 9 [48] to determine whether the nominate species overlap in morphospace. Samples were assigned to species in the DFA based on their assignment in the mtDNA Bayesian analysis (below). DFA was performed only for putative species that had measurement and sequence data for at least three individuals per sex. Thus, DFA was performed on males of all species but only females of *S*. *nigrovittata*, *S*. *mortenseni*, *S*. *annamitica* sp. nov., *S*. *montosa* sp. nov., and *S*. *malayana* sp. nov.

### Nomenclatural acts

The electronic edition of this article conforms to the requirements of the amended International Code of Zoological Nomenclature, and hence the new names contained herein are available under that Code from the electronic edition of this article. This published work and the nomenclatural acts it contains have been registered in ZooBank, the online registration system for the ICZN. The ZooBank LSIDs (Life Science Identifiers) can be resolved and the associated information viewed through any standard web browser by appending the LSID to the prefix “http://zoobank.org/”. The LSID for this publication is: urn:lsid:zoobank.org:pub:4CDDD642-D6FE-47E8-AC15-3F5AD0148F2B. The electronic edition of this work was published in a journal with an ISSN, and has been archived and is available from the following digital repositories: PubMed Central, LOCKSS.

## Results

### Phylogenetic analysis

The aligned mtDNA dataset contained 2,183 characters of 351 taxa (344 individuals of the *S*. *nigrovittata* complex), and the aligned nuDNA dataset contained 1,617 characters of 342 taxa (335 individuals of the *S*. *nigrovittata* complex). The standard deviation of split frequencies among the four Bayesian runs was 0.007004 in the mtDNA analysis and 0.031448 in the nuDNA analysis. The effective sample sizes (ESS) obtained in Tracer [41] was ≥ 509 in the mtDNA analysis and ≥ 374 in the nuDNA analysis.

**Fig 1:**
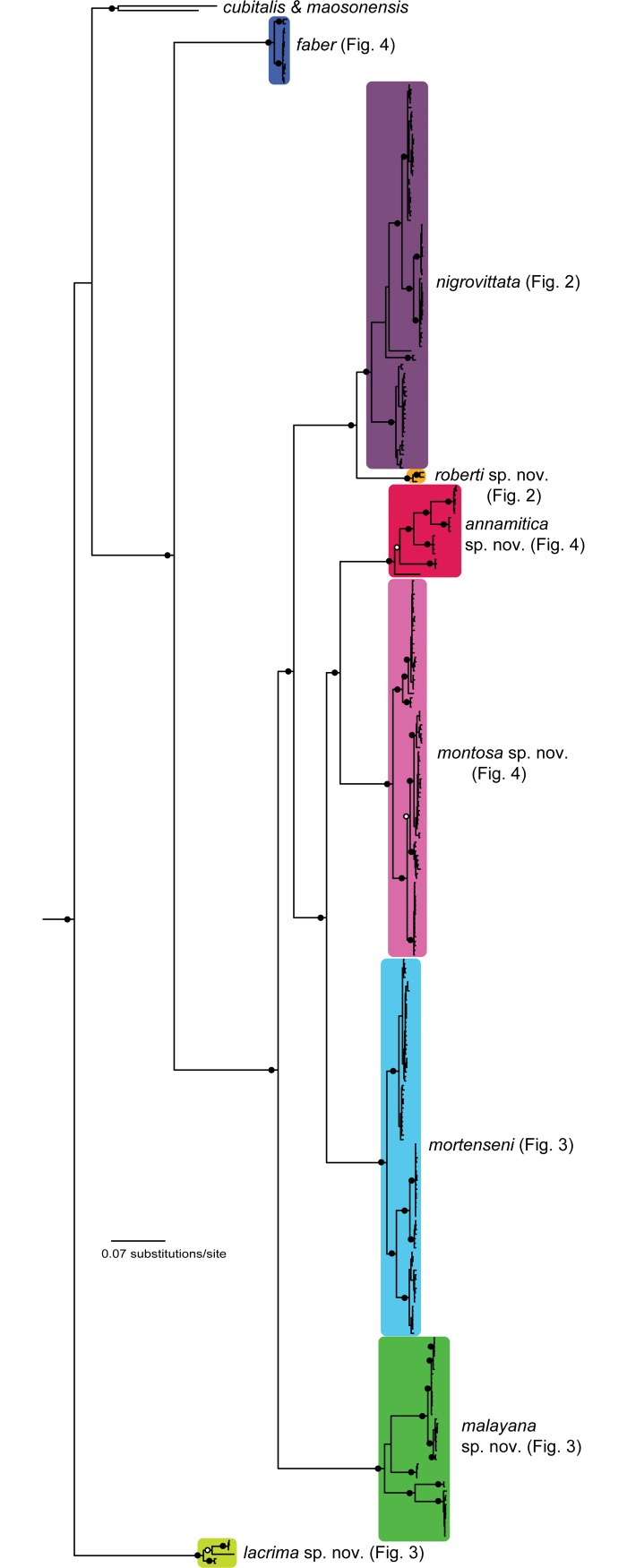
Fifty percent majority-rule consensus phylogram resulting from mixed-model Bayesian analysis of the concatenated mtDNA dataset (2,183 aligned characters) from 344 individuals of the *Sylvirana nigrovittata* species complex. Outgroups also include *Babina holsti*, *Pelophylax nigromaculatus*, *Hydrophylax leptoglossa*, *Hylarana erythraea* and *Indosylvirana attigua* (not shown). Black circles at nodes indicate Bayesian posterior probabilities ≥ 0.99, and open circles at nodes indicate Bayesian posterior probabilities ≥ 0.95.

**Fig 2:**
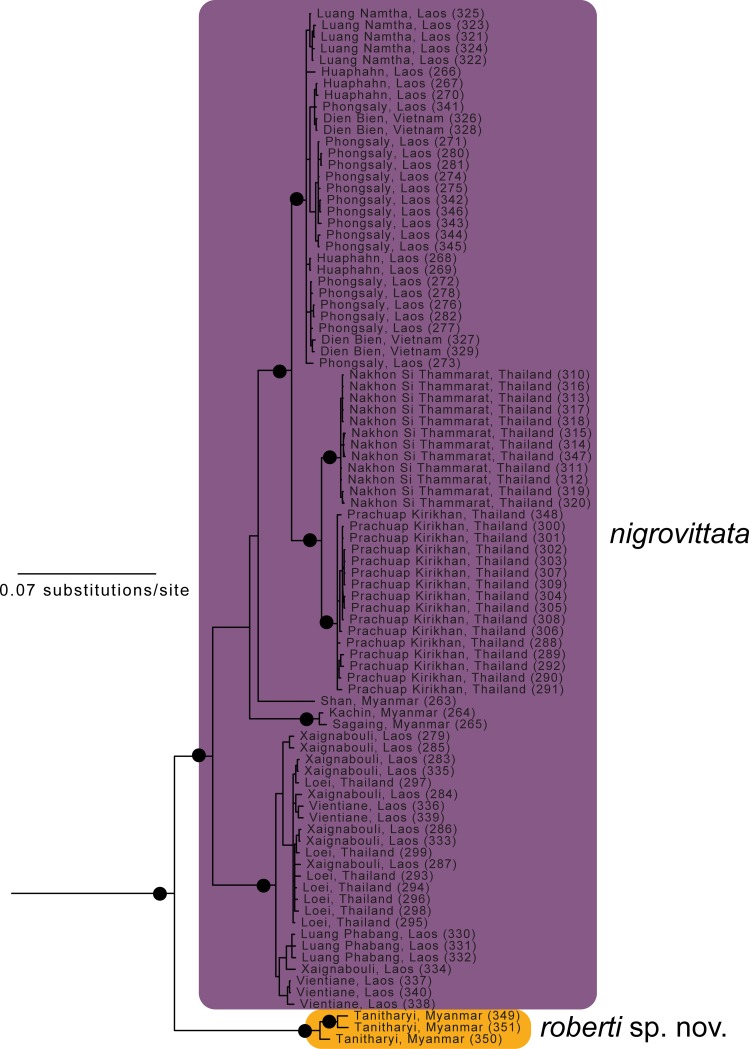
Enlargement of part of Fig 1 showing relationships within *Sylvirana nigrovittata* and *S*. *roberti* sp. nov. resulting from mixed-model Bayesian analysis of the concatenated mtDNA dataset. Black circles at nodes indicate Bayesian posterior probabilities ≥ 0.99, and open circles at nodes indicate Bayesian posterior probabilities ≥ 0.95. Numbers in parentheses at terminal tips refer to sample identification numbers in S1 Table.

**Fig 3:**
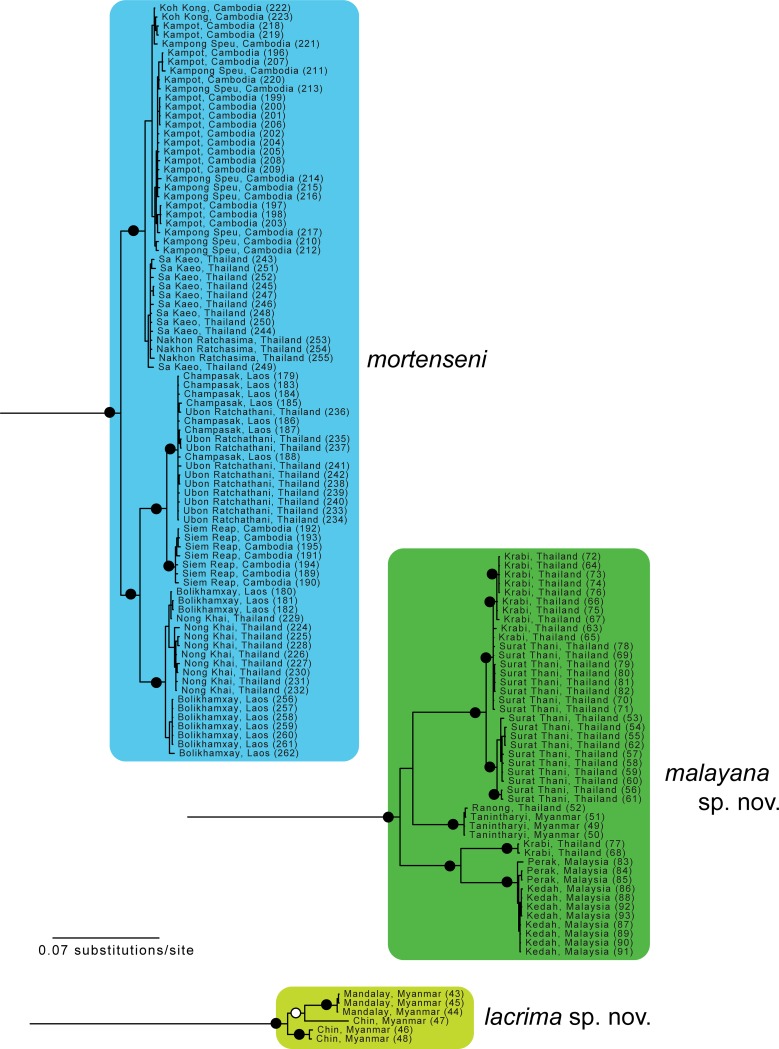
Enlargement of part of Fig 1 showing relationships within *Sylvirana mortenseni*, *S*. *malayana* sp. nov. and *S*. *lacrima* sp. nov. resulting from mixed-model Bayesian analysis of the concatenated mtDNA dataset. Black circles at nodes indicate Bayesian posterior probabilities ≥ 0.99, and open circles at nodes indicate Bayesian posterior probabilities ≥ 0.95. Numbers in parentheses at terminal tips refer to sample identification numbers in S1 Table.

**Fig 4:**
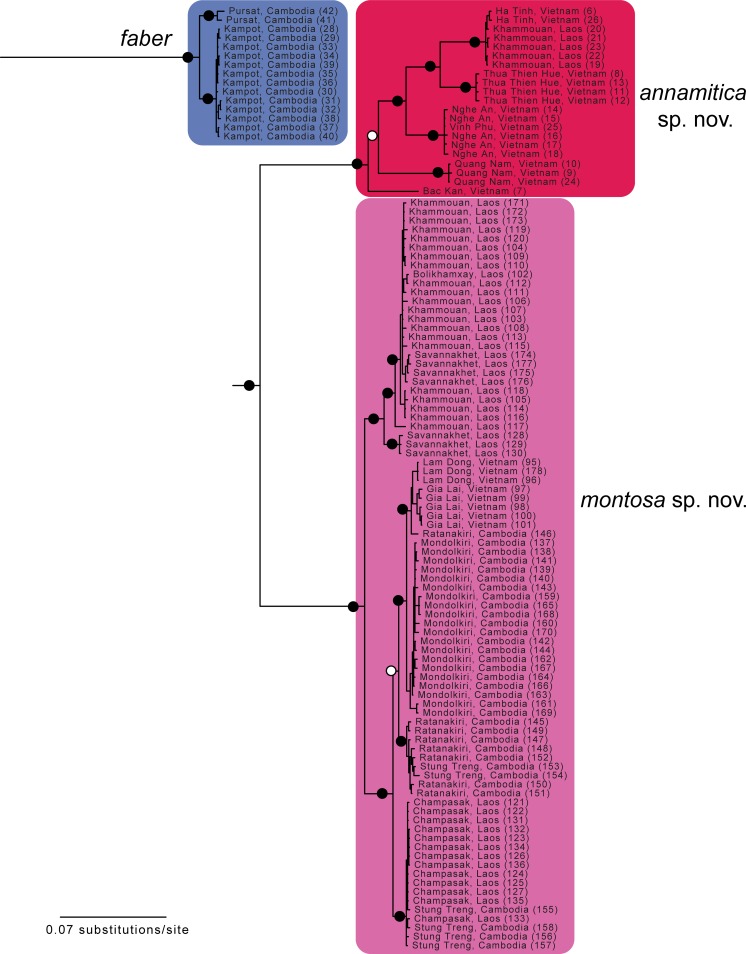
Enlargement of part of Fig 1 showing relationships within *Sylvirana faber*, *S*. *annamitica* sp. nov., and *S*. *montosa* sp. nov. resulting from mixed-model Bayesian analysis of the concatenated mtDNA dataset. Black circles at nodes indicate Bayesian posterior probabilities ≥ 0.99, and open circles at nodes indicate Bayesian posterior probabilities ≥ 0.95. Numbers in parentheses at terminal tips refer to sample identification numbers in S1 Table.

The mtDNA Bayesian analysis found strong support for the monophyly of *Sylvirana* (Fig 1). Samples of the *S*. *nigrovittata* complex were recovered in at least eight major mtDNA clades, but samples referred to this complex from Chin and Mandalay States, Myanmar, rendered the complex non-monophyletic with respect to *S*. *cubitalis* and *S*. *maosonensis* (Figs 1–4).

**Fig 5:**
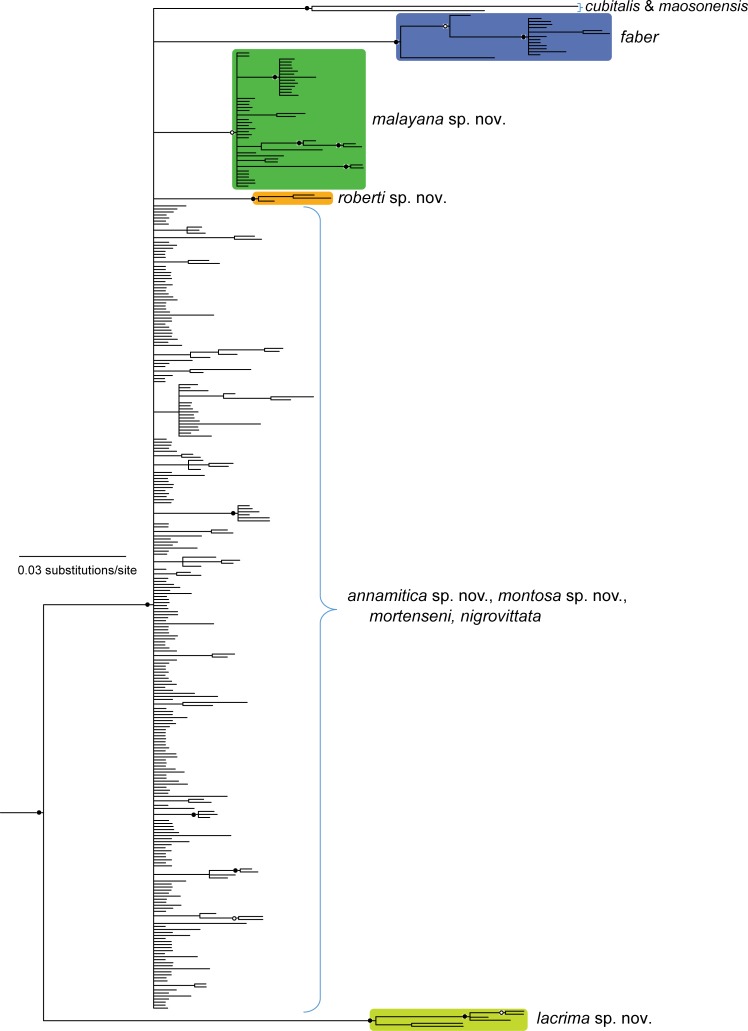
Fifty percent majority-rule consensus phylogram resulting from mixed-model Bayesian analysis of the concatenated nuDNA dataset (1,617 aligned characters) from 335 individuals of the *Sylvirana nigrovittata* species complex. Outgroups also include *Babina holsti*, *Pelophylax nigromaculatus*, *Hydrophylax leptoglossa*, *Hylarana erythraea* and *Indosylvirana attigua* (not shown). Black circles at nodes indicate Bayesian posterior probabilities ≥ 0.99, and open circles at nodes indicate Bayesian posterior probabilities ≥ 0.95.

**Fig 6:**
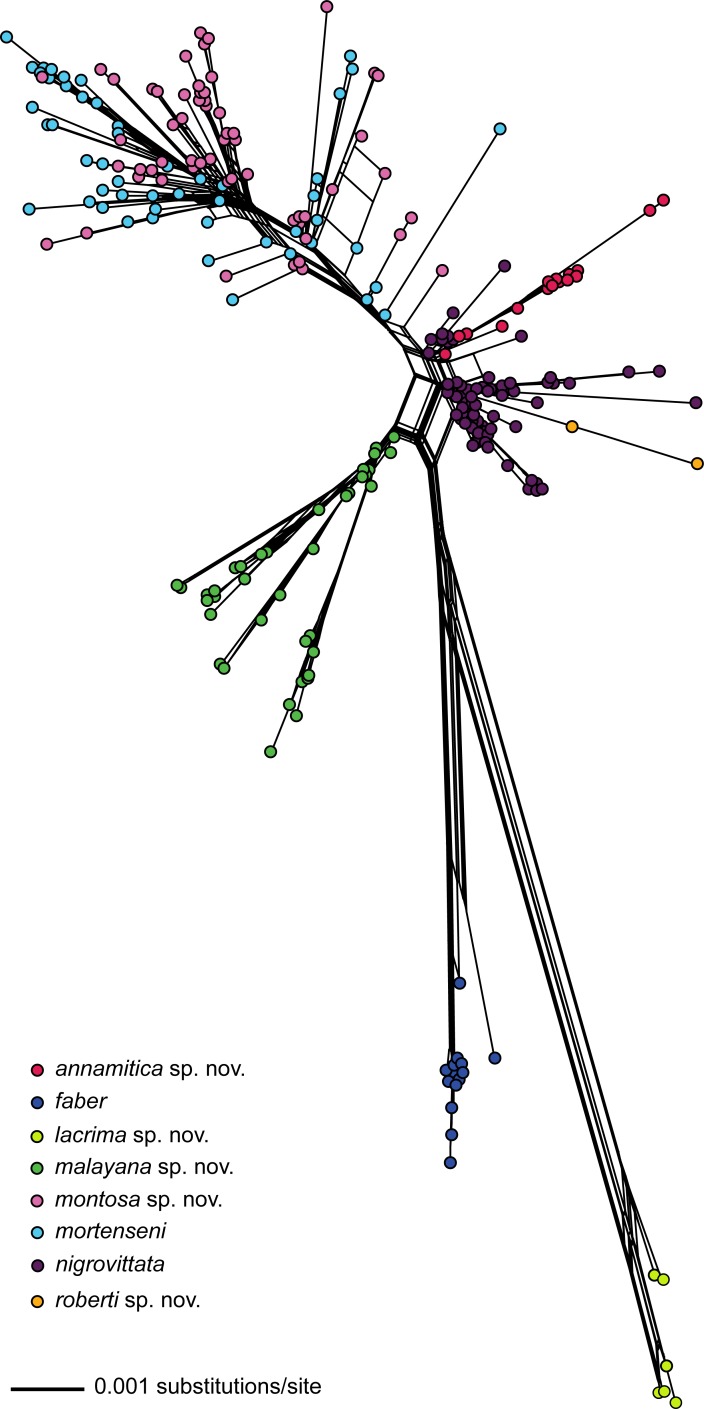
Phylogenetic network of the concatenated nuDNA dataset (1,617 aligned characters) from 257 individuals of the *Sylvirana nigrovittata* species complex using uncorrected ‘p’ genetic distances and the NeighborNet algorithm. Only samples with sequence data for both nuclear genes were included in the analysis.

The nuDNA Bayesian analysis also found strong support for the monophyly of *Sylvirana* and the non-monophyly of the *S*. *nigrovittata* complex with respect to to *S*. *cubitalis* and *S*. *maosonensis* owing to the same samples from Chin and Mandalay States, Myanmar (Fig 5). However, the nuDNA Bayesian analysis revealed much less limited genetic structure among samples of the *S*. *nigrovittata* complex, with only four of the eight major mtDNA clades also recovered in the nuDNA Bayesian analysis (those referred to below as *S*. *faber*, *S*. *malayana* sp. nov., *S*. *roberti* sp. nov., and *S*. *lacrima* sp. nov.; Fig 5). The unrooted phylogenetic network of the nuDNA data (Fig 6) revealed more genetic structure than did the nuDNA Bayesian analysis, with considerable admixture between two mtDNA lineages (those referred to as *S*. *mortensensi* and *S*. *montosa* sp. nov., below), minimal admixture of two additional mtDNA lineages (those referred to as *S*. *nigrovittata* and *S*. *annamitica* sp. nov., below), and no admixture of the four lineages that were also reciprocally monophyletic in the Bayesian mtDNA and nuDNA analyses (those referred to below as *S*. *faber*, *S*. *malayana* sp. nov., *S*. *roberti* sp. nov., and *S*. *lacrima* sp. nov.).

### Morphological analysis

**Fig 7:**
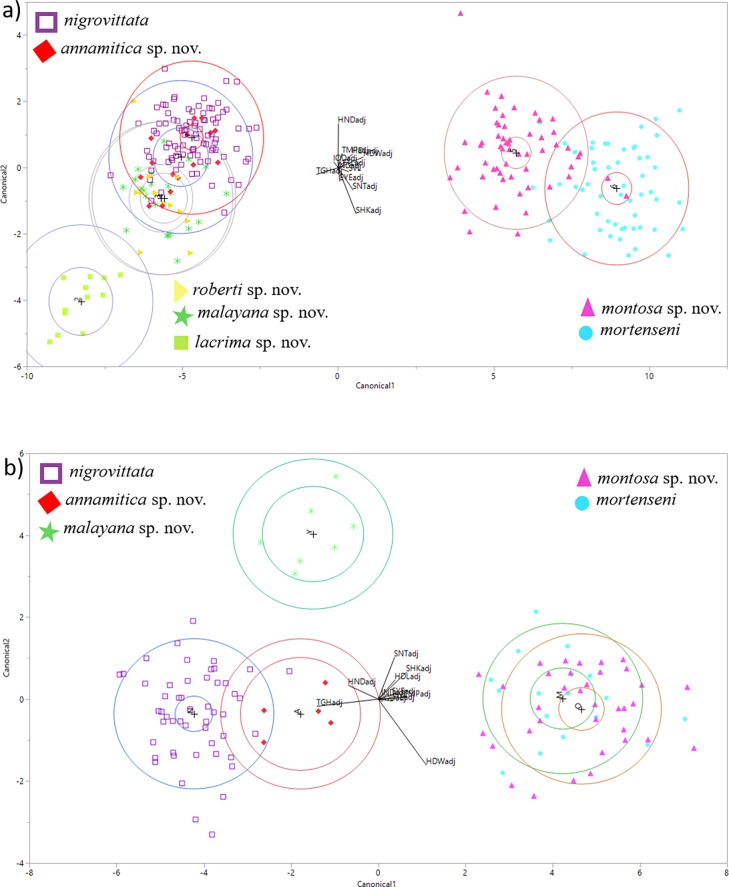
**Discriminant function analysis (DFA) of morphological features of a) male *Sylvirana nigrovittata* (purple open squares), *S*. *mortenseni* (blue circles), *S*. *annamitica* sp. nov. (red diamonds), *S*. *malayana* sp. nov. (green stars), *S*. *roberti* sp. nov. (yellow triangles), *and S*. *lacrima* sp. nov. (green closed squares); and b) female *Sylvirana nigrovittata*, *S*. *mortenseni*, *S*. *annamitica* sp. nov., and *S*. *malayana* sp. nov. (symbols for each species same as for males).** Individual symbols represent individuals of a given species.

Overall, DFA of males correctly assigned 85% of specimens to their species (based on mtDNA clades), with correct classifications of species ranging from 69–100% (Table 2; Fig 7A). Males of *S*. *lacrima* sp. nov. (below) were very distinct in morphospace (100% correct classification rates), but males of remaining species overlapped in morphospace with at least one other species. While *S*. *mortenseni* slightly overlapped with *S*. *montosa* sp. nov. (below), *S*. *malayana* sp. nov. (below) strongly overlapped with *S*. *roberti* sp. nov. (below), and *S*. *nigrovittata* strongly overlapped with *S*. *annamitica* sp. nov. (below) (Fig 7A).

**Table 2:** Results of discriminant function analysis (DFA) of male morphological features used to classify species in the *Sylvirana nigrovittata* species complex.

		Predicted Group							Percent correct
Actual	*annamitica* sp. nov.	*lacrima* sp. nov.	*malayana* sp. nov.	*montosa* sp. nov.	*mortenseni*	*nigrovittata*	*roberti* sp. nov.	N	
*annamitica* sp. nov.	11	0	0	0	0	4	1	16	69
*lacrima* sp. nov.	0	12	0	0	0	0	0	12	100
*malayana* sp. nov.	0	0	17	0	0	0	5	22	77
*montosa* sp. nov.	0	0	0	51	5	0	0	56	91
*mortenseni*	0	0	0	3	51	0	0	54	94
*nigrovittata*	11	0	2	0	0	74	5	92	80
*roberti* sp. nov.	0	0	1	0	0	1	10	12	83

Overall, DFA of females (only five species analyzed) correctly assigned 95% of specimens to their mtDNA clade (Fig 7B). *Sylvirana annamitica* sp. nov. and *S*. *malayana* sp. nov. (below) were correctly classified 100% of the time, *S*. *mortenseni* and *S*. *nigrovittata* were correctly classified 94% of the time, and *S*. *montosa* sp. nov. (below) was correctly classified 86% of the time.

### Species accounts

We follow Inger et al. [49] in adopting a conservative operational criterion of recognizing as species only those lineages that are diagnosable in more than one independent dataset, in this case, in morphology, mtDNA and/or nuDNA. Following this operational criterion, herein we recognize eight distinct species in the *S*. *nigrovittata* complex. These include *S*. *nigrovittata*, *S*. *mortenseni*, *S*. *faber*, and five previously unnamed species, as follows.

***Sylvirana nigrovittata* (Blyth, 1856)**

*Limnodytes nigrovittatus* Blyth, 1856: 718 [50].

*Rana nigrovittata* Sclater, 1892: 345 [51].

*Rana* (*Hylorana*) *nigrovittata* Boulenger, 1920: 124 [52].

*Rana* (*Hylarana*) *nigrovittata* Bourret, 1942: 318 [53].

*Hylarana nigrovittata* Chen, Murphy, Lathrop, Ngo, Orlov, Ho, and Somorjai, 2005: 237 [54]; Che, Pang, Zhao, Wu, Zhao and Zhang, 2007: 3 [55].

*Rana (Sylvirana) nigrovittata* Dubois, 1992: 341 [56].

*Sylvirana nigrovittata* Frost, Grant, Faivovich, Bain, Haas, Haddad, de Sá, Channing, Wilkinson, Donnellan, Raxworthy, Campbell, Blotto, Moler, Drewes, Nussbaum, Lynch, Green, and Wheeler, 2006: 370 [31]; Oliver, Prendini, Kraus, and Raxworthy, 2015: 191 [29].

*Hylarana (Sylvirana) nigrovittata* Fei, Ye, Jiang, and Xie, 2008: 199 [57].

*Hylarana (Sylvirana) menglaensis* Fei, Ye and Xie, 2008: 201 [57]; Le, Pham, Nguyen, Ziegler and Nguyen, 2014: 315 [58].

*Hylarana (Sylvirana) hekouensis* Fei, Ye and Jiang, 2008: 199 [57].

#### Lectotype

BMNH 1947.2.2.99 (= BMNH 1893.2.14.4), female, either immature or post-reproductive in having small, immature ova, Mergui, Myanmar, coll. by W. Theobald. Dubois [56] designated the lectotype as “BMNH 1947.2.2.93 = 1893.2.14.4.” However, BMNH 1947.2.2.99 is the catalog number that was re-assigned to the original number of BMNH 1893.2.14.4, the specimen BMNH 1947.2.2.99 is listed as “one of the types” of *Rana nigrovittata*, and no specimen with the number BMNH 1947.2.2.93 could be located in the catalog or type cabinet (David J. Gower, personal communication, 29 March 2013). We therefore assume that the re-assigned catalog number reported by Dubois [56] contained a typographical error, and that BMNH 1947.2.2.99 is the correct catalog number for the lectotype.

#### Expanded diagnosis

A species of *Sylvirana* having the combination of all digit tips expanded with circummarginal grooves; skin finely granular above, smooth below; males with SVL 34.3–54.0 mm, females with SVL 42.3–55.6 mm; males with enlarged humeral glands and small, narrow nuptial pads; pineal gland visible; no oblique, triangular or teardrop-shaped marking slightly posterior to the tympanum; and flank with dark stripe below dorsolateral fold, extending from snout or behind eye to groin, but without strong demarcation between dark (upper) and light (lower) parts of flank.

#### Expanded description of lectotype

**Fig 8:**
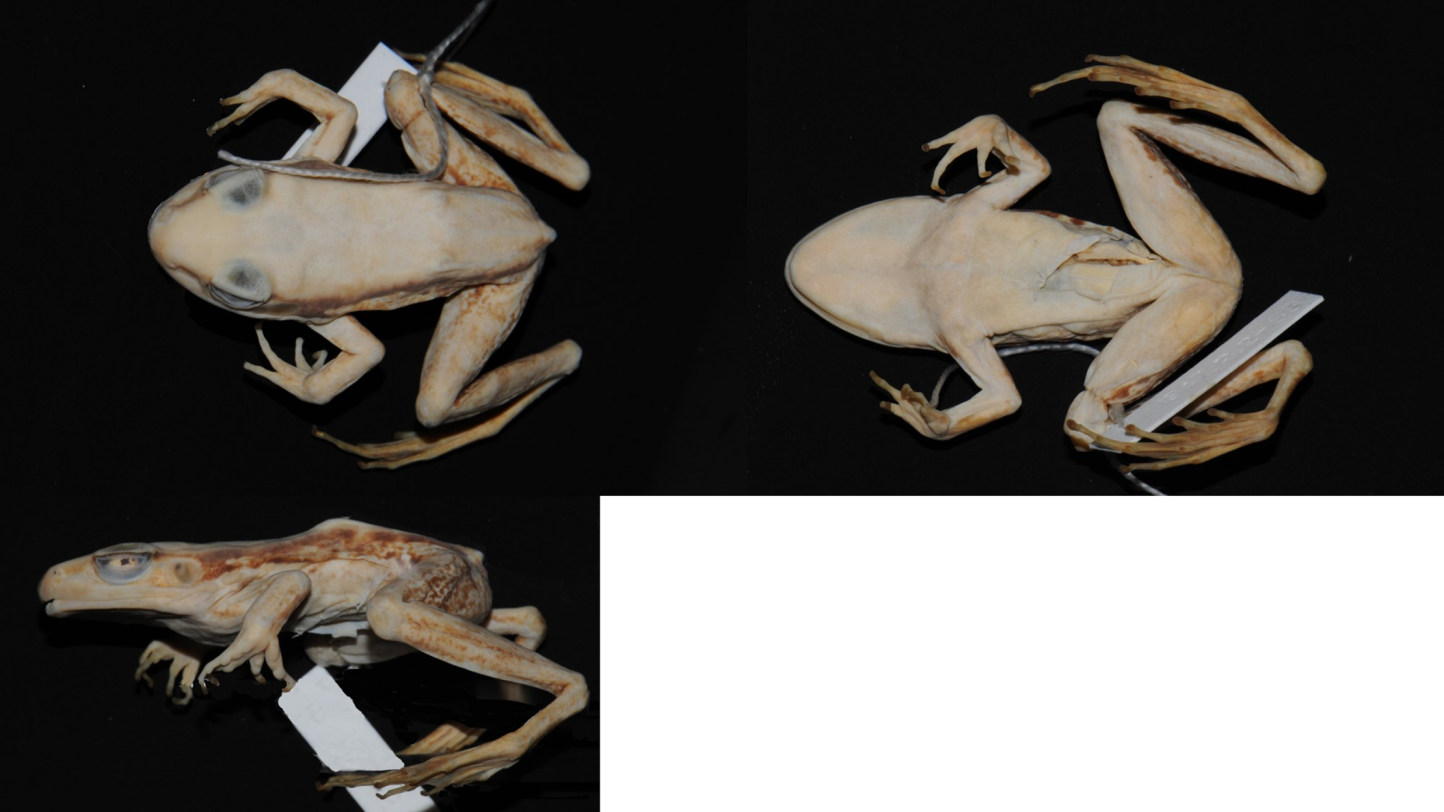
Lectotype of *Sylvirana nigrovittata*, female, BMNH 1947.2.2.99 from Mergui, Myanmar.

Female (Fig 8), SVL 50.8. Habitus moderately slender. Snout obtusely pointed in dorsal view, projecting slightly beyond lower jaw and rounded in profile. Nostril lateral, closer to tip of snout than to eye. Canthus rostralis distinct. Lores oblique and concave. Pineal body visible. Tympanum distinct, round, slightly depressed relative to skin of temporal region.

Tips of all four fingers expanded into discs with circummarginal grooves. Fingers slender. Two palmar tubercles, oval; one oval thenar tubercle. Hand webbing absent. Forelimbs slender. Hindlimbs long and moderately muscular. Toes slender. Feet with extensive webbing, Toe IV with at least one phalange free of web. Subarticular tubercles conspicuous, surfaces rounded, formula 1, 1, 2, 3, 2.

Measurements (mm): SVL 50.8, HDL 18.8, HDW 16.4, SNT 7.3, EYE 7.0, IOD 4.4, IND 5.0, TMP 4.1, SHK 26.2, TGH 23.2, HND 12.4, FTL 24.4.

#### Coloration of lectotype in preservative

Coloration mostly lost in preservative. Dorsal surface pale beige, uniformly colored. Dark coloration extending from just posterior to eye through tympanum to groin; upper half of flank darker than lower half but without strong demarcation, with dark band extending along ventral margin of dorsolateral fold, and some dark spotting on flank. Ventral surfaces immaculate cream, with no mottling or spotting. Posterior surface of thighs with dark marbling pattern on light (pale beige) background.

#### Expanded description of NCSM 79411

**Fig 9:**
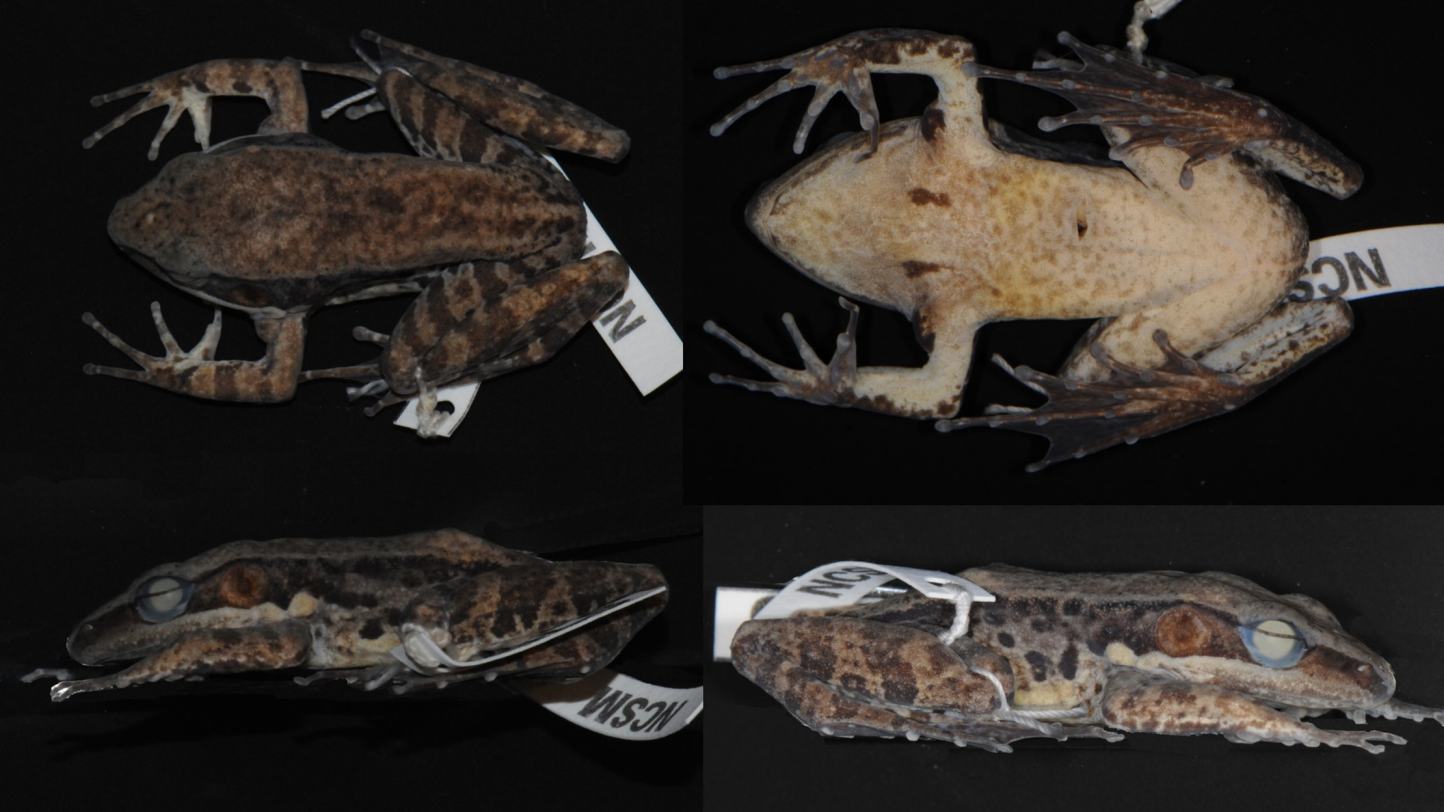
*Sylvirana nigrovittata*, male, NCSM 79411 from Paklay District, Xaignabouli Province, Laos. Photograph by BLS.

Adult male (Fig 9), SVL 50.9, Paklay District, Xaignabouli Province, Laos. Habitus moderately stocky. Head length greater than head width. Snout obtusely pointed in dorsal view, projecting beyond lower jaw and rounded in profile. Nostril lateral, closer to tip of snout than to eye. Internarial distance slightly larger than interorbital distance. Canthus rostralis distinct. Lores oblique and concave. Eye diameter 79% snout length. Interorbital distance greater than upper eyelid width (3.8 mm). Pineal body visible. Round tympanum, 78% eye diameter, slightly depressed relative to skin of temporal region, tympanic rim elevated relative to tympanum. Vomerine teeth obliquely angled, equidistant from choanae as from each other. Tongue cordiform, notched posteriorly. Small slit-like vocal sac openings near corner of jaw. No gular pouch.

Forelimbs moderately robust. Tips of all four fingers expanded into small discs with circummarginal grooves. Disc of Finger III (0.9 mm) slightly (ca. 10%) larger than phalanx diameter (0.8 mm). Relative finger lengths II < IV < I < III. Subarticular tubercles conspicuous, surfaces rounded; formula 1, 1, 2, 2; supernumerary tubercles at the base of all four fingers. Two palmar tubercles, oval, outer slightly larger than inner; oval thenar tubercle. Nuptial pad small, narrow, extending to level of subarticular tubercle. Humeral gland enlarged.

Hindlimbs long. Tips of toes expanded into discs with circummarginal grooves. Width of Toe IV disc (1.2 mm) about 20% larger than that of Finger III disc. Webbing on Toe I to base of disc, on preaxial side of Toe II to subarticular tubercle, on postaxial side of Toe II to base of disc, on preaxial side of Toe III to distal subarticular tubercle, on postaxial side of Toe III to base of disc, on Toe IV to distal subarticular tubercle, and on Toe V to base of disc. Subarticular tubercles conspicuous, surfaces rounded; formula 1, 1, 2, 3, 2. Inner metatarsal tubercle oval; outer metatarsal tubercle round.

Skin finely granular above, smooth ventrally. Skin on flank with small oval glands. Two rictal glands, anterior gland elongate and continuous with upper lip, posterior gland oval. Dorsolateral fold present. Supratympanic fold absent. Two low postorbital swellings on top of head.

Measurements (mm): SVL 50.9, HDL 20.8, HDW 19.1, SNT 8.3, EYE 6.5, IOD 4.4, IND 5.3, TMP 5.1, SHK 27.6, TGH 27.7, HND 14.7, FTL 28.3.

#### Coloration of NCSM 79411 in preservative

Dorsum medium brown with extensive dark brown mottling. Dorsal surfaces of hindlimbs and forelimbs with dark cross bands. Posterior surface of thighs with irregular pattern of dark marbling on light background. Dark line along ventral margin of dorsolateral fold. Most of flank creamy white with several dark brown spots. Lips grey-brown anteriorly, creamy white posteriorly. Rictal glands creamy white. Foot webbing mottled brown. Chin, chest, and belly creamy white with dark brown mottling. Two short, dark vertical lines just lateral to midline at base of throat. Humeral dark brown. Ventral surfaces of forelimbs creamy white, with lateral dark brown spots. Ventral surfaces of hands grey-brown. Ventral surfaces of feet dark brown.

#### Coloration of NUOL 00006 in life (based on photograph by BLS)

**Fig 10:**
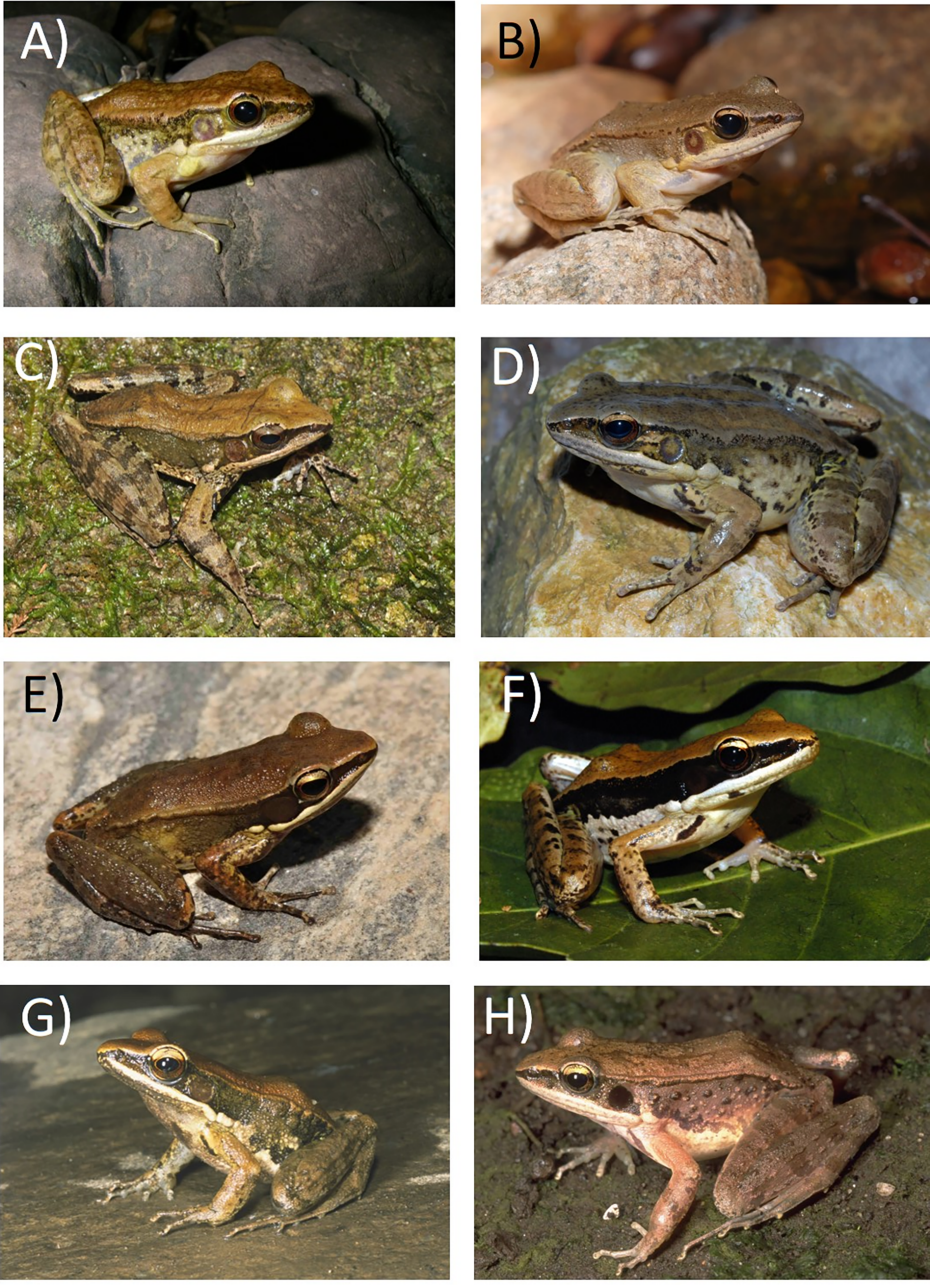
Photographs in life of A) *Sylvirana nigrovittata* (NUOL 00006), male, Phongsaly, Laos (photograph by BLS); B) *S*. *mortenseni*, unvouchered topotype from Koh Chang Island, Trat, Thailand (photograph by A. Rujirawan); C) *S*. *faber*, male, topotype (NCSM 79562), Kampong Speu, Cambodia (photograph by T. Neang); D) *S*. *montosa* sp. nov., male, paratype (NCSM 76397), Savannakhet, Laos (photograph by S. Richards); E) *S*. *annamitica* sp. nov., male, (AMNH A-181996), Quang Nam, Vietnam (photograph by D. Kizirian); F) *S*. *malayana* sp. nov., unvouchered individual from Temmengor, Malaysia (photograph by L.L. Grismer); G) *S*. *roberti* sp. nov. (CAS 229664), Tanintharyi, Myanmar (photograph by J. Vindum); and H) *S*. *lacrima* sp. nov., immature female, paratype (CAS 231229), Mandalay, Myanmar (photograph by J. Vindum).

Adult male (Fig 10A), SVL 46.5, Boun Tai District, Phongsaly Province, Laos. Dorsum red-brown. Dark stripe from tip of snout, extending posteriorly above tympanum and dorsolateral fold. Lip and rictal glands cream. Forelimb beige with faint dark crossbars. Flank mostly yellow-beige with dark spots. Hindlimb beige-brown with dark brown cross bars. Upper one-third of iris golden, lower two-thirds of iris bronze.

#### Variation

Females lack robust forearms and humeral glands (but often have a dark brown spot where humeral glands appear in males). Females are larger than males on average, but there is considerable overlap in size (Table 1). Dorsal coloration varies from medium to very dark brown. Most individuals have at least some dark spots on dorsum, but some lack obvious markings. Cross bands on dorsal surfaces of limbs usually distinct, but are faint in a few individuals. Ventral coloration varies from immaculate creamy-white to very darkly mottled. Dark markings at base of throat are present in a few specimens. Webbing on Toe IV extends to between tubercles, or reaches distal tubercle, in some specimens. Posterior rictal gland sometimes absent, but is usually visible, small, and rounded. Measurements are summarized in Table 1.

#### Molecules

**Fig 11:**
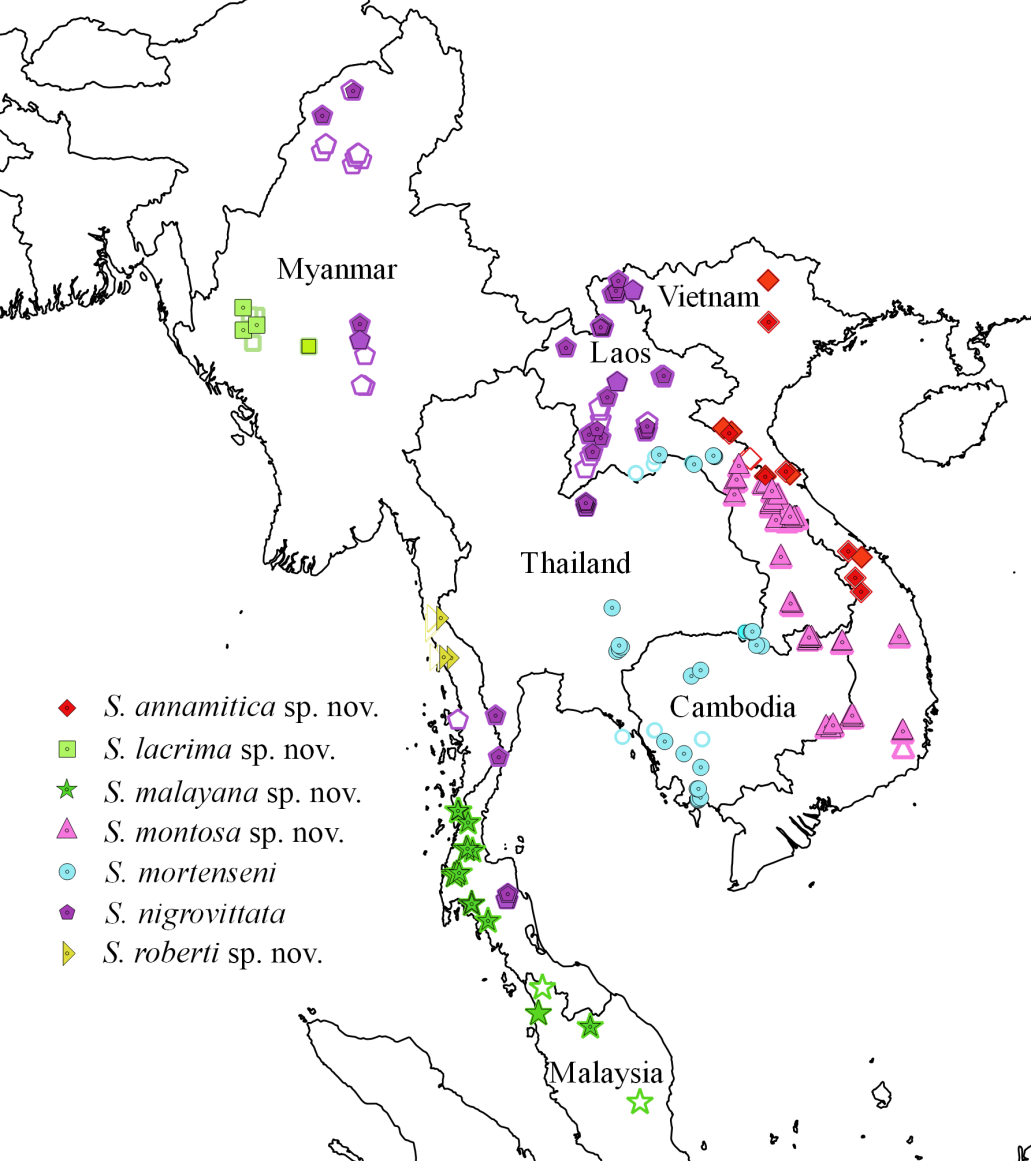
Map of sampling localities of *Sylvirana annamitica* sp. nov. (diamonds), *S*. *lacrima* sp. nov. (squares), *S*. *malayana* sp. nov. (stars), *S*. *montosa* sp. nov. (triangles), *S*. *mortenseni* (circles), *S*. *nigrovittata* (pentagons), and *S*. *roberti* sp. nov. (vertical triangles). Open symbols indicate morphological data only, shaded symbols indicate sequence data only, shaded symbols with center dots indicate both morphological and sequence data were studied.

*Sylvirana nigrovittata* was recovered in the mtDNA Bayesian analysis to be the sister taxon of *S*. *roberti* sp. nov., to which it is parapatrically distributed (Fig 11), but was found to be distantly related to *S*. *annamitica* sp. nov., the species with which it most closely overlaps in morphospace (Figs 1 and 7). *Sylvirana nigrovittata* was admixed in the nuDNA Bayesian analysis with *S*. *montosa* sp. nov., *S*. *mortenseni*, and *S*. *annamitica* sp. nov. (Fig 5), but clustered in the nuDNA phylogenetic network as a species, with close similarity to *S*. *annamitica* sp. nov. and *S*. *roberti* sp. nov. (Fig 6).

#### Distribution

*Sylvirana nigrovittata* is geographically widespread, occurring from southern peninsular Thailand through eastern Myanmar, central and northern Thailand, northern Laos to northern Vietnam (Fig 11), and presumably into extreme southern China (see Remarks below).

#### Comparisons

Males of *S*. *nigrovittata* strongly overlap with *S*. *annamitica* sp. nov. in morphospace, but can be distinguished from that species by its significantly longer TGH:SVL (0.51 ± 0.04; *S*. *annamitica* sp. nov.: 0.47 ± 0.04; t-stat = -4.11, df = 19, two-tailed p < 0.01). A lack of females (n = 5) of *S*. *annamitica* sp. nov. precluded statistical comparisons of females. *Sylvirana nigrovittata* differs from *S*. *lacrima* sp. nov. by having an irregular pattern of dark marbling on light background on the posterior surface of thighs (indistinct pattern in *S*. *lacrima* sp. nov.), and by lacking an oblique, triangular or teardrop-shaped marking slightly posterior to the tympanum (present in *S*. *lacrima* sp. nov.). *Sylvirana nigrovittata* differs from *S*. *malayana* sp. nov. in lacking a strong demarcation of dark (upper) and light (lower) coloring on the flank (present in *S*. *malayana* sp. nov.). Males of *S*. *nigrovittata* have a smaller HDW:HDL (0.91 ± 0.03) than *S*. *montosa* sp. nov. (0.94 ± 0.03; t-stat = -5.08, df = 120, p < 0.001) and a smaller HDW:SVL (0.36 ± 0.01) than *S*. *montosa* sp. nov. (0.37 ± 0.01; t-stat = 5.90, df = 106, p < 0.001). Females of *S*. *nigrovittata* have a smaller TMP:EYE (0.67 ± 0.05) than *S*. *montosa* sp. nov. (0.73 ± 0.08; t-stat = -3.65, df = 52, p < 0.001). *Sylvirana nigrovittata* differs from *S*. *mortenseni* by having males with SVL < 54 mm and females with SVL < 56 mm (the reverse in *S*. *mortenseni*), and from *S*. *roberti* sp. nov. by the presence of a pineal gland (absent in *S*. *roberti* sp. nov.). Despite the type locality of *S*. *nigrovittata* being near to the only known localities of *S*. *roberti* sp. nov., the lectotype and all all specimens referred here to *S*. *nigrovittata* have a pineal gland, but the pineal gland is lacking in all studied specimens of *S*. *roberti* sp. nov.. DFA correctly assigned males and females to *S*. *nigrovittata* 80% and 100% of the time, respectively.

#### Remarks

Our samples from Phongsaly Province, Laos, were obtained within approximately 35 air-km from the type locality of *Hylarana (Sylvirana) menglaensis* Fei, Ye and Xie, 2008 at Mengla County, Yunnan Province, China, on the border with Laos. These Phongsaly samples matched mtDNA sequences of *S*. *nigrovittata*, and we failed to recover molecular or morphological evidence for more than one species in the *S*. *nigrovittata* complex occurring in the northern reaches of Laos. *Hylarana* (*Sylvirana*) *menglaensis* was described without the use of molecular data, but was distinguished in its original description from *S*. *nigrovittata* on the basis of morphology, based on samples from Pahang, West Malaysia, which we refer to below as a separate species (*S*. *malayana* sp. nov.). As such, we synonymize *Hylarana (Sylvirana) menglaensis* Fei, Ye and Xie, 2008 with *S*. *nigrovittata* [50].

The type locality of *Hylarana (Sylvirana) hekouensis* Fei, Ye and Jiang, 2008 at Nanxi, Hekou County, Yunnan Province, China, near the border with Vietnam, lies almost equidistant between our nearest sampled locality of *S*. *nigrovittata* at Dien Bien Province, Vietnam (approximately 180 air-km away) and our northernmost sample of *S*. *annamitica* sp. nov. (below) at Bac Kan Province, Vietnam (approximately 170 air-km away). No molecular data were provided in the original description of *H*. (*S*.) *hekouensis* [57]. Six males in the type series of *H*. (*S*.) *hekouensis* had SVL 34.3–41.0 mm, with mean 38.2 mm (no measure of standard error provided; Fei et al. 2008 [57]), 11 males of *S*. *nigrovittata* that we examined from Dien Bien had SVL 34.3–44.8 mm, with mean 39.4 mm, and 16 males of *S*. *annamitica* sp. nov. (below) from throughout its known range (only a single specimen was available from Bac Kan) had SVL 40.2–52.0 mm, with mean 45.0 mm. Thus, on the basis of size, the Hekou specimens are more similar to those from Dien Bien (= *S*. *nigrovittata*) than to those described here as *S*. *annamitica* sp. nov. Moreover, a complete mitochondrial genome of *H*. *hekouensis* (GenBank accession number KX021954) provided by Chen et al. [59] is genetically more similar to our outgroup sample of *S*. *maosonensis* than it is to members of the *S*. *nigrovittata* complex (analysis not shown), but unfortunately the provenance of this GenBank sequence of *H*. *(S*.*) hekouensis* is not reported [59]. We conclude that the taxonomic status of *Hylarana (Sylvirana) hekouensis* Fei, Ye and Jiang, 2008 remains uncertain, but it is not likely to be attributable to any species being described as new herein (the closest new taxon would be *S*. *annamitica* sp. nov.). To draw attention to this taxonomic problem, we provisionally synonymize *Hylarana (Sylvirana) hekouensis* Fei, Ye and Jiang, 2008 with *S*. *nigrovittata* [50] on the basis of our, admittedly, very limited morphological (SVL) evidence, and urge further investigation into the taxonomic status of *H*. *(S*.*) hekouensis* using vouchered, known locality material.

***Sylvirana mortenseni* (Boulenger, 1903)**

*Rana mortenseni* Boulenger, 1903: 219 [25].

*Rana* (*Hylorana*) *mortenseni* Boulenger, 1920: 123 [52].

*Rana mortenseni* Smith, 1921: 434 [24]; Stuart and Emmett, 2006: 10 [60].

*Hylarana mortenseni* Chen, Murphy, Lathrop, Ngo, Orlov, Ho, and Somorjai, 2005: 237 [54]; Che, Pang, Zhao, Wu, Zhao and Zhang, 2007: 3 [55].

*Rana (Sylvirana) mortenseni* Dubois, 1992: 326 [56]; Ohler, Swan, and Daltry, 2002: 474 [28].

*Hylarana (Sylvirana) mortensensi* Fei, Ye, Jiang, and Xie, 2008: 204 [57].

*Rana nigrovittata* Smith, 1922: 212 (part) [26]; Matsui, Nishikawa, Khonsue, Panha, and Nabhitabhata, 2001: 15 [27].

*Sylvirana mortenseni* Frost, Grant, Faivovich, Bain, Haas, Haddad, de Sá, Channing, Wilkinson, Donnellan, Raxworthy, Campbell, Blotto, Moler, Drewes, Nussbaum, Lynch, Green, and Wheeler, 2006: 370 [31]; Oliver, Prendini, Kraus, and Raxworthy, 2015: 191 [29].

#### Lectotype

Designated herein, Zoological Museum University of Copenhagen (ZMUC) R072735, adult male, Thailand, Trat Province, Koh Chang Island, coll. 15 January 1900 by Dr. Mortensen. The syntype series also included specimens from Yado and Thao, Upper Burma (Myanmar) [25], but the allocation of the Burmese specimens to *S*. *mortenseni* seems unlikely based on morphology and verified range of this species (below).

#### Expanded diagnosis

A species of *Sylvirana* having the combination of all digit tips expanded with circummarginal grooves; skin finely granular above, smooth below; males with SVL 46.0–80.9 mm, females with SVL 47.7–69.2 mm; males with enlarged humeral glands and well-developed nuptial pads; pineal gland visible; no oblique, triangular or teardrop-shaped marking slightly posterior to the tympanum; and flank with dark stripe below dorsolateral fold, extending from snout or behind eye to groin, but without strong demarcation between dark (upper) and light (lower) parts of flank.

#### Description of lectotype

**Fig 12:**
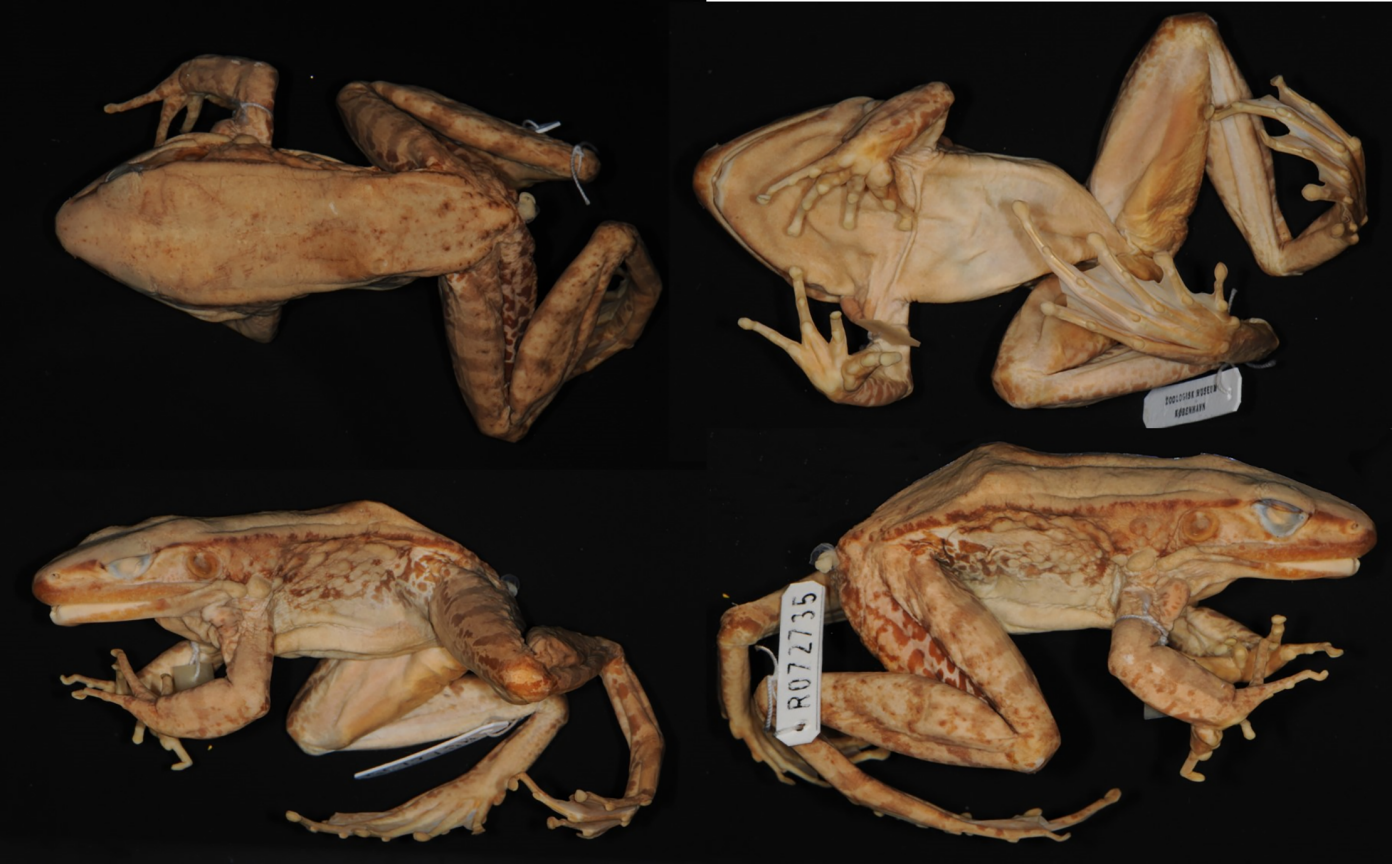
Lectotype of *Sylvirana mortenseni*, male, ZMUC R072735 from Koh Chang, Trat Province, Thailand.

Adult male (Fig 12), SVL 80.9 mm. Habitus moderately slender. Head length subequal to head width. Snout pointed in dorsal view. Snout projecting beyond lower jaw in lateral view, bluntly rounded. Nostril lateral, nearer to tip of snout than to eye. Internarial distance subequal to interorbital distance. Canthus rostralis distinct. Lores oblique and slightly concave. Eye diameter 69% of snout length. Interorbital distance greater than upper eyelid width. Pineal body visible. Tympanum distinct, round, 69% of eye diameter, slightly depressed relative to skin of temporal region, tympanic rim elevated relative to tympanum. Vomerine teeth obliquely angled, equidistant from choanae as from each other. Tongue cordiform, notched posteriorly. No vocal slit visible, no gular pouch.

Forelimbs robust. Tips of all four fingers slightly expanded into discs, without circummarginal grooves. Diameter of Finger III disc (2.2 mm) approximately 50% greater than phalanx diameter (1.4 mm). Fingers slender. Relative finger lengths II < IV < I < III. Distal portion of Finger III reaches level of Finger IV disc. Webbing absent. Subarticular tubercles conspicuous, surfaces rounded; formula 1, 1, 2, 2. One supernumerary tubercle at base of each finger. Two oval palmar tubercles in contact; one round thenar tubercle. Well-developed nuptial pad on Finger I, on dorsal surface to level of subarticular tubercle. Humeral glands enlarged.

Hindlimbs long and moderately muscular. Toes slender. Tips of toes expanded into discs, with circummarginal grooves, width of Toe IV disc 42% that of tympanum. Web on Toe I to base of disc, on preaxial side of Toe II to subarticular tubercle, on postaxial side of Toe II to base of disc, on preaxial side of Toe III to subarticular tubercle, on postaxial side of Toe III to base of disc, on preaxial side of Toe IV distal to penultimate subarticular tubercle, on postaxial side of Toe IV to distal subarticular tubercle, on Toe V to base of disc. Subarticular tubercles conspicuous, surfaces rounded; formula 1, 1, 2, 3, 2. Inner metatarsal tubercle oval; outer metatarsal tubercle round.

Skin finely granular. Supratympanic fold present. Two rictal glands; anterior gland elongate and continuous with upper lip, posterior gland round. Weak dorsolateral fold. Dorsal part of flank with medium-large oval glands, ventral part of flank mostly smooth. Two low postorbital swellings on top of head.

Measurements (mm): SVL 80.9, HDL 31.3, HDW 31.8, SNT 12.9, EYE 8.9, IOD 7.9, IND 8.0, TMP 6.2, SHK 44.8, TGH 43.5, HND 21.7, FTL 45.0

#### Coloration of lectotype in preservative

Coloration mostly lost in preservative. Dorsum beige with irregular light brown markings. Canthus and lip brown, lores beige. Ventral side of dorsolateral fold medium brown, fading to beige dorsally. Mostly immaculate, creamy-beige throat, belly and ventral surface of thighs. Posterior surface of thighs with irregular pattern of dark marbling on light background.

#### Coloration of topotype in life (based on photograph by Attapol Rujirawan)

Unvouchered individual (Fig 10B), Koh Chang Island, Trat Province, Thailand, photographed 29 January 2011. Dorsum and dorsolateral fold brown with few small dark brown spots. Canthus rostralis dark brown, lores brown. Temporal region yellow-beige. Limbs pale brown or beige. Hindlimbs with medium brown crossbands, and yellow coloration on anterior and posterior surfaces. Lip creamy white. Upper one-quarter of iris bronze, lower three quarters of iris dark brown. Chin and chest creamy white with brown mottling. Flank brown, fading to creamy coloration ventrally.

#### Variation

Females lack robust forearms and humeral glands (but often have a dark brown spot where humeral glands appear in males). Males and females greatly overlap in SVL [mean SVL is not significantly different (62.7 ± 8.7 and 60.3 ± 6.2 for males and females, respectively; t-stat = -1.28, df = 38, p > 0.20)], but males can achieve larger SVL (up to 81 mm, while females reach only 69 mm). Males have larger HDW:HDL ratio (0.94 ± 0.04; t-statistic = 2.92, df = 38, p < 0.01) and HDW:SVL ratio (0.37 ± 0.01; t-statistic = 7.33, df = 28, p < 0.001) than females (0.92 ± 0.03 and 0.34 ± 0.01, respectively). Circummarginal grooves are not visible on the fingers of the lectotype, but are distinctly visible in all recently preserved specimens.

Dorsal coloration varies from medium to dark brown, with or without very small dark spots. Mostindividuals have a thick dark line along ventral margin of the dorsolateral fold. Most individuals have dark coloration on upper regions of flank, fading to pale coloration ventrally, but the transition from dark to light coloration on the flank is extremely variable, but never with a sharp demarcation: some individuals have minimal dark coloration above and mostly light-colored flanks, while other individuals have mostly dark-colored flanks. Most individuals have medium to large dark spots on the flank.

Ventral coloration is extremely variable, ranging from creamy white to nearly black with dark mottling. Some individuals have pale throats or bellies, with the remaining ventral surfaces heavily mottled, while other individuals have consistent coloration across all ventral surfaces. Underside of thighs may be cream or heavily stippled so as to appear grey or mottled. Most often dark ventral coloration is stippled or mottled, rather than concentrated in distinct dark spots. Spots on rear of thighs vary from about 25% to subequal diameter of the tympanum. Posterior rictal gland is absent or only weakly visible in some individuals. Lip coloration varies from creamy white to very dark brown.

Mature males have enlarged forearms, humeral glands, and faint nuptial pads that are sometimes difficult to see with the naked eye, but are visible under a dissecting microscope. Regardless of sexual maturity, males always have dark spots at the humeral gland location. Vocal slits are visible in some males, and presence/absence does not seem to correspond with collection locality or body size. Measurements are summarized in Table 1.

#### Molecules

*Sylvirana mortenseni* contained two mtDNA subclades, one containing samples from southwestern Cambodia and southeastern Thailand, and one containing samples from central Cambodia, southern Laos, and eastern Thailand (Fig 3). *Sylvirana mortenseni* was recovered in the mtDNA Bayesian analysis to be the sister taxon to a clade containing *S*. *annamitica* sp. nov. and *S*. *montosa* sp. nov. (Fig 1), the latter of which it also slightly overlaps with in morphospace (Fig 7). *Sylvirana mortenseni* was admixed in the nuDNA Bayesian analysis with *S*. *montosa* sp. nov., *S*. *nigrovittata*, and *S*. *annamitica* sp. nov. (Fig 5), but clustered in the nuDNA phylogenetic network with *S*. *montosa* sp. nov. (Fig 6).

#### Distribution

*Sylvirana mortenseni* occurs from southeastern and eastern Thailand through central Laos and southwestern and central Cambodia (Fig 11). *Sylvirana mortenseni* occurs in syntopy with *S*. *faber* in southwestern Cambodia [60].

#### Comparisons

*Sylvirana mortenseni* can be distinguished from *S*. *annamitica* sp. nov. by having significantly larger body size (Table 1). *Sylvirana mortenseni* can be distinguished from *S*. *lacrima* sp. nov. by having an irregular pattern of dark marbling on light background on the posterior surface of thighs (indistinct pattern in *S*. *lacrima* sp. nov.) and by lacking an oblique, triangular or teardrop-shaped marking slightly posterior to the tympanum (present in *S*. *lacrima* sp. nov.). *Sylvirana mortenseni* differs from *S*. *malayana* sp. nov. by the lack of a strong demarcation between dark and pale coloration on the flank. *Sylvirana mortenseni* strongly overlaps with *S*. *montosa* sp. nov. in morphospace (Fig 7), but males are approximately 8% larger than that species (Table 1), while females of these two species do not significantly differ in SVL (t-stat = 0.68, df = 27, p = 0.50). However, females of this species have a smaller FTL:SVL (0.49 ± 0.03) than *S*. *montosa* sp. nov. (0.50 ± 0.02; t-stat = -2.21, df = 27, p = 0.04). *Sylvirana mortenseni* is significantly larger than *S*. *nigrovittata*, and the presence of a pineal gland distinguishes this species from *S*. *roberti* sp. nov. DFA correctly assigned males and females of this species 94% of the time.

***Sylvirana faber* (Ohler, Swan, and Daltry, 2002)**

*Rana (Sylvirana) faber* Ohler, Swan, and Daltry, 2002: 475 [28]; Chuaynkern, Ohler, Inthara, Kumtong, and Dubois, 2004: 2 [61].

*Rana faber* Stuart, 2005: 463 [62]; Stuart and Emmett, 2006: 9 [60].

*Hylarana faber* Chen, Murphy, Lathrop, Ngo, Orlov, Ho, and Somorjai, 2005: 237 [54]; Che, Pang, Zhao, Wu, Zhao and Zhang, 2007: 3 [55].

*Hylarana (Sylvirana) faber* Fei, Ye, Jiang, and Xie, 2008: 204 [57].

*Sylvirana faber* Frost, Grant, Faivovich, Bain, Haas, Haddad, de Sá, Channing, Wilkinson, Donnellan, Raxworthy, Campbell, Blotto, Moler, Drewes, Nussbaum, Lynch, Green, and Wheeler, 2006: 370 [31]; Oliver, Prendini, Kraus, and Raxworthy, 2015: 191 [29].

#### Holotype

MNHN 2001.0261 (not examined by us), adult male, SVL 59.4 mm, Cambodia, Kampong Speu Province, Phnom Aural Wildlife Sanctuary, Phnom Aural, 12.0093 N, 103.2337 E [28].

#### Expanded diagnosis

A species of *Sylvirana* having the combination of all digit tips expanded with circummarginal grooves; males with SVL 51.0–79.3 mm, females with SVL 76.6–89.2 mm [60, 61]; distinct dorsolateral folds; scattered asperities on dorsum; brown dorsum, usually with light-colored flecking on back; males with enlarged forearms; males with humeral glands that are externally visible but do not form a conspicuous bulge; pineal gland visible; and no oblique, triangular or teardrop-shaped marking slightly posterior to the tympanum.

#### Expanded description of topotype NCSM 79562

**Fig 13:**
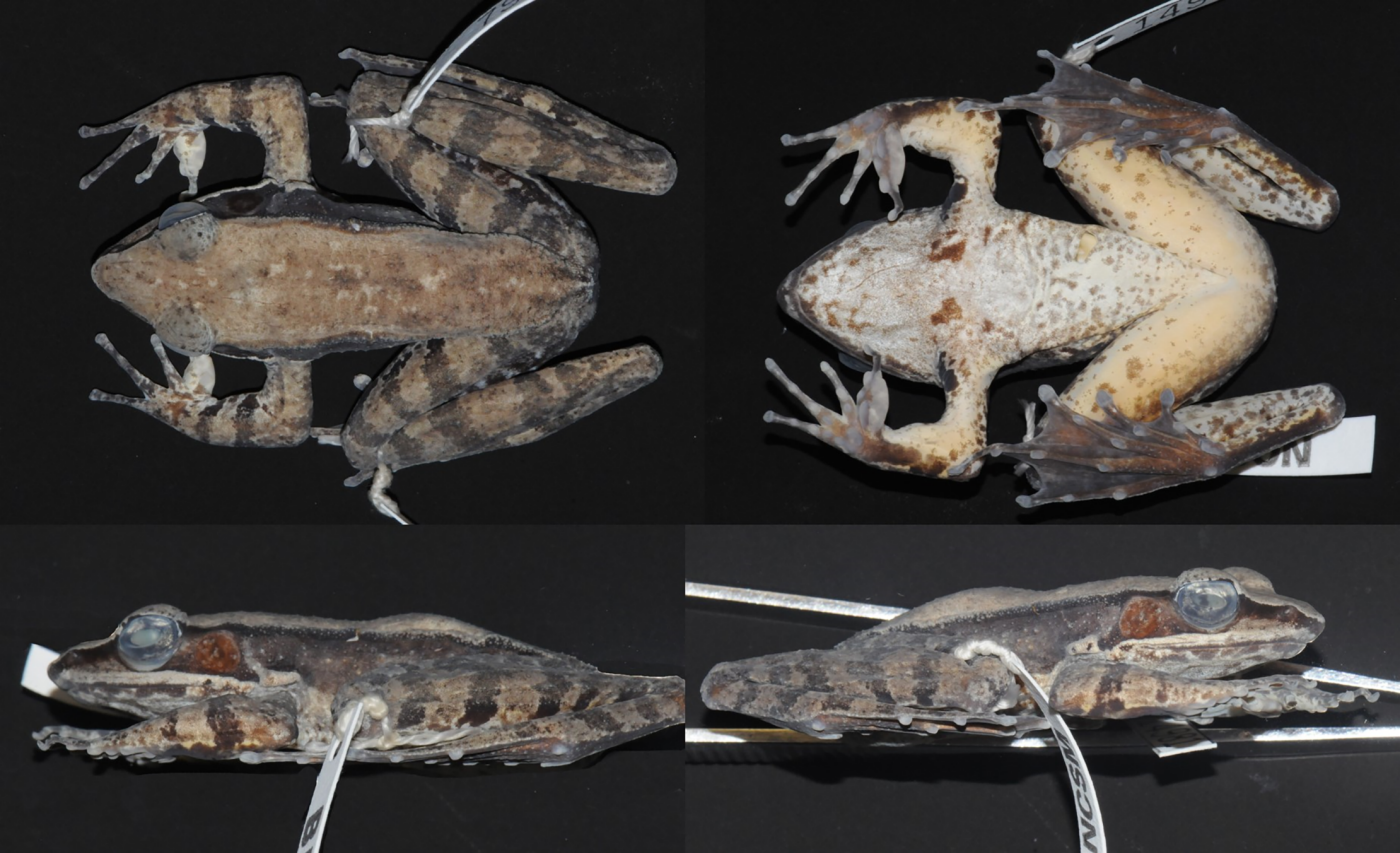
Topotype of *Sylvirana faber*, male, NCSM 79562, from Phnom Aural, Kampong Speu Province, Cambodia. Photograph by T. Neang.

Adult male (Fig 13), SVL 57.9 mm, Phnom Aural, Kampong Speu Province, Cambodia. Habitus moderately stocky. Head length greater than head width. Snout obtusely pointed in dorsal view, projecting beyond lower jaw and rounded in profile. Nostril lateral, closer to tip of snout than to eye. Internarial distance slightly larger than interorbital distance. Canthus rostralis distinct. Lores oblique and concave. Eye diameter 86% snout length. Interorbital distance greater than upper eyelid width (5.0 mm). Pineal body visible. Round tympanum, 64% eye diameter, slightly depressed relative to skin of temporal region, tympanic rim elevated relative to tympanum. Vomerine teeth obliquely angled, equidistant from choanae as from each other. Tongue cordiform, notched posteriorly. Small slit-like vocal sac openings near corner of jaw. No gular pouch.

Forelimbs robust. Tips of all four fingers expanded into small discs with circummarginal grooves. Disc of Finger III (0.94 mm) only slightly larger (about 8%) than phalanx (0.87 mm). Relative finger lengths II < IV < I < III. Subarticular tubercles conspicuous, surfaces rounded; formula 1, 1, 2, 2; supernumerary tubercles at the base of all four fingers. Two palmar tubercles, oval, outer slightly longer but more slender than inner; oval thenar tubercle. Nuptial pad well developed, to level of subarticular tubercle. Humeral gland weakly developed.

Hindlimbs long. Tips of toes expanded into discs with circummarginal grooves. Width of Toe IV disc (1.60 mm) about 70% larger than that of Finger III disc. Webbing on Toe I to base of disc, on preaxial side of Toe II to subarticular tubercle, on postaxial side of Toe II to base of disc, on preaxial side of Toe III to distal subarticular tubercle, on postaxial side of Toe III to base of disc, on Toe IV to distal subarticular tubercle, and on Toe V to base of disc. Subarticular tubercles conspicuous, surfaces rounded; formula 1, 1, 2, 3, 2. Inner metatarsal tubercle oval; outer metatarsal tubercle round.

Skin finely granular above, smooth ventrally. Skin on flank with small glands. One oval rictal gland. Dorsolateral fold present. Supratympanic fold absent. Two low postorbital swellings on top of head.

Measurements (mm): SVL 57.9, HDL 21.5, HDW 20.7, SNT 8.7, EYE 7.5, IOD 5.2, IND 5.6, TMP 4.8, SHK 39.0 TGH 35.7, HND 14.9, FTL 33.8.

#### Coloration of topotype NCSM 79562 in preservative

Dorsum medium brown with some dark brown mottling. Dorsal surfaces of limbs with distinct cross bands. Posterior surface of thighs with irregular pattern of dark marbling on light background. Dark line extending along ventral margin of dorsolateral fold, but most of flank medium- to dark-brown with some pale spots. Anterior portion of lip grey-brown, creamy white posteriorly. Rictal gland creamy white. Webbing mottled brown. Chin, chest, and belly creamy white with some dark brown mottling. Two short, dark vertical stripes just lateral to midline at base of throat. Humeral glands moderately enlarged and dark brown in color. Ventral surfaces of forelimbs creamy white with dark brown spots laterally. Ventral surfaces of hands grey-brown. Ventral surfaces of feet dark brown.

#### Coloration of topotype NCSM 79562 in life (based on photograph by T. Neang)

Dorsum red-brown. Dark stripe from tip of snout, extending posteriorly, above and below tympanum. Lip and rictal gland cream. Dorsal surfaces of forelimbs and hindlimbs beige-brown with dark crossbands. Flanks mostly greenish brown with pale spots. Upper one-third of iris golden, lower two-thirds bronze (Fig 10C).

#### Variation

Females are distinctly larger than males [60, 61], and lack visible humeral glands.

#### Molecules

*Sylvirana faber* was recovered in the mtDNA Bayesian analysis to be the sister taxon to a clade containing all other members of the *S*. *nigrovittata* complex except *S*. *lacrima* sp. nov. (Fig 1). *Sylvirana faber* was also recovered as a distinct lineage in the nuDNA Bayesian analysis and nuDNA phylogenetic network (Figs 5 and 6).

#### Distribution

*Sylvirana faber* is restricted to the uplands of southwestern Cambodia (Kampong Speu and Kampot Provinces) and adjacent southeastern Thailand (Chantaburi Province) [28, 60–62]. *Sylvirana faber* occurs in syntopy with *S*. *mortenseni* [60].

#### Comparisons

*Sylvirana faber* differs from *S*. *annamitica* sp. nov., *S*. *lacrima* sp. nov., *S*. *malayana* sp. nov., *S*. *montosa* sp. nov., *S*. *mortenseni*, *S*. *nigrovittata*, and *S*. *roberti* sp. nov., by having females with SVL ≥ 70 mm (females with SVL ≤ 70 in *S*. *annamitica* sp. nov., *S*. *lacrima* sp. nov., *S*. *malayana* sp. nov., *S*. *montosa* sp. nov., *S*. *mortenseni*, *S*. *nigrovittata*, and *S*. *roberti* sp. nov.). *Sylvirana faber* further differs from *S*. *annamitica* sp. nov., *S*. *malayana* sp. nov., *S*. *montosa* sp. nov., *S*. *mortenseni*, *S*. *nigrovittata*, and *S*. *roberti* sp. nov. by having males with humeral glands that are externally visible but do not form a conspicuous bulge (humeral glands form a conspicuous bulge in *S*. *annamitica* sp. nov., *S*. *malayana* sp. nov., *S*. *montosa* sp. nov., *S*. *mortenseni*, *S*. *nigrovittata*, and *S*. *roberti* sp. nov.). *Sylvirana faber* further differs from *S*. *lacrima* sp. nov. by lacking a small triangle or teardrop-shaped marking slightly posterior to the tympanum (present in *S*. *lacrima* sp. nov.) and having an irregular pattern of dark marbling on light background on posterior surface of thighs (indistinct pattern in *S*. *lacrima* sp. nov.).

#### Remarks

Stuart and Emmett [60] reviewed errors in the SVL of the holotype male and the adult female paratypes reported in the original description [28]. Chuaynkern et al. [61] referred one of the male paratypes to another species, and reported different sexes for specimens in the type series.

***Sylvirana montosa* sp. nov.**

*Zoobank ID*: urn:lsid:zoobank.org:act:223986CF-2C65-426B-BE4A-66DF0E87C255

*Rana nigrovittata* Smith, 1921: 433 (part) [24]; Smith, 1922: 212 (part) [26]; Inger, Orlov & Darevsky, 1999: 21 [63]; Stuart, Sok & Neang, 2006: 141 [64].

#### Holotype

FMNH 255417, adult male, Laos, Khammouan Province, Boualapha District, Hin Nam No National Protected Area (formerly Hin Nam No National Biodiversity Conservation Area), Phou Khaonok Mountain, 17.3833 N, 105.7500 E, 545 m elev., coll. 18 February 1998 by Bryan L. Stuart.

#### Paratypes

FMNH 255415 (adult male), Laos, Khammouan Province, Boualapha District, Hin Nam No National Protected Area, 17.5000 N, 105.8500 E, 200 m elev., coll. 16 February 1998 by Bryan L. Stuart. FMNH 255418 (adult male), Laos, Khammouan Province, Boualapha District, Hin Nam No National Protected Area, 17.3333 N, 105.6833 E, 500 m elev., coll. 25 February 1998 by Bryan L. Stuart. FMNH 255419 (adult male), Laos, Khammouan Province, Boualapha District, Hin Nam No National Protected Area, 17.2833 N, 105.6833 E, 240 m elev., coll. 01 March 1998 by Bryan L. Stuart.

FMNH 255420 (adult male), FMNH 255421–26 (six adult females), Laos, Khammouan Province, Nakai District, Phou Hin Poun National Protected Area (formerly Phou Hin Poun National Biodiversity Conservation Area), 17.8500 N, 104.8667 E, 220 m elev., coll. 17–18 March 1998 by Bryan L. Stuart and Tanya Chan-ard. FMNH 255427 (adult female), Laos, Khammouan Province, Nakai District, Phou Hin Poun National Protected Area, 17.8833 N, 104.9167 E, 570 m elev., coll. 23 March 1998 by Bryan L. Stuart and Tanya Chan-ard. FMNH 255428 (adult male), Laos, Khammouan Province, Thakhek District, Phou Hin Poun National Protected Area, 17.5500 N 104.8667 E, 200 m elev., coll. 01 April 1998 by Bryan L. Stuart.

FMNH 258117, 258119, 258121, 258198, 258200–02 (seven adult males), FMNH 258118, 258120 (two adult females), Laos, Champasak Province, Pakxong District, Dong Hua Sao National Protected Area (formerly Dong Hua Sao National Biodiversity Conservation Area), slope of Bolaven Plateau, near 15.0467 N, 106.1792 E, 400 m elev., coll. 16–18 September 1999 by Bryan L. Stuart and Harold Heatwole.

NCSM 76395 (adult male), Laos, Savannakhet Province, Vilabouli District, Sepon Mining Tenement, Ban Houay Hong Village, Houay Hoong Stream, 17.0445 N, 106.1262 E, 254 m elev., coll. 04 December 2008 by Bryan L. Stuart, Stephen J. Richards, Somphouthone Phimmachak, and Niane Sivongxay. NCSM 76397 (adult male), NCSM 76398 (adult female), Laos, Savannakhet Province, Vilabouli District, Sepon Mining Tenement, Ban Kalai Village, Houay Kalai Stream, 17.0118 N, 106.2201 E, 353 m elev., coll. 06 December 2008 by Bryan L. Stuart, Stephen J. Richards, Somphouthone Phimmachak, and Niane Sivongxay.

FMNH 253792–94 (three adult males), FMNH 253795–96, (two adult females), Vietnam, Gia Lai Province, An Khe District, 20 km NW of Kannack Town, Buon Luoi Village, 14.3333 N, 108.6000 E, 700–750 m elev., coll. 22 March 1995 and 04 February 1998 by Ilya Darevsky and Nikolai L. Orlov. BMNH 1921.4.1.224–225 (two adult females), Vietnam, Lam Dong Province, Langbian Plateau, Daban, coll. in 1917 by Malcolm A. Smith and C. Boden Kloss.

FMNH 261971–73, 261975 (four adult males), Cambodia, Mondolkiri Province, Pichrada District, Phnom Nam Lyr Wildlife Sanctuary, Phnom Nam Lyr Mountain, near 12.5378 N, 107.5333 E, 600 m elev., coll. 16–17 June 2000 by Bryan L. Stuart. FMNH 261978 (adult male), Cambodia, Mondolkiri Province, Pichrada District, Phnom Nam Lyr Wildlife Sanctuary, Phnom Nam Lyr Mountain, near 12.4969 N, 107.4925 E, 700 m elev., coll. 22 June 2000 by Bryan L. Stuart.

#### Referred material

Additional specimens are referred to the new species in S1 and S2 Tables.

#### Etymology

The specific epithet *montosa* L. means mountainous, in reference to the new species being associated with hilly or mountainous topography.

#### Diagnosis

A species of *Sylvirana* having the combination of all digit tips expanded with circummarginal grooves; skin finely granular above, smooth below; males with SVL 44.7–74.7 mm, females with SVL 49.1–69.0 mm; males with enlarged humeral glands and small, narrow nuptial pads; thin dark stripe extending below dorsolateral fold from posterior margin of eye to groin, but without strong demarcation between dark (upper) and light (lower) parts of flank; and no oblique, triangular or teardrop-shaped marking slightly posterior to the tympanum.

#### Description of holotype

**Fig 14:**
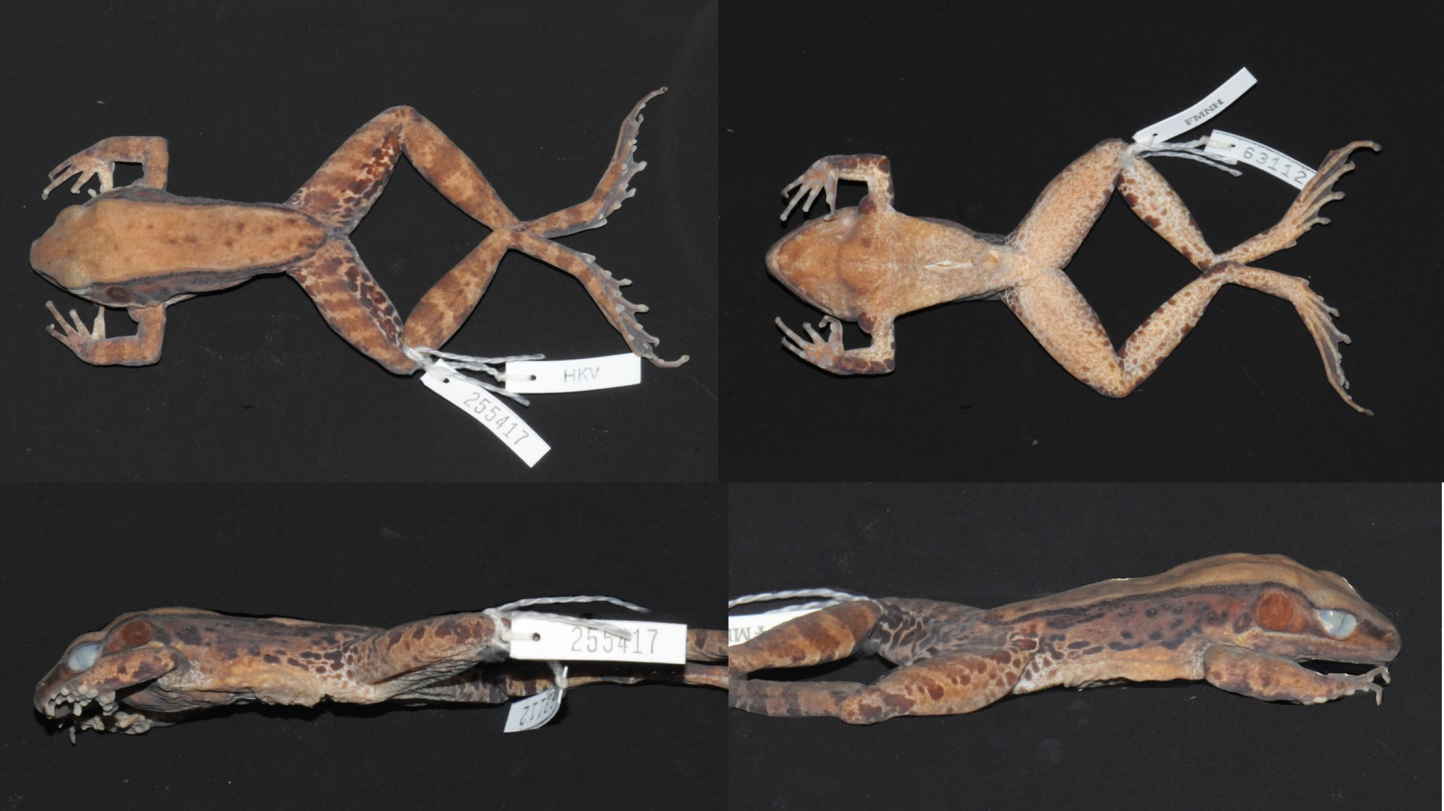
Holotype of *Sylvirana montosa* sp. nov., male, FMNH 255417 from Hin Nam No National Protected Area, Khammouan Province, Laos.

Adult male (Fig 14), 61.1 mm SVL. Habitus moderately slender. Head length greater than head width. Snout obtusely pointed in dorsal view, projecting beyond lower jaw and rounded in profile. Nostril lateral, closer to tip of snout than to eye; internarial distance greater than interorbital distance. Canthus rostralis distinct, lores oblique and concave. Eye diameter 93% snout length; interorbital distance lesser than upper eyelid width (6.3 mm). Pineal body not visible. Round tympanum 72% eye diameter, slightly depressed relative to skin of temporal region, tympanic rim elevated relative to tympanum. Vomerine teeth obliquely angled, equidistant from choanae as from each other. Tongue cordiform, notched posteriorly. Small slit-like vocal sac openings near corner of jaw. No gular pouch.

Forelimbs robust. Tips of fingers expanded into small discs with circummarginal grooves, width of Finger III disc (1.04 mm) only slightly greater than width of phalanx (0.95 mm), and about 18% diameter of tympanum. Relative finger lengths II < I < IV < III. Webbing absent. Subarticular tubercles conspicuous and rounded; formula: 1, 1, 2, 2. One supernumerary tubercle at the base of each finger; two oval palmar tubercles; one oval thenar tubercle. Humeral glands enlarged. Nuptial pad present.

Hindlimbs long. Tips of toes expanded into discs with circummarginal grooves, width of Toe IV disc (1.35 mm) 23% that of tympanum. Webbing on Toe I to base of disc, on preaxial side of Toe II to just distal of subarticular tubercle, on postaxial side of Toe II to base of disc, on preaxial side of Toe III to distal subarticular tubercle, on postaxial side of Toe III to base of disc, on preaxial side of Toe IV to distal-most joint, on postaxial side of Toe IV to distal-most joint, and on Toe V to base of disc. Subarticular tubercles conspicuous, surfaces rounded; formula: 1, 1, 2, 3, 2. Inner metatarsal tubercle elongate; outer metatarsal tubercle round.

Skin finely granular dorsally, smooth ventrally, slightly glandular on flanks. Supratympanic fold extending from dorsolateral fold to rictal gland, along posterior margin of tympanum. Two rictal glands: anterior elongate and continuous with upper lip, posterior gland round. Distinct dorsolateral fold. Two low postorbital swellings on top of head.

Measurements (mm): SVL 61.1, HDL 26.1, HDW 24.2, SNT 8.9, EYE 8.2, IOD 5.0, IND 6.0, TMP 5.9, SHK 36.1, TGH 29.3, HND 16.0, FTL 33.2.

#### Coloration of holotype in preservative

Medium brown dorsally with a few dark brown spots. Dorsal surfaces of hindlimbs with distinct crossbars, less apparent but still present on dorsal surfaces of forelimbs. Posterior surface of thighs with irregular pattern of dark marbling on light background. Dorsolateral fold dark brown. Flanks darker towards dorsum than towards belly, with dark brown spots. Lip grey-brown anteriorly and beige posteriorly. Humeral glands dark brown. Chin mottled medium-dark; chest, belly, and underside of thighs pale with brown mottling. Webbing and bottom of feet brown; palms pale to medium brown. Lateral and undersides of forelimbs spotted, proximal sides creamy white with brown mottling.

#### Coloration of paratype NCSM 76397 in life (based on photograph by Stephen Richards)

Adult male (Fig 10D), SVL 61.7, Vilabouli District, Savannakhet Province, Laos. Dorsum medium brown with small to medium-sized darker spots. Dark stripe starting at snout extending posteriorly through eye and above tympanum to the groin, broken posterior to axilla, continuing along dorsolateral fold. Flanks cream with dark brown spots. Forelimbs beige with some dark markings. Hindlimbs darker beige with dark brown cross bars, and dark spots and some creamy-yellow coloration on the anterior portion of flank. Lip grey fading to white posteriorly, rictal glands white. Upper one-quarter of iris golden, lower part bronze.

#### Variation

Females lack robust forearms or humeral glands (but often have a dark brown spot where humeral glands appear in males). Females (SVL 59.1 ± 5.2) not significantly larger than males (SVL 57.7 ± 6.2; t-stat = -1.13; df = 82; p = 0.26; Table 1). Dorsal coloration varies from medium-to-dark brown with some dark mottling or spots. Lips grey, brown, or cream, or some combination of darker color anteriorly fading to cream posteriorly. Coloration on flanks usually fading ventrally into pale belly coloration and with distinct dark spots, but a few individuals have very dark flanks with distinct dark spots. Ventral coloration ranges from immaculate creamy-white to very darkly mottled, but throat never lighter in coloration than chest and belly. A few specimens have small faint stripes just lateral to the midline at the base of the throat. Nuptial pads in males are narrow, but clearly visible under a dissecting microscope. Posterior rictal gland may be ovoid or round. Pineal gland visible in most (*n* = 28) paratypes, and in representative specimens of all paratype locations. Measurements are summarized in Table 1.

#### Molecules

*Sylvirana montosa* sp. nov. was recovered in the mtDNA Bayesian analysis to be the sister taxon to *S*. *annamitica* sp. nov. (Fig 1 and Fig 4), but from which it is distinct in morphospace (Fig 7). *Sylvirana montosa* was admixed in the nuDNA Bayesian analysis with *S*. *mortenseni*, *S*. *nigrovittata*, and *S*. *annamitica* sp. nov. (Fig 5), but clustered in the nuDNA phylogenetic network with *S*. *mortenseni* (Fig 6).

#### Distribution

This species ranges from southern Vietnam and eastern Cambodia through central Laos (Fig 11). *Sylvirana montosa* sp. nov. may be sympatric with *S*. *annamitica* sp. nov. in Nakai-Nam Theun National Protected Area, Laos.

#### Comparisons

*Sylvirana montosa* sp. nov. can be distinguished from *S*. *annamitica* sp. nov. by having males larger than 52 mm SVL and females larger than 54 mm SVL (the reverse condition in *S*. *annamitica* sp. nov.). *Sylvirana montosa* sp. nov. is distinguished from *S*. *lacrima* sp. nov. in having an irregular pattern of dark marbling on light background on posterior surface of thighs (indistinct pattern in *S*. *lacrima* sp. nov.), and in lacking an oblique teardrop-shaped or triangular mark slightly posteriorly to tympanum (present in *S*. *lacrima* sp. nov.). *Sylvirana montosa* sp. nov. can be distinguished from *S*. *malayana* sp. nov. in lacking a strong demarcation between dark (upper) and light (lower) parts on the flank (present in *S*. *malayana* sp. nov.). This species is distinguished from *S*. *mortenseni* by having significantly smaller males (SVL: 57.7 ± 6.2, *S*. *mortenseni* SVL 62.7 ± 8.7; t-stat = -3.44, df = 96, p < 0.001), although females are roughly subequal in size (59.1 ± 5.2 and 60.3 ± 6.2 for *S*. *montosa* sp. nov. and *S*. *mortenseni*, respectively, t-stat = 0.68, df = 27, p = 0.50). *Sylvirana montosa* sp. nov. differs from *S*. *nigrovittata* by males having a larger HDW:HDL (0.94 ± 0.03; *S*. *nigrovittata* 0.91 ± 0.03; t-stat = -5.08, df = 120, p < 0.001) and a larger HDW:SVL (0.37 ± 0.01; *S*. *nigrovittata*, 0.36 ± 0.01; t-stat = 5.90, df = 106, p < 0.001), and by females having a larger TMP:EYE (0.73 ± 0.08) than *S*. *nigrovittata* (0.67 ± 0.05; t-stat = -3.65, df = 52, p < 0.001) and a larger HDW:SVL (0.345 ± 0.012; *S*. *nigrovittata*, 0.338 ± 0.012; t-stat = -2.57, df = 71, p = 0.01). *Sylvirana montosa* sp. nov. differs from *S*. *roberti* sp. nov. in possessing a pineal gland (absent in *S*. *roberti* sp. nov.). Males and females were correctly assigned to this species 91% and 86% of the time, respectively, in DFA analyses.

***Sylvirana annamitica* sp. nov.**

*Zoobank ID*: urn:lsid:zoobank.org:act:2016CCC9-6AD2-4A83-BD06-4D2ED2909D6B

*Rana nigrovittata* Ziegler, 2002: 102 [65]; Gawor, Hendrix, Vences, Böhme & Ziegler, 2009: 1 (part) [30].

*Rana (Sylvriana) nigrovittata* Grosjean, 2005: 61 [66].

*Sylvirana nigrovittata* Frost, Grant, Faivovich, Bain, Haas, Haddad, de Sá, Channing, Wilkinson, Donnellan, Raxworthy, Campbell, Blotto, Moler, Drewes, Nussbaum, Lynch, Green, and Wheeler, 2006: 319 [31].

*Sylvirana nigrovittata* sp. 2 Oliver, Prendini, Kraus & Raxworthy, 2015: 191 [29].

#### Holotype

FMNH 256535, adult male, Laos, Khammouan Province, Nakai District, Nakai-Nam Theun National Protected Area (formerly Nakai-Nam Theun National Biodiversity Conservation Area), 17.9667 N, 105.5667 E, 700 m elev., coll. 6 November 1998 by Bryan L. Stuart.

#### Paratypes

FMNH 256538 (adult male), FMNH 256539 (adult female), same data as holotype except coll. 7 November 1998. FMNH 256533 (adult male), same data as holotype except 17.9500 N 105.5667 E, coll. 9 November 1998. FMNH 256540 (adult male), same data as holotype except 17.9500 N 105.5667 E, coll. 12 November 1998.

AMNH A-161461 (adult female), Vietnam, Bac Kan Province, Ba Be District, Ba Be Lake National Park, 22.4003°N 105.6317°E, coll. 8 August 1997 by Darrel R. Frost and Christopher J. Raxworthy.

FMNH 255629 (adult male), Vietnam, Nghe An Province, Con Cuong District, Pu Mat Nature Reserve, 18.9333 N 104.7500 E, 300 m elev., coll. 5 September 1998 by Bryan L. Stuart.

AMNH A-161290 (adult male), AMNH A-161280 (adult female), Vietnam, Ha Tinh Province, Rao Cai, Ke Go Natural Reserve, coll. 11 April 1998 by David A. Kizirian and Truong Q. Nguyen.

AMNH A-169308 (adult male), Vietnam, Thua Thien-Hue Province, A Luoi District, Tram Tra Ve (forestry station of Huong Giang State Forestry Enterprise), 16.2606 N 107.4439 E, coll. 16 August 2005 by Raoul H. Bain and Truong Q. Nguyen.

NCSM 79174 (adult male), Vietnam, Quang Nam Province, Nam Giang District, Song Thanh Nature Reserve, near Khe Giua, 15.6591 N 107.6015 E, 335 m elev., coll. 25 May 2010 by David A. Kizirian and Truong Q. Nguyen.

ROM 27786 (adult female), Vietnam, Vinh Phu Province, Tam Dao District, Tam Dao National Park, 21.4542 N, 105.6414 E, approximately 800 m elev., coll. 4 June 1995 by Robert W. Murphy.

#### Referred material

Additional specimens are referred to the new species in S2 Table.

#### Etymology

The specific epithet refers to the Annamite Mountains (as Xai Phou Luang in Lao or Day Truong Son in Vietnamese) along the border of Laos and Vietnam that coincide with most of the geographic range of the new species.

#### Diagnosis

A species of *Sylvirana* having the combination of all digit tips expanded with circummarginal grooves; skin finely granular above, smooth below; males with SVL 40.2–52.0 mm, females with SVL 49.5–53.7 mm; males with enlarged humeral glands and small, thin nuptial pads; pineal gland visible; no oblique, triangular or teardrop-shaped marking slightly posterior to the tympanum; and flank with narrow dark stripe below dorsolateral fold extending from eye to groin, but without strong demarcation between dark (upper) and light (lower) parts of flank.

#### Description of holotype

**Fig 15:**
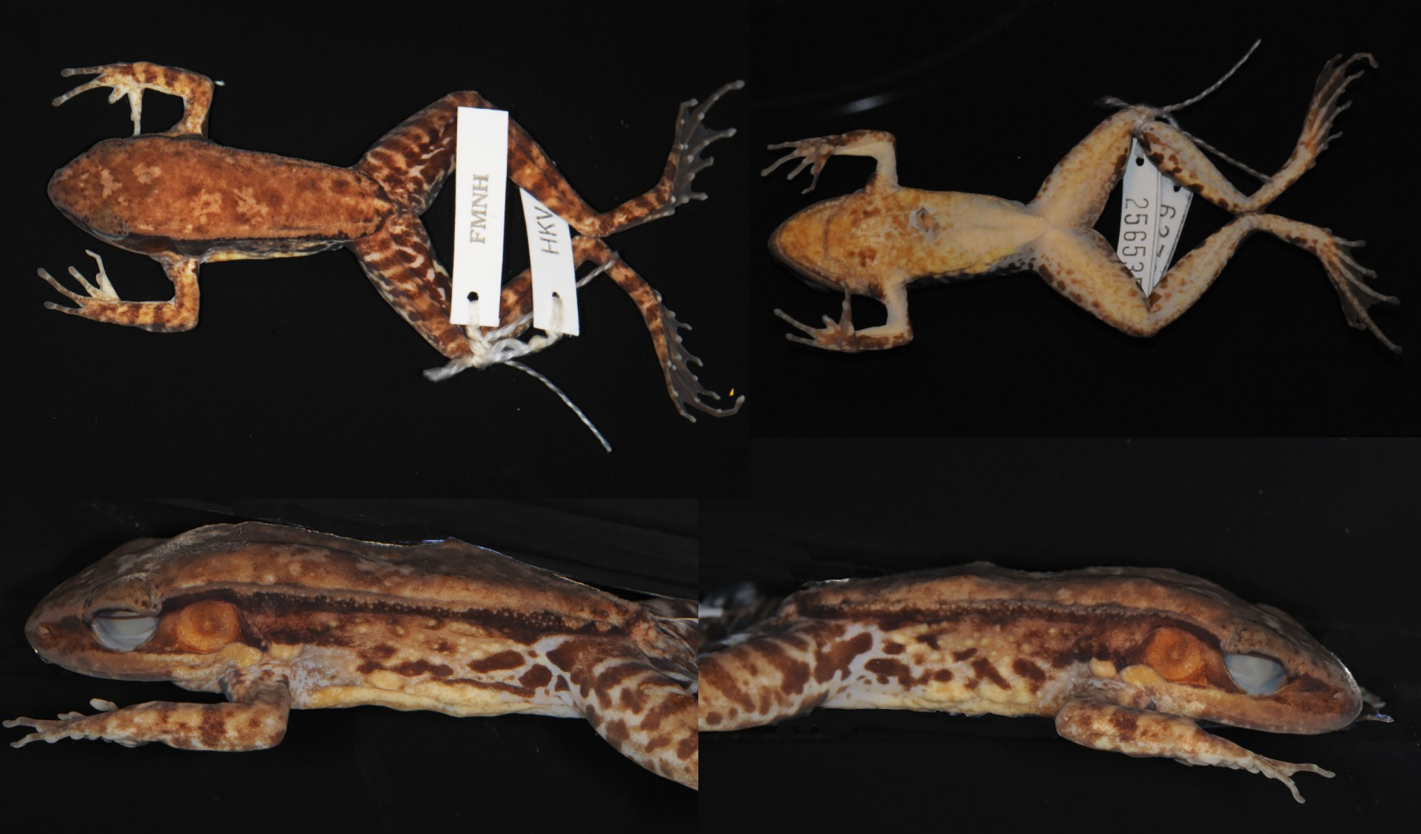
Holotype of *Sylvirana annamitica* sp. nov., male, FMNH 256535 from Nakai-Nam Theun National Protected Area, Khammouan Province, Laos.

Adult male (Fig 15), 47.3 mm SVL. Habitus moderately slender. Head length greater than head width. Snout obtusely pointed in dorsal view, projecting beyond lower jaw and rounded in profile. Nostril lateral, closer to tip of snout than to eye; internarial distance greater than interorbital distance. Canthus rostralis distinct, lores oblique and concave. Eye diameter 97% snout length; interorbital distance slightly greater than upper eyelid width (4.03 mm). Pineal body faintly visible. Round tympanum 75% eye diameter, slightly depressed relative to skin of temporal region, tympanic rim elevated relative to tympanum. Vomerine teeth obliquely angled, equidistant from choanae as from each other. Tongue cordiform, notched posteriorly. Small slit-like vocal sac openings near corner of jaw. No gular pouch.

Forelimbs slightly robust. Tips of fingers expanded into small discs with circummarginal grooves, width of Finger III disc (0.97 mm) only slightly (about 13%) greater than width of phalanx (0.86 mm), and about 22% diameter of tympanum. Relative finger lengths IV < II < I < III. Webbing absent. Subarticular tubercles conspicuous and rounded; formula: 1, 1, 2, 2. One supernumerary tubercle at the base of each finger; two oval palmar tubercles; one oval thenar tubercle. Humeral glands enlarged. Nuptial pads thin and small, but visible under dissecting microscope.

Hindlimbs long. Tips of toes expanded into discs with circummarginal grooves, width of Toe IV disc (1.26 mm) 26% that of tympanum. Webbing on Toe I to base of disc, on preaxial side of Toe II to subarticular tubercle, on postaxial side of Toe II to base of disc, on preaxial side of Toe III to distal subarticular tubercle, on postaxial side of Toe III to base of disc, on preaxial side of Toe IV to penultimate subarticular tubercle, on postaxial side of Toe IV to distal subarticular tubercle, and on Toe V to base of disc. Subarticular tubercles conspicuous, surfaces rounded; formula: 1, 1, 2, 3, 2. Inner metatarsal tubercle elongate; outer metatarsal tubercle round.

Skin finely granular dorsally, smooth ventrally, slightly glandular on flanks. Supratympanic fold extending from dorsolateral fold to rictal gland, along posterior margin of tympanum. Two rictal glands: anterior elongate and continuous with upper lip, posterior gland oval. Distinct dorsolateral folds. Two low postorbital swellings on top of head.

Measurements (mm): SVL 47.3, HDL 18.3, HDW 15.5, SNT 6.6, EYE 6.4, IOD 4.4, IND 4.8, TMP 4.8, SHK 24.4, TGH 19.0, HND 11.4, FTL 24.6.

#### Coloration of holotype in preservative

Dorsum medium brown, with some dark brown mottling and light-colored spots. Dorsal surfaces of hindlimbs and forelimbs with dark crossbars. Posterior surface of thighs with irregular pattern of dark marbling on light background. Dorsolateral fold dark brown. Flanks pale with dark brown spots. Lip grey-brown anteriorly and beige posteriorly. Humeral glands dark brown. Dark mottling on throat, less mottling on chest, belly, and underside of thighs. Webbing and ventral surfaces of feet brown; ventral surfaces of hands dark brown.

#### Coloration of referred specimen AMNH A-181996 in life (based on photograph by David Kizirian)

Adult male (Fig 10E), SVL 43.3, Phuoc Son District, Song Thanh Nature Reserve, Quang Nam Province, Vietnam. Dorsum reddish brown with paler spots and some darker spots. Dark stripe extending along ventral margin of dorsolateral fold from snout through eye and tympanum to groin, reduced to a narrow stripe posterior to axilla. Flanks yellowish-brown with dark brown spots. Dorsal surfaces of forelimbs beige with irregular dark markings. Dorsal surfaces of hindlimbs darker beige with dark brown cross bars. Lip and rictal glands white. Upper one-quarter of iris golden, lower part bronze.

#### Variation

Females lack robust forearms and humeral glands (but often have a dark brown spot where the humeral gland appears on males). Females (SVL 51.2 ± 1.8, n = 5) are larger than males (45.0 ± 2.9, n = 16), but we were unable to test this difference statistically due to small sample size of adult females. Measurements are summarized in Table 1.

Dorsal coloration varies from medium-to-dark brown, with variable dark mottling or spots. Lips grey, brown, or cream, or some combination of darker color anteriorly fading to cream posteriorly. Coloration on flanks usually with thin dark stripe just ventral to dorsolateral fold, fading to light ventral coloration with distinct spots, but a few individuals have very dark flanks with dark spots. Ventral coloration ranges from immaculate creamy-white to very darkly mottled. A few specimens have two small faint stripes just lateral to the midline at the base of the throat. Posterior rictal gland may be ovoid or round.

#### Molecules

*Sylvirana annamitica* sp. nov. was recovered in the mtDNA Bayesian analysis to be the sister taxon to *S*. *montosa* sp. nov. (Fig 1 and Fig 4), from which it is distinct in morphospace (Fig 7). *Sylvirana annamitica* was admixed in the nuDNA Bayesian analysis with *S*. *mortenseni*, *S*. *nigrovittata*, and *S*. *montosa* sp. nov. (Fig 5), but clustered in the nuDNA phylogenetic network near to *S*. *nigrovittata* (Fig 6).

#### Distribution

This species ranges from northern Vietnam southward through central Laos and central Vietnam, with most of its distribution coinciding with the Annamite (= Xai Phou Luang or Day Truong Son) Mountains (Fig 11).

#### Comparisons

*Sylvirana annamitica* sp. nov. differs from *S*. *lacrima* sp. nov. in having an irregular pattern of dark marbling on light background on posterior surface of thighs (indistinct pattern in *S*. *lacrima* sp. nov.), and in lacking an oblique teardrop-shaped or triangular marking posterior to tympanum (present in *S*. *lacrima* sp. nov.). *Sylvirana annamitica* sp. nov. can be distinguished from *S*. *malayana* sp. nov. by lacking a strong demarcation between dark (upper) and pale (lower) parts of flank (present in *S*. *malayana* sp. nov.). *Sylvirana annamitica* sp. nov. differs from both *S*. *montosa* sp. nov. and *S*. *mortenseni* by having smaller SVL for both males and females (Table 1). Males of this species strongly overlap with *S*. *nigrovittata* in morphospace, but can be distinguished by the significantly shorter TGH:SVL (0.47 ± 0.05; *S*. *nigrovittata*: 0.51 ± 0.04; t-stat = -3.16, df = 19, two-tailed p < 0.01). *Sylvirana annamitica* sp. nov. can be distinguished from *S*. *roberti* sp. nov. by the presence of a pineal gland (absent in *S*. *roberti* sp. nov.). Males were correctly assigned to this species 69% of the time in DFA, and females (n = 5) were correctly assigned to species 100% of the time.

#### Remarks

The type locality of *Hylarana (Sylvirana) hekouensis* Fei, Ye and Jiang, 2008 lies only approximately 170 air-km away from our northernmost sample of *S*. *annamitica* sp. nov. at Bac Kan Province, Vietnam. However, our morphological evidence suggests that the new species is not synonymous with *H*. (*S*.) *hekouensis*, but rather that *H*. (*S*.) *hekouensis* is more likely to be synonymous with *S*. *nigrovittata* (see that species account above).

Paratype female AMNH A-161280 was also included in the molecular analyses of Frost et al. [31] and Oliver et al. [29].

***Sylvirana malayana* sp. nov.**

*Zoobank ID*: urn:lsid:zoobank.org:act:7216437F-3716-41FE-96C8-9116CAF89209

*Rana nigrovittata* Berry, 1975: 81 [67].

*Hylarana (Sylvirana) nigrovittata* Fei, Ye and Xie, 2008: 201–204 [57]; Fei, Ye and Jiang, 2008: 199–201 [57].

#### Holotype

FMNH 268770, adult male, Thailand, Surat Thani Province, Khao Sok National Park, 8.9147 N, 98.5278 E, coll. 29 December 2003 by Tanya Chan-ard.

#### Paratypes

FMNH 268771–72 (two adult males), same data as holotype.

FMNH 268388, FMNH 268392 (two adult males), FMNH 268387 (adult female), Thailand, Surat Thani Province, Kaeng Krung National Park, coll. 29 November–1 December 2004 by Jennifer A. Sheridan.

FMNH 268763–64 (two adult females), Thailand, Krabi Province, Khao Phanom Bencha National Park, coll. on 17 December 2003 by Tanya Chan-ard.

FMNH 268384 (adult male), Thailand, Ranong Province, Ngao Falls National Park, coll. 26 November 2004 by Jennifer A. Sheridan.

LSUHC 5855–56 (two adult males), LSUHC 5854 (adult female), Malaysia, Perak, PITC logging camp, coll. by L. Lee Grismer.

CAS 247472 (adult male), Myanmar (Burma), Tanintharyi State, Khamaukgyi Township, 10.3720 N, 98.6086 E, 28 m elev., coll. 12 January 2010 by M. Hlaing, S.L. Oo, A.H. Aung, and K.S. Lwin. CAS 247856 (adult male), Myanmar (Burma), Tanintharyi State, Khamaukgyi Township, 10.3698 N, 98.6313 E, 53 m elev., coll. 26 January 2010 by M. Hlaing, S.L. Oo, A.H. Aung, and K.S. Lwin. CAS 247866 (adult male), Myanmar (Burma), Tanintharyi State, Khamaukgyi Township, 10.3794 N, 98.6092 E, 42 m elev., coll. 28 January 2010 by S.L. Oo, Z.H. Aung, and K.S. Lwin.

#### Referred material

Additional specimens are referred to the new species in S2 Table.

#### Etymology

The specific epithet refers to the new species being the only member of the *S*. *nigrovittata* species complex that is currently known to occur in Peninsular Malaysia.

#### Diagnosis

A species of *Sylvirana* having the combination of all digit tips expanded with circummarginal grooves; skin finely granular above, smooth below; males with SVL 42.2–48.8 mm, females with SVL 47.2–56.8 mm; males with enlarged humeral glands and narrow nuptial pads; pineal gland visible; no oblique, triangular or teardrop-shaped marking slightly posterior to the tympanum; and dark stripe extending from snout to groin, with strong demarcation between dark (upper) and light (lower) parts of flank.

#### Description of holotype

**Fig 16:**
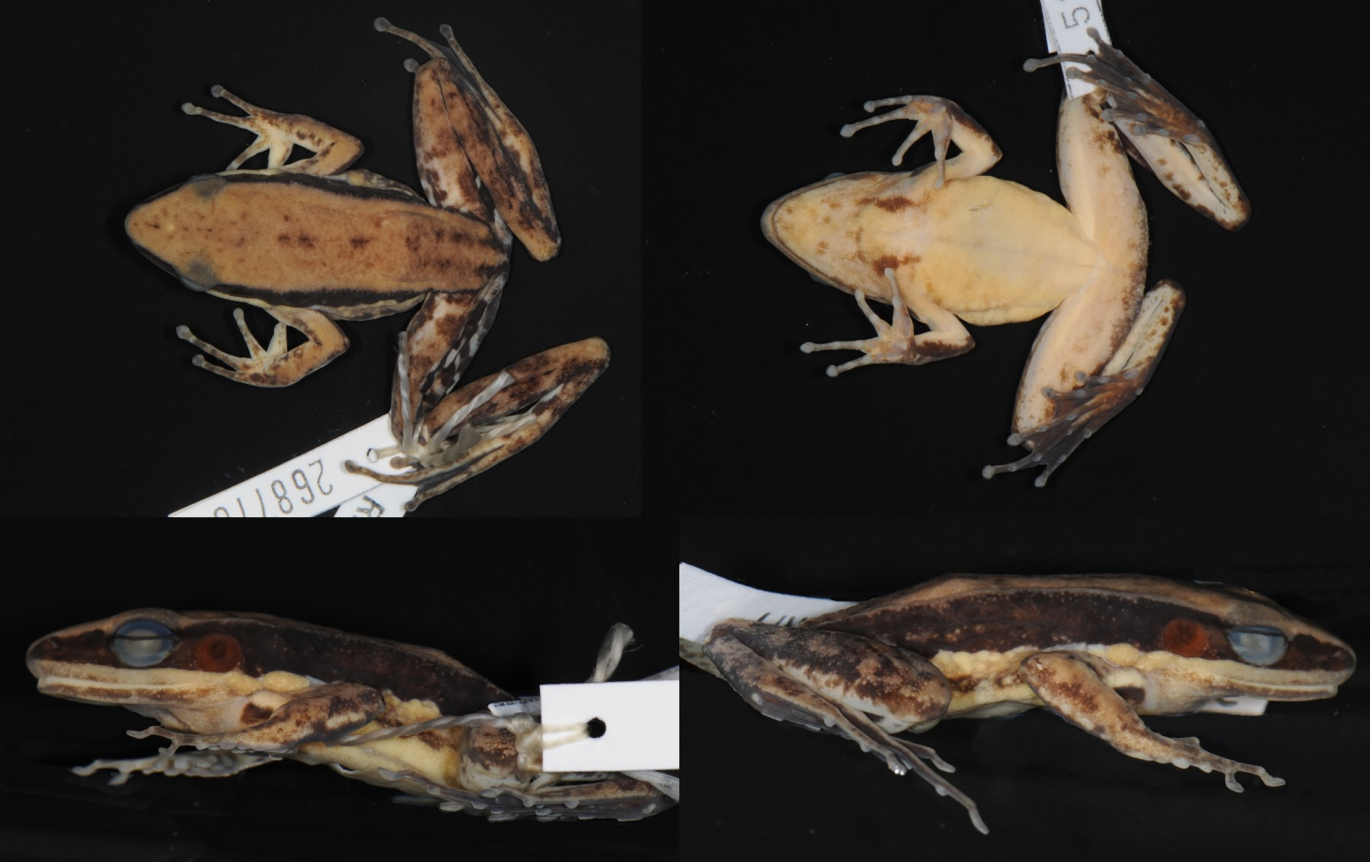
Holotype of *Sylvirana malayana* sp. nov., male, FMNH 268770 from Khao Sok National Park, Surat Thani Province, Thailand.

Adult male (Fig 16), 42 mm SVL. Habitus slender; head length greater than head width. Snout obtusely pointed in dorsal view, projecting beyond lower jaw, round in profile. Nostril lateral, closer to tip of snout than to eye. Internarial distance greater than interorbital distance. Canthus rostralis distinct, lores oblique and concave. Eye diameter 85% snout length; interorbital distance slightly greater than upper eyelid width (3.5 mm). Pineal body visible. Distinct, round tympanum, 69% the eye diameter, slightly depressed relative to the skin of the temporal region; tympanic rim elevated relative to tympanum. Vomerine teeth obliquely angled, equidistant from each other as from choanae. Tongue cordiform, notched posteriorly. Small slit-like vocal sac openings near corner of jaw. No gular pouch.

Forelimbs moderately robust. Tips of all four fingers expanded into discs with circummarginal grooves. Width of Finger III disc (1.17 mm) approximately 60% greater than width of phalanx (0.73 mm), and 31% the diameter of tympanum. Relative finger lengths IV < II < I < III. One supernumerary tubercle at the base of each finger. Subarticular tubercles conspicuous, surfaces rounded; formula: 1, 1, 2, 2. Two oval palmar tubercles, one oval thenar tubercle. Webbing absent. Narrow nuptial pads present.

Hindlimbs long, moderately slender. Tips of toes expanded into discs, with circummarginal grooves. Width of Toe IV disc (1.5 mm) 40% that of tympanum. Webbing on Toe I to base of disc, on preaxial side of Toe II to just distal of subarticular tubercle, on postaxial side of Toe II to base of disc, on preaxial side of Toe III to distal subarticular tubercle, on postaxial side of Toe III to base of disc, on Toe IV to distal subarticular tubercles, on Toe V to base of disc. Subarticular tubercles conspicuous, surfaces rounded; formula: 1, 1, 2, 3, 2. Inner metatarsal tubercle elongate; outer metatarsal tubercle round.

Skin finely granular dorsally, smooth ventrally. Supratympanic fold extending from dorsolateral fold to rictal gland, along posterior margin of tympanum. Two rictal glands, anterior gland elongate and continuous with upper lip, posterior round. Dorsolateral fold present. Skin on flanks with enlarged oval and round small to medium glands. No visible postorbital swellings on top of head.

Measurements (mm): SVL 42.7, HDL 16.6, HDW 13.3, SNT 6.3, EYE 5.4, IOD 3.9, IND 4.2, TMP 3.7, SHK 24.0, TGH 22.4, HND 11.6, FTL 22.1.

#### Coloration of holotype in preservative

Dorsum medium brown with several small to medium dark spots. Distinct, broad dark band extending along ventral margin of dorsolateral fold from snout to groin, forming dark dorsal half of flank. Ventral half of flank cream with dark brown markings, with strong demarcation between dorsal and ventral halves on flank. Upper lip creamy-grey anteriorly, lighter posteriorly. Humeral glands with dark brown spots. Dorsal surfaces of forelimbs and hindlimbs with dark crossbands. Posterior surface of thighs with irregular pattern of dark marbling on light background. Ventral surfaces of hindlimbs cream, with some dark brown mottling and spotting laterally.

Throat cream with some dark brown mottling. Two brown stripes just lateral to the midline at base of the throat. Venter cream with minimal dark brown mottling laterally. Toe webbing brown. Rictal gland cream colored. Inguinal region and armpit distinctly lighter in coloration than adjacent areas. Dorsal surfaces of thighs with two pale spots near to inguinal region.

#### Coloration in life (based on photograph by L. Lee Grismer)

Unvouchered individual (Fig 10F), Temenggor, Malaysia. Dorsum medium brown with very small dark spots. Lips white. Distinct, broad dark band extending along ventral margin of dorsolateral fold from snout to groin. Flank dark on dorsal half, creamy white on ventral half, with strong demarcation between the two colors. Few dark brown spots on lower half of flank. Dorsal surfaces of forelimbs medium brown with dark brown spots. Dorsal surfaces of hindlimbs with dark brown cross bars. Inguinal region with yellow wash.

#### Variation

Females lack robust forearms and humeral glands (but often have a dark brown spot where humeral glands appear in males). Although males and females overlap in size, females are larger on average (SVL 52.7 ± 3.2) than males (SVL 44.7 ± 2.0; t-stat = -6.25, df = 8, p < 0.001; Table 1). Crossbars on dorsal surfaces of hindlimbs indistinct in five individuals (CAS 247865–66, 247209, 247472, and LSUHC 05855). Dorsal surfaces of forelimb paler than dorsum in some individuals. Dark spots on the pale (ventral) half of the flank vary greatly in size. Lips vary from creamy white to brown. Webbing on postaxial side of Toe IV reaches distal subarticular tubercle in some specimens. Ventral surface varies from immaculate cream to having dark brown spots. Pair of dark stripes at base of throat indistinct in four individuals (FMNH 268385, CAS 247866, 247472, LSUHC 05854) and absent in one individual (LSUHC 0855). Ventral surfaces of hands range from mostly cream to completely dark brown. Posterior rictal gland round or oval. Measurements are summarized in Table 1.

#### Molecules

*Sylvirana malayana* sp. nov. was recovered in the mtDNA Bayesian analysis to be the sister taxon to a clade containing *S*. *nigrovittata*, *S*. *roberti* sp. nov., *S*. *annamitica* sp. nov., and *S*. *montosa* sp. nov. (Fig 1). *Sylvirana malayana* sp. nov. was also recovered as a distinct lineage in the nuDNA Bayesian analysis and nuDNA phylogenetic network (Figs 5 and 6).

#### Distribution and natural history

This species occurs from southern peninsular Myanmar and Thailand through the Malay Peninsula (Fig 11). Berry [67] noted that it occurs (as “*Rana nigrovittata*”) in the Malaysian states of Perlis, Kedah, Perak, and Selangor, all of which are along the west coast of Peninsular Malaysia, with the southernmost reference being Batu Caves, Selangor, just north of Kuala Lumpur.

#### Comparisons

The strong demarcation of light and dark coloring on the flank distinguishes *S*. *malayana* sp. nov. from all other species in the *S*. *nigrovittata* complex (Fig 10). Additionally, females of this species have the smallest HDW:HDL of any species (0.83 ± 0.02; ANOVA F(4,111) = 15.49; p < 0.001), but the largest SNT:SVL (0.15 ±0.01; ANOVA F(4,111) = 6.16; p < 0.001), and a significantly smaller HDW:SVL than females of other species that were tested (0.32 ± 0.004; ANOVA F(4,111) = 11.38, p < 0.001), including *S*. *lacrima* sp. nov. (0.33 ± 0.008; t-stat = -3.94, df = 7, p < 0.01). DFA revealed that males of this species strongly overlap in morphospace with *S*. *roberti* sp. nov., but males of this species have larger TMP:EYE ratio (0.74 ± 0.06; t-stat = -2.35, df = 31, two-tailed p < 0.02), and a smaller HDW:SVL (0.33 ± 0.01; t-stat = 4.43, df = 28, two-tailed p < 0.001) than *S*. *roberti* sp. nov. (0.70 ± 0.03 and 0.34 ± 0.01, respectively). DFA correctly assigned males to this species 77% of the time, and females to this species 100% of the time.

***Sylvirana roberti* sp. nov.**

*Zoobank ID*: urn:lsid:zoobank.org:act:BAB6D05C-2858-488B-AC3A-F1851C694D36

#### Holotype

CAS 243913, adult male, Myanmar (Burma), Tanintharyi State, Dewei District, Yephyu Township, Tanintharyi Nature Reserve, 14.7475 N, 98.2213 E, 73 m elev., coll. 23 March 2009 by S.L. Oo, K.S. Lwin, and Z.H. Aung.

#### Paratypes

CAS 229665 (adult male), CAS 229678 (adult female), Myanmar (Burma), Tanintharyi State, Dewei District, Thaye Chaung Township, 13.8613 N, 98.2883 E, 52 m elev., coll. 18 January 2003 by H. Win, H. Tun, K.S. Lwin, A.K. Shein, and Z.M. Naing. CAS 229796, adult male, Myanmar (Burma), Tanintharyi State, Dewei District, Thaye Chaung Township, near border of Nwa La Bo Reserve Forest, 13.8453 N, 98.4585 E, 61 m elev., coll. 28 January 2003 by H. Win, H. Tun, K.S. Lwin, A.K. Shein, and Z.M. Naing.

#### Referred material

Additional specimens are referred to the new species in S2 Table.

#### Etymology

The specific epithet is a patronym honoring our mentor and friend, Datuk Dr. Robert F. Inger, Curator Emeritus of Amphibians & Reptiles, Field Museum of Natural History, in recognition of his immeasurable contributions to the biology and conservation of Southeast Asian frogs.

#### Diagnosis

A species of *Sylvirana* having the combination of all digit tips expanded with circummarginal grooves; skin finely granular above, smooth below; males with SVL 41.6–45.4, female with SVL 49.2; males with enlarged humeral glands; pineal gland not visible; dark stripe extending from snout to groin, but without strong demarcation between dark (upper) and light (lower) parts of flank; and no oblique, triangular or teardrop-shaped marking slightly posterior to the tympanum.

#### Description of holotype

**Fig 17:**
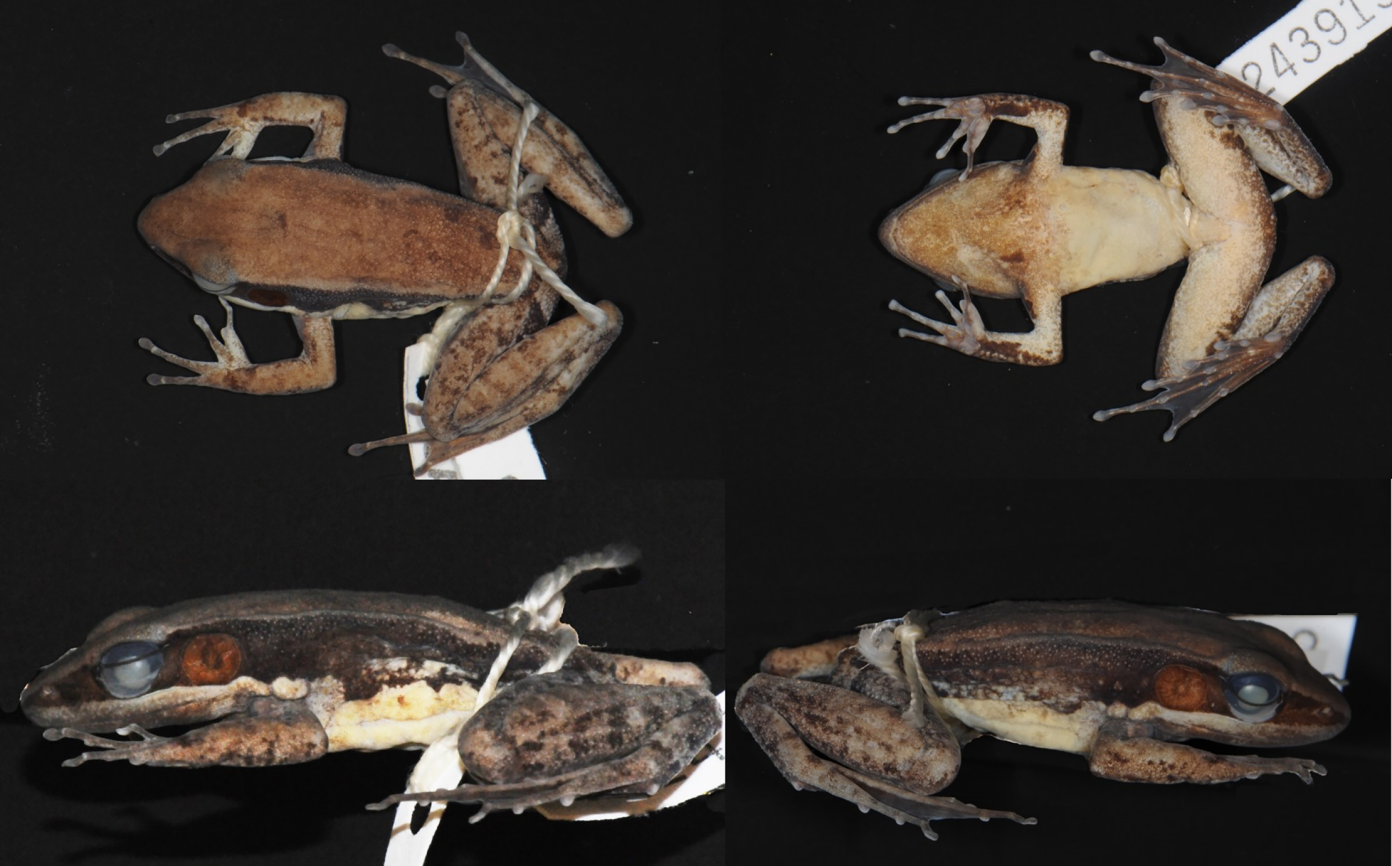
Holotype of *Sylvirana roberti* sp. nov., male, CAS 243913 from Tanintharyi Nature Reserve, Tanintharyi State, Myanmar (Burma).

Adult male (Fig 17), 43.6 mm SVL. Habitus moderately slender. Head length greater than head width. Snout obtusely pointed in dorsal view, projecting beyond lower jaw, rounded in lateral view. Nostril lateral, much closer to tip of snout than to eye. Internarial greater than interorbital distance. Canthus rostralis distinct, lores oblique and concave. Eye diameter 84% snout length, interorbital distance greater than upper eyelid width (3.8 mm). Pineal body not visible. Round tympanum, 73% eye diameter, slightly depressed relative to skin of temporal region; tympanic rim elevated relative to tympanum. Vomerine teeth obliquely angled, equidistant from choanae as from each other. Tongue cordiform, notched posteriorly. Slit-like vocal sac opening near corner of jaw. No gular pouches present.

Forelimbs moderately robust. Tips of all four fingers expanded into discs with circummarginal grooves; width of finger III disc (1.10 mm) 55% greater than width of phalanx (0.71 mm), 26% that of tympanum. Relative finger lengths II < IV < I < III. Webbing absent. Subarticular tubercles conspicuous, surfaces rounded; formula: 1, 1, 2, 2. One supernumerary tubercle at the base of each finger. Two oval palmar tubercles, one oval thenar tubercle. Nuptial pad on underside of thumb to subarticular tubercle. Humeral gland enlarged.

Hindlimbs long and moderately muscular. Tips of toes expanded into discs with circummarginal grooves, width of Toe IV disc (1.16 mm) 27% that of tympanum. Webbing on Toe I to base of disc, on preaxial side of Toe II to subarticular tubercle, on postaxial side of Toe II to base of disc, on preaxial side of Toe III to distal subarticular tubercle, on postaxial side of Toe III to base of disc, on Toe IV to distal subarticular tubercle, and to base of disc on Toe V. Subarticular tubercles conspicuous, surfaces rounded; formula: 1, 1, 2, 3, 2. Inner metatarsal tubercle oval; outer metatarsal tubercle round.

Skin finely granular dorsally, smooth ventrally. Supratympanic fold extending from dorsolateral fold to rictal gland, along posterior margin of tympanum. Two rictal glands: anterior elongate and continuous with upper lip, posterior gland oval. Dorsolateral fold present. Skin on flanks with small to medium enlarged round and oval glands. Two low postorbital swellings on top of head.

Measurements (mm): SVL 43.6, HDL 17.1, HDW 14.8, SNT 7.0, EYE 5.8, IOD 4.0, IND 4.6, TMP 4.2, SHK 22.1, TGH 21.3, HND 10.7, FTL 21.8.

#### Coloration of holotype in preservative

Dorsum medium to dark brown. Dark stripe extending along ventral margin of dorsolateral fold from tip of snout to groin. Dorsal surfaces of forelimbs light to medium brown with some dark brown spots. Dorsal surfaces of hindlimbs with faint dark crossbars. Posterior surface of thighs with irregular pattern of dark marbling on light background. Webbing brown. Humeral glands dark brown. Chin mottled brown. Two small faint stripes just lateral to the midline at base of the throat. Chest mottled, belly creamy white. Ventral surfaces of thighs cream with heavy mottling and spotting laterally. Ventral surfaces of forelimbs cream with brown mottling and dark spots just proximal to hands.

#### Coloration of paratopotype in life (based on photograph by Jens Vindum)

CAS 229664 (not examined by us; Fig 10G), Thaye Chaung Township, Dewei District, Tanintharyi State, Myanmar (Burma). Dorsum medium brown. Dark stripe extending along ventral margin of dorsolateral fold from tip of snout to groin. Ventral half of flank mostly cream with dark spots that merge into dark stripe on upper flank. Upper one-third of iris golden brown, lower two-thirds metallic brown. Lip and rictal glands white. Chin mottled grey-brown. Humeral gland dark. Dorsal surfaces of hindlimbs brown with dark brown cross bars. Dorsal surfaces of forelimbs medium brown with few small dark spots.

#### Variation

Females lack robust forearms and humeral glands (but often have a dark brown spot where humeral glands appear in males). Female (SVL 49.2 mm) larger than males (SVL 43.6 ± 1.3 mm). Dorsum varies from beige to medium or dark brown with some darker spotting, although most individuals are nearly uniformly colored without spots. Lips usually creamy-white, but are dark in a few individuals. Coloration on flank is variable: dark coloration may begin at snout and continue as a thick band through the eye and tympanum to the groin; the lores may be pale with dark coloration beginning posterior to tympanum extending to the groin; or dark coloration may be restricted to the area between the axilla and dorsolateral fold. All individuals have at least some creamy white coloration on the ventral half of the flank, but the amount and pattern varies: in some individuals, the flank is mostly white with some dark brown spots, but in most individuals, the light coloration is covered by several dark brown spots that blend into the darker coloration of the upper half of the flank. Skin on flank may be smooth or distinctly glandular. Posterior rictal gland may be absent, or small and round. Ventral surfaces vary from nearly immaculate creamy-white to very heavily mottled. The pair of thin dark stripes at base of throat are absent, extremely faint, or obscured by chin mottling in some individuals. Nuptial pads are visible under the microscope in 10 of 12 males that also have some enlargement of humeral glands (the two males without nuptial pads, CAS 229665, CAS 243898, may be very young adults nearing maturity). Measurements are summarized in Table 1.

#### Molecules

*Sylvirana roberti* sp. nov. was recovered in the mtDNA Bayesian analysis to be the sister taxon to *S*. *nigrovittata* (Figs 1 and 2), but to be distantly related to *S*. *malayana* sp. nov. (Fig 1) with which it greatly overlaps in morphospace (Fig 7). *Sylvirana roberti* sp. nov. was also recovered as a distinct lineage in the nuDNA Bayesian analysis (Fig 5), but clustered in the nuDNA phylogenetic network near to *S*. *nigrovittata* (Fig 6).

#### Distribution and natural history

This species is currently known only from Tanintharyi State in southern Myanmar (Burma; Fig 11).

#### Comparisons

This species is distinguished from *S*. *annamitica* sp. nov., *S*. *lacrima* sp. nov., *S*. *malayana* sp. nov., *S*. *montosa* sp. nov., *S*. *mortenseni*, and *S*. *nigrovittata* in lacking a pineal gland. Additionally, males have a larger SNT:SVL (0.156 ± 0.005) than others in this group (0.145–0.151 ± 0.006–0.007; ANOVA(6,256) F = 5.58, p < 0.001), including *S*. *malayana* sp. nov. (0.150 ± 0.007; t-stat = 2.88, df = 30, two-tailed p < 0.01) with which it strongly overlaps in morphospace. Further, this species has a larger HDW:SVL (0.34 ± 0.01) than *S*. *malayana* sp. nov. (0.33 ± 0.01; t-stat = 4.43, df = 28, two-tailed p < 0.001). DFA showed that males were correctly assigned to this species 83% of the time. Only a single female was available, and so was not included in DFA.

***Sylvirana lacrima* sp. nov.**

*Zoobank ID*: urn:lsid:zoobank.org:act:18BD98FC-F20C-4708-8DBD-89CE2878D9FB

*Sylvirana nigrovittata* sp. 1 Oliver, Prendini, Kraus, and Raxworthy, 2015: 191 [29].

#### Holotype

CAS 234925, adult male, Myanmar (Burma), Chin State, Mindat District, Mindat Township, Hteen Chaung village, 21.3868 N, 94.0567 E, 443 m elev., coll. 10 December 2005 by A.K. Shein and T. Nyo.

#### Paratypes

CAS 234990 (adult male), Myanmar (Burma), Chin State, Mindat District, Mindat Township, Lone Zone village, 21.7807 N, 93.7482 E, 701 m elev., coll. 13 March 2006 by A.K. Shein, T. Nyo, and L. Shein. CAS 235166 (adult male), CAS 235166 (adult female), Myanmar (Burma), Chin State, Mindat District, Mindat Township, Aone village, 21.2715 N 93.7523 E, 850 m elev., coll. 25 April 2006 by A.K. Shein and L. Shein.

CAS 231229 (immature female), Myanmar (Burma), Mandalay State, Nyaung-U District, Kyauk Pa Daung Township, Popa Mountain Park, 20.9046 N, 95.2360 E, 819 m elev., coll. 21 September 2002 by T. Thin, A. K. Shein, and H. Tun.

#### Referred material

Additional specimens are referred to the new species in S2 Table.

#### Etymology

The specific epithet *lacrima* (L.) is a noun in apposition meaning teardrop, in reference to the marking behind the tympanum of the new species.

#### Diagnosis

A species of *Sylvirana* having the combination of all digit tips expanded with circummarginal grooves; skin finely granular above, smooth below; males with SVL 38.5–45.5 mm, females with SVL 46.7–66.8 mm; males with weakly visible humeral glands, enlarged forelimbs, and well-developed nuptial pads; dorsum medium to dark brown, thin dark stripe just ventral to dorsolateral fold extending from snout to groin, but without strong demarcation between dark (upper) and light (lower) parts of flank; an oblique, triangular or teardrop-shaped marking slightly posterior to the tympanum; indistinct pattern on posterior surface of thighs; and the postaxial side of Toe IV webbed to base of disc.

#### Description of holotype

**Fig 18:**
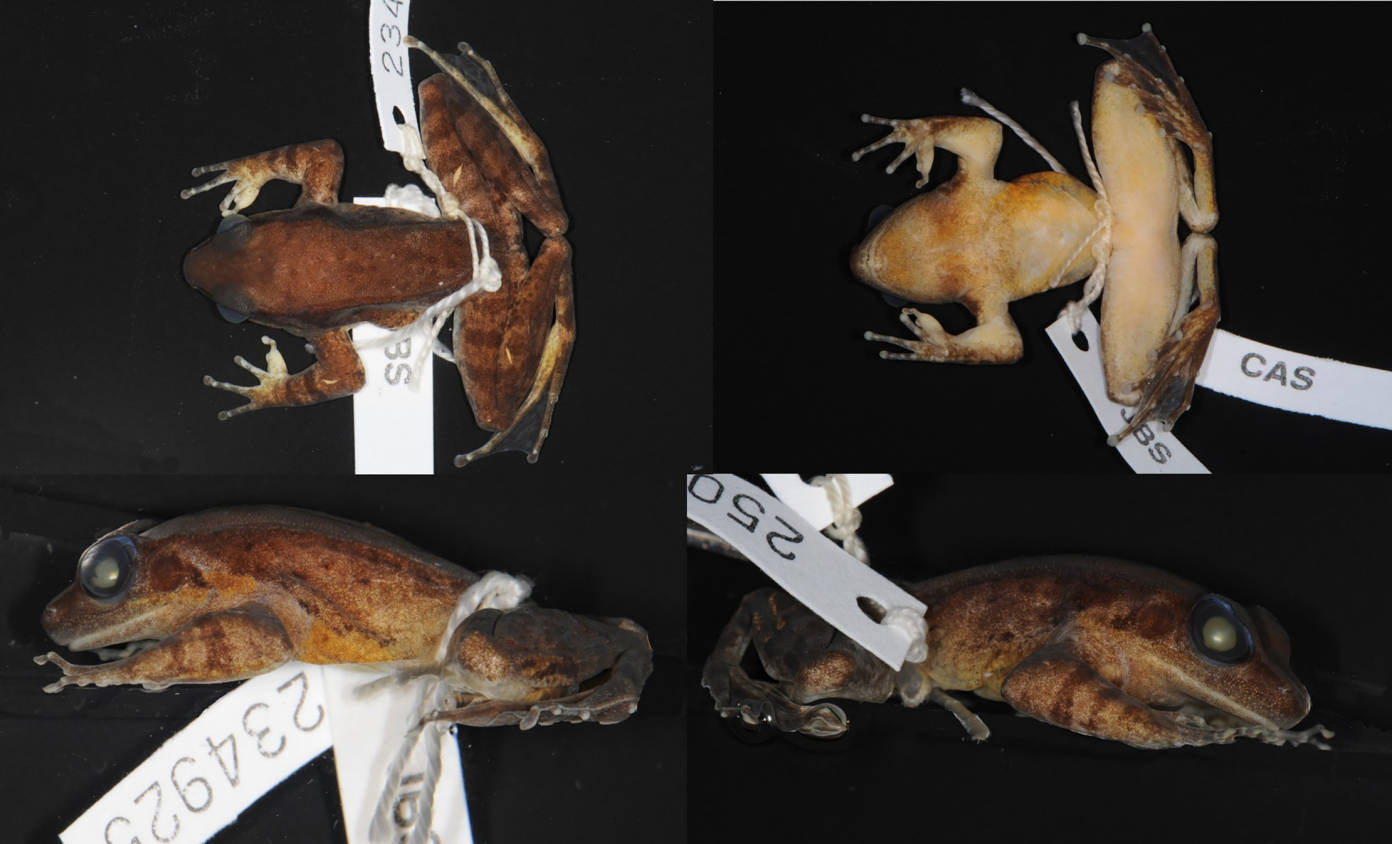
Holotype of *Sylvirana lacrima* sp. nov., male, CAS 234925 from Mindat Township, Chin State, Myanmar (Burma).

Adult male (Fig 18), 41.3 mm SVL. Habitus moderately slender. Head length subequal to head width. Snout obtusely pointed in dorsal view, projecting beyond lower jaw and rounded in profile. Nostril lateral, nearer to tip of snout than to eye. Internarial distance greater than interorbital distance. Canthus rostralis distinct; lores oblique and slightly concave. Eye diameter 94% of snout length. Interorbital distance greater than upper eyelid width (2.8 mm). Pineal body not visible. Tympanum distinct, round, 59% of eye diameter, slightly depressed relative to skin of temporal region, tympanic rim elevated relative to tympanum. Vomerine teeth obliquely angled, about twice as far from choanae as from each other. Tongue cordiform, notched posteriorly. Small slit-like vocal sac openings near corner of jaw. No gular pouch.

Forelimbs very robust. Tips of all four fingers slightly expanded into discs, with circummarginal grooves. Finger III disc width (0.80) approximately 14% larger than phalanx diameter (0.70), and 25% of tympanum diameter. Relative finger lengths IV < II < I < III. Webbing absent. Subarticular tubercles conspicuous and rounded; formula: 1, 1, 2, 2. One supernumerary tubercle at the base of each finger. Two oval palmar tubercles; one oval thenar tubercle. Well-developed nuptial pad on Finger I, extending past level of subarticular tubercle. Humeral glands weakly visible.

Hindlimbs long and moderately muscular. Toes slender. Tips of toes expanded into discs, with circummarginal grooves, width of Toe IV disc (1.16 mm) 36% that of tympanum. Web on Toe I to base of disc, on preaxial side of Toe II to subarticular tubercle, on postaxial side of Toe II to base of disc, on preaxial side of Toe III to subarticular tubercle, on postaxial side of Toe III to base of disc, on preaxial side of Toe IV to distal subarticular tubercle, on postaxial side of Toe IV to base of disc, on Toe V to base of disc. Subarticular tubercles conspicuous and rounded; formula: 1, 1, 2, 3, 2. Inner metatarsal tubercle oval, outer metatarsal tubercle round. Distinct spinules on outer margin of Toe V.

Skin finely granular dorsally, smooth ventrally. Supratympanic fold absent. One rictal gland continuous with upper lip. Distinct dorsolateral fold. Flanks smooth, lacking obvious glands. No visible postorbital swellings on top of head.

Measurements (mm): SVL 41.26, HDL 14.15, HDW 13.18, SNT 5.86, EYE 5.53, IOD 3.45, IND 3.63, TMP 3.24, SHK 24.26, TGH 21.82, HND 9.7, FTL 21.79

#### Coloration of holotype in preservative

Dorsum dark brown. Canthus, lores, and lip medium-dark brown. Oblique teardrop-shaped dark marking slightly posterior to the tympanum. Ventral portion of flank darker in coloration than lower portion, but color shift is gradual. Dorsal surfaces of forelimbs and hindlimbs medium brown with dark brown cross bars. Webbing brown. Humeral glands dark brown. Chin mottled brown, fading to creamy beige posteriorly on belly and underside of thighs. Indistinct pattern on posterior surface of thighs.

#### Coloration of paratype CAS 231229 in life (based on photograph by Jens Vindum)

Immature female, SVL 53.4 mm (Fig 10H). Dorsum medium brown with some spotting. Distinct dark stripe from tip of snout through eye and tympanum extending to groin. Lips white. Tympanum very dark brown. Flank dark on top half, creamy white on bottom half. Flanks with small and medium-sized round and oval glands, and dark brown spots. Dorsal surfaces of forelimbs medium brown, forearms with dark cross bars. Dorsal surfaces of hind limbs dark brown with darker cross bars. Upper iris gold, lower iris bronze.

#### Variation

Females lack robust forearms and nuptial pads, and are considerably larger in size (SVL 55.7 ± 8.1 mm) than males (SVL 42.1 ± 2.0 mm). Spinules on outer margin of Toe V absent in many individuals. Webbing on postaxial side of Toe IV reaches only to distal subarticular tubercle in some individuals. Dorsum sometimes with dark brown spots. Dorsal and flank coloration may be very similar in preservative, or flanks may be darker. Dark stripe extending along ventral margin of dorsolateral fold varies from narrow to broad, those with the latter having the oblique marking posterior to tympanum obscured. Ventral half of flank varies from cream or grey-brown. Spots on flank vary greatly in size. Lip coloration varies from brown, grey-brown, or creamy white. Oblique dark marking slightly posterior to the tympanum triangular or less distinct in some individuals. Throat usually pale, but spotted or mottled, or entirely brown, in some individuals.

#### Molecules

*Sylvirana lacrima* sp. nov. was recovered in both mtDNA and nuDNA Bayesian analyses to be the basal member of *Sylvirana*, and to render the *S*. *nigrovittata* complex non-monophyletic with respect to *S*. *cubitalis* and *S*. *maosonensis* (Fig 1 and Fig 5). *Sylvirana lacrima* sp. nov. was also recovered as a distinct lineage in the nuDNA phylogenetic tree and network (Figs 5 and 6).

#### Distribution and natural history

This species is known from Chin and Mandalay States in western Myanmar (Fig 11).

#### Comparisons

This species is distinguished from all other members of this species group by having indistinct pattern on the posterior surface of thighs (irregular pattern of dark marbling on light background in other species), and by the presence of a small, oblique teardrop-shaped or triangular marking posterior to tympanum. No other members of this species group have webbing on Toe IV reaching the base of the disc, although this is variable in *S*. *lacrima* sp. nov. Males can also be distinguished from other members of this species group by the presence of large, well-developed nuptial pads, combined with a size of less than 46 mm SVL.

Overall, males of this species are smaller with proportionately smaller features (HDL, TMP, HND, e.g.) than other species in this group (Table 1). Male TMP:EYE ratio, for example, is smaller (0.63 ± 0.04) than other species (0.70–0.75 ± 0.03–0.06; ANOVA F (6,256) = 8.22, p < 0.001), as is HDL:SVL (0.36 ± 0.02; ANOVA F(6,256) = 21.64, p < 0.001), but SHK:SVL (0.58 ± 0.03) is larger than in other species (0.52–0.55 ± 0.01–0.03; ANOVA F(6,256) = 8.91, p < 0.001). Similarly, ANOVA shows that females have a smaller HDL:SVL (0.35 ± 0.01; other species 0.37 ± 0.01; ANOVA F(4, 111) = 6.20, p = 0.001) and TMP:EYE (0.60 ± 0.06; ANOVA F(4,111) = 8.57, p < 0.001) than other species tested (0.67–0.73 ± 0.05–0.08). Additionally, females have the largest SHK:SVL (0.59 ± 0.02; ANOVA F(4,111) = 11.99, p < 0.001), and TGH:SVL (0.55 ± 0.03; ANOVA F(4,111) = 3.61, p < 0.01) of any species examined. Males were correctly assigned to this species in DFA 100% of the time. Females were not included in DFA with other species due to insufficient sample size.

#### Remarks

Our DFA showed that males of this species are very distinct in morphospace from other members of this species group (Fig 7), with individuals correctly assigned 100% of the time. Sample size of females was too small for DFA.

## Discussion

We present evidence to reject the hypothesis that frogs that morphologically resemble *S*. *nigrovittata* should continue to be recognized as a single species. Instead, we hypothesize that these frogs represent a minimum of eight distinct evolutionary lineages that should be recognized as species. Our evidence for their recognition as species is based on three independent lines of evidence, morphology (both qualitative and quantitative measures of phenotype), mtDNA, and/or nuDNA. Mitochondrial DNA divergence provided us with a framework for initially partitioning up this complex, and those mitochondrial lineages corroborated by morphological or nuDNA divergence were recognized and named herein. Numerous subclades of mtDNA (Figs 1–4) were not recognized herein as species. Three previously named species (*S*. *nigrovittata*, *S*. *mortenseni*, and *S*. *faber*) and two newly-named species (*S*. *malayana* sp. nov. and *S*. *lacrima* sp. nov.) were readily distinguishable using the data that were available to us. Three of the newly named species (*S*. *annamitica* sp. nov., *S*. *montosa* sp. nov., and *S*. *roberti* sp. nov.) were less readily distinguishable from *S*. *nigrovittata* and *S*. *mortenseni*, but sufficient evidence existed in our view to support the hypothesis that these three also represented distinct species. These three named taxa may be less easily diagnosed because they are younger evolutionary lineages, or incipient species, that continue to share ancestral polymorphisms in nuDNA and morphology with *S*. *nigrovittata* or *S*. *mortenseni*.

We emphasize that our recognized eight species are hypotheses that remain testable with new data. Finer-scale, denser sampling at the geographic boundaries of the proposed species, accompanied by the use of multi-locus data sets to delimit species using coalescent-based methods such as *BEAST [68] or Bayesian Phylogenetics & Phylogeography [69], should be employed to further test these hypotheses of species. Acoustic data, such as advertisement calls of males, should also be compared at these species boundaries. The presence of more than one species in at least partial sympatry (central Laos) and syntopy (southwestern Cambodia) suggests that isolating mechanisms between at least some of these species probably exist. Studies of acoustic variation, and other parameters such as timing of reproduction, mode of reproduction, and microhabitat use of different life stages (larvae and adults) may provide important insight into these isolating mechanisms and further clarify species boundaries in this group of frogs. We did not study larvae in this group, as we lacked sufficient material, and previously published descriptions of larvae from across the geographic range of the species complex have found great similarities in larval shape, size, coloration, and oral characters among populations [30, 66, 70]. However, this study should provide a framework for new, targeted sampling of larvae, with identifications verified with DNA barcoding methods [e.g. 30], that are then studied using tools such as scanning electron microscopy of the buccopharyngeal morphology, a method that has been shown to be useful in revealing differences in larval characters of otherwise morphologically cryptic species [71].

The *S*. *nigrovittata* complex is taxonomically challenging and ripe with morphologically cryptic species that are difficult to tease apart; for these reasons, *S*. *nigrovittata* has been recognized as being polytypic for nearly a century, but without formal description of much of this variation [24, 26–30, 72]. Nonetheless, our findings are largely consistent with those of others, although the interpretations of the same variation across the range of “*S*. *nigrovittata*” may differ. Smith [26] noted three distinct morphotypes within Thailand, and these largely agree with our hypotheses of at least three species occurring in the country (*S*. *nigrovittata*, *S*. *mortenseni*, and *S*. *malayana* sp. nov.). Matsui et al. [27], using allozymic data, found that populations of *S*. *mortenseni* at Koh Chang, Thailand (its type locality) and *S*. *nigrovittata* at Phu Wua in northeastern Thailand formed a group that was separate from populations at Phu Luang and elsewhere in northern Thailand. They used these findings to justify treating *S*. *mortenseni* as a junior synomym of *S*. *nigrovittata*. We found the same general pattern of genetic (and morphological) variation, but instead expanded the range of *S*. *mortenseni* into northeastern Thailand [as did 28], and assigned samples from Phu Luang (and elsewhere in northern Thailand) to *S*. *nigrovittata* sensu stricto. Gawor et al.’s [30] recovery of distinct mitochondrial DNA clades in central Vietnam and northern Thailand is also corroborated in our study.

One of the most compelling justifications for rejecting the hypothesis that *S*. *nigrovittata* contains a single species is the non-monophyly of frogs that morphologically resemble *S*. *nigrovittata* to other species of *Sylvirana* [29, this study]. To facilitate improved communication, we recommend modifying the concept of the *S*. *nigrovittata* complex from that employed at the start of our study [and that by 29] to one that reflects only those lineages that form a monophyletic group with the nominate form, *S*. *nigrovittata*. As such, we recommend not continuing to treat *S*. *lacrima* sp. nov. as a member of the *S*. *nigrovittata* complex, despite the general similarity in its morphology that led to its inclusion in our initial study and that by Oliver et al. [29].

Our hypotheses of species in this complex are likely an underestimate, as anecdotal evidence suggests the high likelihood of additional, unstudied diversity in the group, notably in Myanmar and Sumatra. Boulenger’s [73] syntypes of *S*. *mortenseni* from Koh Chang, Thailand, and Mon, Myanmar, were examined by us and cursorily determined to represent two morphologically distinct species. The designation of a lectotype herein from Koh Chang assigns the form from that locality to the name *S*. *mortensensi*, but we chose to not include the Mon specimens in our study (or to assign them a name) owing to the unavailability of genetic material from Mon or its vicinity. In addition, a single specimen of *S*. “*nigrovittata*” from off of the mainland of Southeast Asia, in Sumatra, Indonesia, was cursorily examined by us (FMNH 210049), but was also not included in this study owing to lack of accompanying genetic material. Conversely, two species in this complex from southern China that were recently named as new species without genetic evidence, *S*. *menglaensis* and *S*. *hekouensis* (Fei, Ye & Xie, 2008; Fei, Ye & Jiang, 2008) were synonymized by us with *S*. *nigrovittata* on the basis of samples available to us from nearby localities in Laos and Vietnam. However, these synonymies should be verified with additional sampling at, or much closer to, their type localities. Further sampling in Myanmar, Sumatra, and southern China, and study of the resulting collections, may reveal additional diversity and/or help to clarify species boundaries in this group.

While *S*. *nigrovittata* sensu stricto remains geographically widespread in our study, the other members of this group have considerably smaller geographic ranges. *Sylvirana nigrovittata* is currently listed as a species of global Least Concern [23], in part due to its wide geographic range [74]. However, *S*. *mortenseni* and the five species newly described here need a reassessment of their conservation status. For example, *S*. *lacrima* sp. nov. and *S*. *roberti* sp. nov., are currently known only from two localities each and so may have acute conservation needs. Conversely, *S*. *mortenseni* was listed as Near Threatened [23] based on having a known geographic range of only Koh Chang Island, Thailand, and the adjacent Cardamom Mountains of Cambodia at the time of the assessment [75]. Our study provided evidence that the geographic distribution of this species is much larger than previously realized (Fig 11; and see [28]), and thus *S*. *mortenseni* could warrant a downgrade in its conservation assessment. Regardless of size of geographic range, all species here suffer habitat loss, as deforestation rates in Southeast Asia continue to be among the highest globally [18]. The threat of habitat loss is particularly acute for those species having known narrow geographic distributions (e.g., *Sylvirana lacrima* sp. nov., *S*. *roberti* sp. nov., and *S*. *annamitica* sp. nov.). This underscores the importance of properly delimiting species in complexes of cryptic taxa, as well as their geographic ranges, in order to accurately assess conservation priorities within countries and across the region.

## Supporting information

S1 Table Samples used in molecular analyses. (DOCX)

S2 Table Samples of *Sylvirana* used in morphological analyses. (DOCX)

S1 Appendix Measurements of *Sylvirana* specimens used in morphological analyses. (XLSX)
