# Correction: Hidden species diversity in *Sylvirana nigrovittata* (Amphibia: Ranidae) highlights the importance of taxonomic revisions in biodiversity conservation

**DOI:** 10.1371/journal.pone.0196242

**Published:** 2018-04-17

**Authors:** The PLOS ONE Staff

## Notice of republication

This article was republished on April 5, 2018, to correct an error in Fig 11. The publisher apologizes for the error. Please download this article again to view the correct version. The originally published, uncorrected article and the republished, corrected article are provided here for reference.

## Supporting information

S1 File Originally published, uncorrected article. (PDF)

S2 File Republished, corrected article. (PDF)
