# Supplementary material for: Hidden species diversity in Sylvirana nigrovittata (Amphibia: Ranidae) highlights the importance of taxonomic revisions in biodiversity conservation
Source: PLoS One. 2018 Mar 14;13(3):e0192766. doi: 10.1371/journal.pone.0192766 (PMC5851555; doi:10.1371/journal.pone.0192766)
Supplement: S1 Table — (DOCX) [file pone.0192766.s001.docx]

**S1 Table. Samples used in molecular analyses.** Bolded latitude and longitude indicate estimates by the authors based on stated collection localities. All other coordinates provided by respective museum.

| **Taxon** | **ID** | **Catalog No.** | **Field No.** | **Locality/Source** | **Latitude (N)** | **Longitude (E)** | **ND3** | **ND2** | **16S** | **POMC** | **NCX1** |
| --- | --- | --- | --- | --- | --- | --- | --- | --- | --- | --- | --- |
| *Babina holsti* | 1 | n/a | n/a | GenBank | n/a | n/a | AB761264 | AB761264 | AB761264 | AB777228 | AB777230 |
| *Hydrophylax leptoglossa* | 2 | CAS 240980 | CAS-MHS-25346 | Myitkyina District, Kachin State, Myanmar | 24.9640 | 96.3424 | MG606992 | MG606659 | MG606312 | MG605975 | MG605710 |
| *Hylarana erythraea* | 3 | FMNH 257282 | HKV 63681 | Siem Reap District, Siem Reap Province, Cambodia | 13.3747 | 103.8456 | MG606995 | MG606662 | MG606315 | MG605978 | MG605713 |
| *Indosylvirana attigua* | 4 | FMNH 258259 | HKV 64054 | Pakxong District, Champasak Province, Laos | 15.0653 | 106.2175 | MG606993 | MG606660 | MG606313 | MG605976 | MG605711 |
| *Pelophylax nigromaculatus* | 5 | n/a | n/a | GenBank | n/a | n/a | KT878718 | KT878718 | KT878718 | GU978207 | AB526633 |
| *Sylvirana annamitica* sp. nov. | 6 | AMNH A-161290 | AMNH FS-13559 (AMCC 106394) | Rao Cai, Ke Go Natural Reserve, Ha Tinh Province, Vietnam | **18.0667** | **106.0333** | MG607016 | MG606682 | MG606336 | MG606001 | n/a |
| *S. annamitica* sp. nov*.* | 7 | AMNH A-161461 | AMNH FS-13105 (AMCC 106428) | Ba Be District, Bac Kan Province, Vietnam | 22.4003 | 105.6317 | MG607322 | MG606978 | MG606645 | n/a | n/a |
| *S. annamitica* sp. nov*.* | 8 | AMNH A-169308 | AMNH FS-16642 (AMCC 144885) | A Luoi District, Thua Tien Hue Province, Vietnam | 16.2606 | 107.4439 | MG607022 | MG606688 | MG606342 | MG606005 | n/a |
| *S. annamitica* sp. nov. | 9 | AMNH A-181997 | AMNH FS-12816 (AMCC 192773) | Phuoc Son District, Quang Nam Province, Vietnam | 15.3439 | 107.7358 | MG607253 | MG606919 | MG606575 | MG606239 | MG605906 |
| *S. annamitica* sp. nov. | 10 | AMNH A-500117 | AMNH FS-12809 (AMCC 192765) | Phuoc Son District, Quang Nam Province, Vietnam | 15.3439 | 107.7358 | MG607252 | MG606918 | MG606574 | MG606238 | MG605905 |
| *S. annamitica* sp. nov. | 11 | AMNH A-501128 | AMNH FS-16644 (AMCC 144887) | A Luoi District, Thua Tien Hue Province, Vietnam | **16.2606** | **107.4439** | MG607250 | MG606916 | MG606572 | MG606236 | MG605903 |
| *S. annamitica* sp. nov. | 12 | AMNH A-501129 | AMNH FS-16645 (AMCC 144888) | A Luoi District, Thua Tien Hue Province, Vietnam | **16.2606** | **107.4439** | MG607251 | MG606917 | MG606573 | MG606237 | MG605904 |
| *S. annamitica* sp. nov. | 13 | AMNH A-501126 | AMNH FS-16800 (AMCC 144802) | Nam Dong District, Thua Tien Hue Province, Vietnam | **16.1278** | **107.7456** | MG607249 | MG606915 | MG606571 | MG606235 | MG605902 |
| *S. annamitica* sp. nov. | 14 | FMNH 255627 | HKV 62628 | Con Cuong District, Nghe An Province, Vietnam | 18.9667 | 104.8000 | MG607323 | MG606979 | MG606646 | MG606046 | MG605774 |
| *S. annamitica* sp. nov. | 15 | FMNH 255628 | HKV 62629 | Con Cuong District, Nghe An Province, Vietnam | 18.9667 | 104.8000 | MG607324 | MG606980 | MG606647 | MG606047 | MG605775 |
| *S. annamitica* sp. nov. | 16 | FMNH 255629 | HKV 62686 | Con Cuong District, Nghe An Province, Vietnam | 18.9333 | 104.7500 | MG607325 | MG606981 | MG606648 | MG606048 | MG605776 |
| *S. annamitica* sp. nov. | 17 | FMNH 255630 | HKV 62687 | Con Cuong District, Nghe An Province, Vietnam | 18.9333 | 104.7500 | MG607326 | MG606982 | MG606649 | MG606049 | MG605777 |
| *S. annamitica* sp. nov. | 18 | FMNH 255631 | HKV 62737 | Tuong Dong District, Nghe An Province, Vietnam | 19.0500 | 104.6167 | MG607327 | MG606983 | MG606650 | MG606050 | MG605778 |
| *S. annamitica* sp. nov. | 19 | FMNH 256533 | HKV 62825 | Nakai District, Khammouan Province, Laos | 17.9500 | 105.5667 | MG607331 | MG606987 | MG606654 | MG606054 | n/a |
| *S. annamitica* sp. nov. | 20 | FMNH 256535 | HKV 62789 | Nakai District, Khammouan Province, Laos | 17.9667 | 105.5667 | MG607328 | MG606984 | MG606651 | MG606051 | MG605779 |
| *S. annamitica* sp. nov. | 21 | FMNH 256538 | HKV 62801 | Nakai District, Khammouan Province, Laos | 17.9667 | 105.5667 | MG607329 | MG606985 | MG606652 | MG606052 | MG605780 |
| *S. annamitica* sp. nov. | 22 | FMNH 256539 | HKV 62802 | Nakai District, Khammouan Province, Laos | 17.9667 | 105.5667 | MG607330 | MG606986 | MG606653 | MG606053 | MG605781 |
| *S. annamitica* sp. nov. | 23 | FMNH 256540 | HKV 62842 | Nakai District, Khammouan Province, Laos | 17.9500 | 105.5667 | MG607332 | MG606988 | MG606655 | MG606055 | MG605782 |
| *S. annamitica* sp. nov. | 24 | NCSM 79174 | AMNH FS-12749 (AMCC 192686 ) | Nam Giang District, Quang Nam Province, Vietnam | 15.6591 | 107.6015 | MG607297 | MG606953 | MG606620 | MG606286 | MG605953 |
| *S. annamitica* sp. nov. | 25 | ROM 27786 | ROM 14398 | Tam Dao District, Vinh Phu Province, Vietnam | 21.4542 | 105.6414 | MG607334 | MG606990 | MG606657 | n/a | n/a |
| *S. annamitica* sp. nov. | 26 | ZFMK (catalog no. unknown) | TZ 201 | Ky Anh District, Ha Tinh Province, Vietnam | **18.0064** | **106.1257** | MG607333 | MG606989 | MG606656 | MG606311 | MG605974 |
| *S. cubitalis* | 27 | FMNH 265818 | HKV 65999 | Phu Rua District, Loei Province, Thailand | 17.3341 | 101.5090 | MG606994 | MG606661 | MG606314 | MG605977 | MG605712 |
| *S. faber* | 28 | FMNH 262621 | HKV 64469 | Kampot District, Kampot Province, Cambodia | 10.6314 | 104.0425 | MG606998 | MG606665 | MG606318 | MG605981 | MG605716 |
| *S. faber* | 29 | FMNH 262622 | HKV 64470 | Kampot District, Kampot Province, Cambodia | 10.6314 | 104.0425 | MG606999 | MG606666 | MG606319 | MG605982 | MG605717 |
| *S. faber* | 30 | FMNH 262623 | HKV 64471 | Kampot District, Kampot Province, Cambodia | 10.6314 | 104.0425 | MG607000 | MG606667 | MG606320 | MG605983 | n/a |
| *S. faber* | 31 | FMNH 262624 | HKV 64472 | Kampot District, Kampot Province, Cambodia | 10.6314 | 104.0425 | MG607001 | MG606668 | MG606321 | MG605984 | MG605718 |
| *S. faber* | 32 | FMNH 262625 | HKV 64473 | Kampot District, Kampot Province, Cambodia | 10.6314 | 104.0425 | MG607002 | MG606669 | MG606322 | MG605985 | MG605719 |
| *S. faber* | 33 | FMNH 262626 | HKV 64513 | Kampot District, Kampot Province, Cambodia | 10.6219 | 104.0478 | MG607003 | MG606670 | MG606323 | MG605986 | MG605720 |
| *S. faber* | 34 | FMNH 262627 | HKV 64514 | Kampot District, Kampot Province, Cambodia | 10.6219 | 104.0478 | MG607004 | MG606671 | MG606324 | MG605987 | MG605721 |
| *S. faber* | 35 | FMNH 262628 | HKV 64515 | Kampot District, Kampot Province, Cambodia | 10.6219 | 104.0478 | MG607005 | MG606672 | MG606325 | MG605988 | MG605722 |
| *S. faber* | 36 | FMNH 262629 | HKV 64516 | Kampot District, Kampot Province, Cambodia | 10.6219 | 104.0478 | MG607006 | MG606673 | MG606326 | MG605989 | MG605723 |
| *S. faber* | 37 | FMNH 262630 | HKV 64517 | Kampot District, Kampot Province, Cambodia | 10.6219 | 104.0478 | MG607007 | MG606674 | MG606327 | MG605990 | MG605724 |
| *S. faber* | 38 | FMNH 262631 | HKV 64536 | Kampot District, Kampot Province, Cambodia | 10.6314 | 104.0425 | MG607008 | MG606675 | MG606328 | MG605991 | MG605725 |
| *S. faber* | 39 | FMNH 262632 | HKV 64559 | Kampot District, Kampot Province, Cambodia | 10.6219 | 104.0478 | MG607009 | MG606676 | MG606329 | MG605992 | MG605726 |
| *S. faber* | 40 | FMNH 262633 | HKV 64560 | Kampot District, Kampot Province, Cambodia | 10.6219 | 104.0478 | MG607010 | MG606677 | MG606330 | MG605993 | MG605727 |
| *S. faber* | 41 | FMNH 267767 | HKV 33198 | Pursat Province, Cambodia | **12.0933** | **104.2736** | MG606997 | MG606664 | MG606317 | MG605980 | MG605715 |
| *S. faber* | 42 | FMNH 267772 | HKV 33176 | Pursat Province, Cambodia | **11.8647** | **103.6623** | MG606996 | MG606663 | MG606316 | MG606008 | MG605738 |
| *S. lacrima* sp. nov. | 43 | CAS 231229 | JBS 14746 | Nyaung-U District, Mandalay State, Myanmar | 20.9046 | 95.2360 | MG607263 | MG606930 | MG606586 | MG606252 | MG605919 |
| *S. lacrima* sp. nov. | 44 | CAS 231310 | JBS 14921 | Nyaung-U District, Mandalay State, Myanmar | 20.9019 | 95.2335 | MG607264 | MG606931 | MG606587 | MG606253 | MG605920 |
| *S. lacrima* sp. nov. | 45 | CAS 232307 | JBS 14920 | Nyaung-U District, Mandalay State, Myanmar | 20.9019 | 95.2335 | MG607265 | MG606932 | MG606588 | MG606254 | MG605921 |
| *S. lacrima* sp. nov. | 46 | CAS 234925 | JBS 25034 | Mintatt District, Chin State, Myanmar | 21.3868 | 94.0567 | MG607267 | MG606934 | MG606590 | MG606256 | MG605923 |
| *S. lacrima* sp. nov. | 47 | CAS 234990 | JBS 25162 | Mintatt District, Chin State, Myanmar | 21.7807 | 93.7482 | MG607268 | MG606935 | MG606591 | MG606257 | MG605924 |
| *S. lacrima* sp. nov. | 48 | CAS 235166 | JBS 25397 | Mintatt District, Chin State, Myanmar | 21.2715 | 93.7523 | MG607269 | MG606936 | MG606592 | MG606258 | MG605925 |
| *S. malayana* sp. nov. | 49 | CAS 247472 | CAS-MHS-30677 | Kawthaung District, Tanintharyi State, Myanmar | 10.3720 | 98.6086 | MG607272 | MG606939 | MG606595 | MG606261 | MG605928 |
| *S. malayana* sp. nov. | 50 | CAS 247856 | CAS-MHS-30908 | Kawthaung District, Tanintharyi State, Myanmar | 10.3698 | 98.6313 | MG607273 | MG606940 | MG606596 | MG606262 | MG605929 |
| *S. malayana* sp. nov. | 51 | CAS 247866 | CAS-MHS-30942 | Kawthaung District, Tanintharyi State, Myanmar | 10.3794 | 98.6092 | MG607274 | MG606941 | MG606597 | MG606263 | MG605930 |
| *S. malayana* sp. nov. | 52 | FMNH 268384 | HKV 66726 | Ranong District, Ranong Province, Thailand | **9.8562** | **98.6276** | MG607238 | MG606904 | MG606560 | MG606224 | MG605897 |
| *S. malayana* sp. nov. | 53 | FMNH 268385 | HKV 66800 | Khlong Sok District, Surat Thani Province, Thailand | **9.5062** | **98.8014** | MG607239 | MG606905 | MG606561 | MG606225 | n/a |
| *S. malayana* sp. nov. | 54 | FMNH 268386 | HKV 66804 | Khlong Sok District, Surat Thani Province, Thailand | **9.5062** | **98.8014** | MG607240 | MG606906 | MG606562 | MG606226 | MG605898 |
| *S. malayana* sp. nov. | 55 | FMNH 268387 | HKV 66806 | Khlong Sok District, Surat Thani Province, Thailand | **9.5062** | **98.8014** | MG607241 | MG606907 | MG606563 | MG606227 | n/a |
| *S. malayana* sp. nov. | 56 | FMNH 268388 | HKV 66830 | Khlong Sok District, Surat Thani Province, Thailand | **9.5062** | **98.8014** | MG607242 | MG606908 | MG606564 | MG606228 | MG605899 |
| *S. malayana* sp. nov. | 57 | FMNH 268389 | HKV 66831 | Khlong Sok District, Surat Thani Province, Thailand | **9.5062** | **98.8014** | MG607243 | MG606909 | MG606565 | MG606229 | n/a |
| *S. malayana* sp. nov. | 58 | FMNH 268390 | HKV 66832 | Khlong Sok District, Surat Thani Province, Thailand | **9.5062** | **98.8014** | MG607244 | MG606910 | MG606566 | MG606230 | n/a |
| *S. malayana* sp. nov. | 59 | FMNH 268391 | HKV 66834 | Khlong Sok District, Surat Thani Province, Thailand | **9.5062** | **98.8014** | MG607245 | MG606911 | MG606567 | MG606231 | MG605900 |
| *S. malayana* sp. nov. | 60 | FMNH 268392 | HKV 66840 | Khlong Sok District, Surat Thani Province, Thailand | **9.5062** | **98.8014** | MG607246 | MG606912 | MG606568 | MG606232 | MG605901 |
| *S. malayana* sp. nov. | 61 | FMNH 268393 | HKV 66841 | Khlong Sok District, Surat Thani Province, Thailand | **9.5062** | **98.8014** | MG607247 | MG606913 | MG606569 | MG606233 | n/a |
| *S. malayana* sp. nov. | 62 | FMNH 268394 | HKV 66842 | Khlong Sok District, Surat Thani Province, Thailand | **9.5062** | **98.8014** | MG607248 | MG606914 | MG606570 | MG606234 | n/a |
| *S. malayana* sp. nov. | 63 | FMNH 268763 | RFI 50886 | Krabi Province, Thailand | **9.4792** | **98.9400** | MG607037 | MG606703 | MG606357 | MG606021 | MG605751 |
| *S. malayana* sp. nov. | 64 | FMNH 268764 | RFI 50889 | Krabi Province, Thailand | **9.4792** | **98.9400** | MG607038 | MG606704 | MG606358 | MG606022 | MG605752 |
| *S. malayana* sp. nov. | 65 | FMNH 268765 | RFI 50890 | Krabi Province, Thailand | **9.4792** | **98.9400** | MG607039 | MG606705 | MG606359 | MG606023 | MG605753 |
| *S. malayana* sp. nov. | 66 | FMNH 268766 | RFI 50893 | Krabi Province, Thailand | **8.2726** | **98.9216** | MG607041 | MG606707 | MG606361 | MG606025 | MG605755 |
| *S. malayana* sp. nov. | 67 | FMNH 268767 | RFI 50917 | Krabi Province, Thailand | **8.2726** | **98.9216** | MG607045 | MG606711 | MG606365 | MG606029 | MG605759 |
| *S. malayana* sp. nov. | 68 | FMNH 268768 | RFI 50931 | Krabi Province, Thailand | **7.8873** | **99.2901** | MG607047 | MG607337 | n/a | MG606031 | MG605761 |
| *S. malayana* sp. nov. | 69 | FMNH 268770 | RFI 51021 | Khlong Sok District, Surat Thani Province, Thailand | **8.9147** | **98.5278** | MG607049 | MG606713 | MG606368 | MG606033 | MG605763 |
| *S. malayana* sp. nov. | 70 | FMNH 268771 | RFI 51051 | Khlong Sok District, Surat Thani Province, Thailand | **8.9579** | **98.5846** | MG607052 | MG606716 | MG606371 | MG606036 | MG605766 |
| *S. malayana* sp. nov. | 71 | FMNH 268772 | RFI 51053 | Khlong Sok District, Surat Thani Province, Thailand | **8.9147** | **98.5278** | MG607053 | MG606717 | MG606372 | MG606037 | MG605767 |
| *S. malayana* sp. nov. | 72 | FMNH 268969 | RFI 50885 | Krabi Province, Thailand | **8.2726** | **98.9216** | MG607036 | MG606702 | MG606356 | MG606020 | MG605750 |
| *S. malayana* sp. nov. | 73 | FMNH 268970 | RFI 50892 | Krabi Province, Thailand | **9.4792** | **98.9400** | MG607040 | MG606706 | MG606360 | MG606024 | MG605754 |
| *S. malayana* sp. nov. | 74 | FMNH 268971 | RFI 50895 | Krabi Province, Thailand | **9.4792** | **98.9400** | MG607042 | MG606708 | MG606362 | MG606026 | MG605756 |
| *S. malayana* sp. nov. | 75 | FMNH 268972 | RFI 50896 | Krabi Province, Thailand | **9.4792** | **98.9400** | MG607043 | MG606709 | MG606363 | MG606027 | MG605757 |
| *S. malayana* sp. nov. | 76 | FMNH 268973 | RFI 50897 | Krabi Province, Thailand | **9.4792** | **98.9400** | MG607044 | MG606710 | MG606364 | MG606028 | MG605758 |
| *S. malayana* sp. nov. | 77 | FMNH 268974 | RFI 50927 | Krabi Province, Thailand | **7.8873** | **99.2901** | MG607046 | MG607336 | MG606366 | MG606030 | MG605760 |
| *S. malayana* sp. nov. | 78 | FMNH 268975 | RFI 51019 | Khlong Sok District, Surat Thani Province, Thailand | **8.9147** | **98.5278** | MG607048 | MG606712 | MG606367 | MG606032 | MG605762 |
| *S. malayana* sp. nov. | 79 | FMNH 268976 | RFI 51022 | Khlong Sok District, Surat Thani Province, Thailand | **8.9579** | **98.5846** | MG607050 | MG606714 | MG606369 | MG606034 | MG605764 |
| *S. malayana* sp. nov. | 80 | FMNH 268977 | RFI 51030 | Khlong Sok District, Surat Thani Province, Thailand | **8.9579** | **98.5846** | MG607051 | MG606715 | MG606370 | MG606035 | MG605765 |
| *S. malayana* sp. nov. | 81 | FMNH 268978 | RFI 51054 | Khlong Sok District, Surat Thani Province, Thailand | **8.9579** | **98.5846** | MG607054 | MG606718 | MG606373 | MG606038 | MG605768 |
| *S. malayana* sp. nov. | 82 | FMNH 268979 | RFI 51055 | Khlong Sok District, Surat Thani Province, Thailand | **8.9147** | **98.5278** | MG607055 | MG606719 | MG606374 | MG606039 | MG605769 |
| *S. malayana* sp. nov. | 83 | LSUHC 5854 | n/a | Temengor District, Perak State, Malaysia | 3.9433 | 103.0878 | MG607282 | MG607338 | MG606605 | MG606271 | MG605938 |
| *S. malayana* sp. nov. | 84 | LSUHC 5855 | n/a | Temengor District, Perak State, Malaysia | 3.9433 | 103.0878 | MG607283 | MG607339 | MG606606 | MG606272 | MG605939 |
| *S. malayana* sp. nov. | 85 | LSUHC 5856 | n/a | Temengor District, Perak State, Malaysia | 3.9433 | 103.0878 | MG607284 | MG607340 | MG606607 | MG606273 | MG605940 |
| *S. malayana* sp. nov. | 86 | LSUHC 5953 | n/a | Jerai District, Kedah State, Malaysia | **5.7913** | **100.4320** | MG607285 | MG607341 | MG606608 | MG606274 | MG605941 |
| *S. malayana* sp. nov. | 87 | LSUHC 5959 | n/a | Jerai District, Kedah State, Malaysia | **5.7913** | **100.4320** | MG607286 | MG607342 | MG606609 | MG606275 | MG605942 |
| *S. malayana* sp. nov. | 88 | LSUHC 5960 | n/a | Jerai District, Kedah State, Malaysia | **5.7913** | **100.4320** | MG607287 | MG607343 | MG606610 | MG606276 | MG605943 |
| *S. malayana* sp. nov. | 89 | LSUHC 5964 | n/a | Jerai District, Kedah State, Malaysia | **5.7913** | **100.4320** | MG607288 | MG607344 | MG606611 | MG606277 | MG605944 |
| *S. malayana* sp. nov. | 90 | LSUHC 5965 | n/a | Jerai District, Kedah State, Malaysia | **5.7913** | **100.4320** | MG607289 | MG607345 | MG606612 | MG606278 | MG605945 |
| *S. malayana* sp. nov*.* | 91 | LSUHC 6001 | n/a | Jerai District, Kedah State, Malaysia | **5.7913** | **100.4320** | MG607290 | MG607346 | MG606613 | MG606279 | MG605946 |
| *S. malayana* sp. nov. | 92 | LSUHC 6002 | n/a | Jerai District, Kedah State, Malaysia | **5.7913** | **100.4320** | MG607291 | MG607347 | MG606614 | MG606280 | MG605947 |
| *S. malayana* sp. nov. | 93 | LSUHC 6003 | n/a | Jerai District, Kedah State, Malaysia | **5.7913** | **100.4320** | MG607292 | MG607348 | MG606615 | MG606281 | MG605948 |
| *S. maosonensis* | 94 | FMNH 255637 | HKV 62643 | Con Cuong District, Nghe An Province, Vietnam | **19.0633** | **104.7522** | MG607335 | MG606991 | MG606658 | MG605979 | MG605714 |
| *S. montosa* sp. nov. | 95 | AMS R184961 | BLS 11911 | Lac Duong District, Lam Dong Province, Vietnam | 12.1826 | 108.6800 | MG607020 | MG606686 | MG606340 | MG606240 | MG605907 |
| *S. montosa* sp. nov. | 96 | AMS R184960 | BLS 11913 | Lac Duong District, Lam Dong Province, Vietnam | 12.1622 | 108.6650 | MG607254 | MG606920 | MG606576 | MG606242 | MG605909 |
| *S. montosa* sp. nov. | 97 | FMNH 253792 | HKV 60820 | An Khe District, Gia Lai Province, Vietnam | 14.3333 | 108.6000 | MG607056 | MG606720 | MG606375 | MG606040 | MG605770 |
| *S. montosa* sp. nov. | 98 | FMNH 253793 | HKV 60821 | An Khe District, Gia Lai Province, Vietnam | 14.3333 | 108.6000 | MG607057 | MG606721 | MG606376 | MG606041 | MG605771 |
| *S. montosa* sp. nov. | 99 | FMNH 253794 | HKV 60935 | An Khe District, Gia Lai Province, Vietnam | 14.3333 | 108.6000 | MG607058 | MG606722 | MG606377 | MG606042 | MG605772 |
| *S. montosa* sp. nov. | 100 | FMNH 253795 | HKV 60936 | An Khe District, Gia Lai Province, Vietnam | 14.3333 | 108.6000 | MG607059 | MG606723 | MG606378 | MG606043 | n/a |
| *S. montosa* sp. nov. | 101 | FMNH 253796 | HKV 60937 | An Khe District, Gia Lai Province, Vietnam | 14.3333 | 108.6000 | MG607060 | MG606724 | MG606379 | MG606044 | MG605773 |
| *S. montosa* sp. nov. | 102 | FMNH 254752 | HKV 62211 | Khamkeut District, Bolikhamxay Province, Laos | **18.1964** | **104.9675** | MG607061 | n/a | MG606380 | n/a | n/a |
| *S. montosa* sp. nov. | 103 | FMNH 255349 | HKV 63187 | Boualapha District, Khammouan Province, Laos | 17.2833 | 105.6833 | MG607069 | MG606732 | MG606388 | MG606062 | n/a |
| *S. montosa* sp. nov. | 104 | FMNH 255350 | HKV 63188 | Boualapha District, Khammouan Province, Laos | 17.2833 | 105.6833 | MG607070 | MG606733 | MG606389 | MG606063 | n/a |
| *S. montosa* sp. nov. | 105 | FMNH 255415 | HKV 63101 | Boualapha District, Khammouan Province, Laos | 17.5000 | 105.8500 | MG607066 | MG606729 | MG606385 | MG606059 | n/a |
| *S. montosa* sp. nov. | 106 | FMNH 255417 | HKV 63112 | Boualapha District, Khammouan Province, Laos | 17.3833 | 105.7500 | MG607067 | MG606730 | MG606386 | MG606060 | n/a |
| *S. montosa* sp. nov. | 107 | FMNH 255418 | HKV 63158 | Boualapha District, Khammouan Province, Laos | 17.3333 | 105.6833 | MG607068 | MG606731 | MG606387 | MG606061 | n/a |
| *S. montosa* sp. nov. | 108 | FMNH 255419 | HKV 63189 | Boualapha District, Khammouan Province, Laos | 17.2833 | 105.6833 | MG607071 | MG606734 | MG606390 | MG606064 | n/a |
| *S. montosa* sp. nov. | 109 | FMNH 255420 | HKV 63199 | Boualapha District, Khammouan Province, Laos | 17.8500 | 104.8667 | MG607072 | MG606735 | MG606391 | MG606065 | MG605786 |
| *S. montosa* sp. nov. | 110 | FMNH 255421 | HKV 63200 | Boualapha District, Khammouan Province, Laos | 17.8500 | 104.8667 | MG607073 | MG606736 | MG606392 | MG606066 | MG605787 |
| *S. montosa* sp. nov. | 111 | FMNH 255422 | HKV 63201 | Boualapha District, Khammouan Province, Laos | 17.8500 | 104.8667 | MG607074 | MG606737 | MG606393 | MG606067 | MG605788 |
| *S. montosa* sp. nov. | 112 | FMNH 255423 | HKV 63202 | Boualapha District, Khammouan Province, Laos | 17.8500 | 104.8667 | MG607075 | MG606738 | MG606394 | MG606068 | MG605789 |
| *S. montosa* sp. nov. | 113 | FMNH 255424 | HKV 63203 | Boualapha District, Khammouan Province, Laos | 17.8500 | 104.8667 | MG607076 | MG606739 | MG606395 | MG606069 | MG605790 |
| *S. montosa* sp. nov. | 114 | FMNH 255425 | HKV 63215 | Boualapha District, Khammouan Province, Laos | 17.8500 | 104.8667 | MG607077 | MG606740 | MG606396 | MG606070 | MG605791 |
| *S. montosa* sp. nov. | 115 | FMNH 255426 | HKV 63216 | Boualapha District, Khammouan Province, Laos | 17.8500 | 104.8667 | MG607078 | MG606741 | MG606397 | MG606071 | MG605792 |
| *S. montosa* sp. nov. | 116 | FMNH 255427 | HKV 63254 | Boualapha District, Khammouan Province, Laos | 17.8833 | 104.9167 | MG607079 | MG606742 | MG606398 | MG606072 | MG605793 |
| *S. montosa* sp. nov. | 117 | FMNH 255428 | HKV 63297 | Thakhek District, Khammouan Province, Laos | 17.5500 | 104.8667 | MG607080 | MG606743 | MG606399 | MG606073 | MG605794 |
| *S. montosa* sp. nov. | 118 | FMNH 256534 | HKV 62888 | Nakai District, Khammouan Province, Laos | 17.8000 | 105.5167 | MG607065 | MG606728 | MG606384 | MG606058 | MG605785 |
| *S. montosa* sp. nov. | 119 | FMNH 256536 | HKV 62874 | Nakai District, Khammouan Province, Laos | 17.8333 | 105.5833 | MG607063 | MG606726 | MG606382 | MG606056 | MG605783 |
| *S. montosa* sp. nov. | 120 | FMNH 256537 | HKV 62882 | Nakai District, Khammouan Province, Laos | 17.8333 | 105.5833 | MG607064 | MG606727 | MG606383 | MG606057 | MG605784 |
| *S. montosa* sp. nov. | 121 | FMNH 258114 | HKV 63926 | Pakxong District, Champasak Province, Laos | 15.0769 | 106.1375 | MG607099 | MG606765 | MG606421 | MG606095 | MG605808 |
| *S. montosa* sp. nov. | 122 | FMNH 258115 | HKV 63927 | Pakxong District, Champasak Province, Laos | 15.0769 | 106.1375 | MG607100 | MG606766 | MG606422 | MG606096 | n/a |
| *S. montosa* sp. nov. | 123 | FMNH 258117 | HKV 63966 | Pakxong District, Champasak Province, Laos | 15.0467 | 106.1792 | MG607103 | MG606769 | MG606425 | MG606099 | MG605809 |
| *S. montosa* sp. nov. | 124 | FMNH 258118 | HKV 63979 | Pakxong District, Champasak Province, Laos | 15.0467 | 106.1792 | MG607104 | MG606770 | MG606426 | MG606100 | MG605810 |
| *S. montosa* sp. nov. | 125 | FMNH 258119 | HKV 63982 | Pakxong District, Champasak Province, Laos | 15.0467 | 106.1792 | MG607107 | MG606773 | MG606429 | MG606103 | n/a |
| *S. montosa* sp. nov. | 126 | FMNH 258120 | HKV 64002 | Pakxong District, Champasak Province, Laos | 15.0467 | 106.1792 | MG607108 | MG606774 | MG606430 | MG606104 | MG605813 |
| *S. montosa* sp. nov. | 127 | FMNH 258121 | HKV 64003 | Pakxong District, Champasak Province, Laos | 15.0467 | 106.1792 | MG607109 | MG606775 | MG606431 | MG606105 | n/a |
| *S. montosa* sp. nov. | 128 | FMNH 258141 | HKV 64443 | Thapangthong District, Savannakhet Province, Laos | 16.1358 | 105.9178 | MG607131 | MG606798 | MG606454 | n/a | n/a |
| *S. montosa* sp. nov. | 129 | FMNH 258142 | HKV 64444 | Thapangthong District, Savannakhet Province, Laos | 16.1358 | 105.9178 | MG607132 | MG606799 | n/a | n/a | n/a |
| *S. montosa* sp. nov. | 130 | FMNH 258143 | HKV 64445 | Thapangthong District, Savannakhet Province, Laos | 16.1358 | 105.9178 | MG607133 | MG606800 | MG606455 | n/a | n/a |
| *S. montosa* sp. nov. | 131 | FMNH 258198 | HKV 63963 | Pakxong District, Champasak Province, Laos | 15.0467 | 106.1792 | MG607101 | MG606767 | MG606423 | MG606097 | n/a |
| *S. montosa* sp. nov. | 132 | FMNH 258199 | HKV 63965 | Pakxong District, Champasak Province, Laos | 15.0467 | 106.1792 | MG607102 | MG606768 | MG606424 | MG606098 | n/a |
| *S. montosa* sp. nov. | 133 | FMNH 258200 | HKV 63980 | Pakxong District, Champasak Province, Laos | 15.0467 | 106.1792 | MG607105 | MG606771 | MG606427 | MG606101 | MG605811 |
| *S. montosa* sp. nov. | 134 | FMNH 258201 | HKV 63981 | Pakxong District, Champasak Province, Laos | 15.0467 | 106.1792 | MG607106 | MG606772 | MG606428 | MG606102 | MG605812 |
| *S. montosa* sp. nov. | 135 | FMNH 258202 | HKV 64004 | Pakxong District, Champasak Province, Laos | 15.0467 | 106.1792 | MG607110 | MG606776 | MG606432 | MG606106 | MG605814 |
| *S. montosa* sp. nov. | 136 | FMNH 258203 | HKV 64005 | Pakxong District, Champasak Province, Laos | 15.0467 | 106.1792 | MG607111 | MG606777 | MG606433 | MG606107 | MG605815 |
| *S. montosa* sp. nov. | 137 | FMNH 261971 | HKV 64745 | Pichrada District, Mondolkiri Province, Cambodia | 12.5378 | 107.5333 | MG607157 | MG606824 | MG606479 | MG606144 | n/a |
| *S. montosa* sp. nov. | 138 | FMNH 261972 | HKV 64746 | Pichrada District, Mondolkiri Province, Cambodia | 12.5378 | 107.5333 | MG607158 | MG606825 | MG606480 | MG606145 | MG605837 |
| *S. montosa* sp. nov. | 139 | FMNH 261973 | HKV 64747 | Pichrada District, Mondolkiri Province, Cambodia | 12.5378 | 107.5333 | MG607159 | MG606826 | MG606481 | MG606146 | MG605838 |
| *S. montosa* sp. nov. | 140 | FMNH 261974 | HKV 64748 | Pichrada District, Mondolkiri Province, Cambodia | 12.5378 | 107.5333 | MG607160 | MG606827 | MG606482 | MG606147 | n/a |
| *S. montosa* sp. nov. | 141 | FMNH 261975 | HKV 64776 | Pichrada District, Mondolkiri Province, Cambodia | 12.5378 | 107.5333 | MG607161 | MG606828 | MG606483 | MG606148 | MG605839 |
| *S. montosa* sp. nov. | 142 | FMNH 261976 | HKV 64836 | Pichrada District, Mondolkiri Province, Cambodia | 12.5217 | 107.5536 | MG607162 | MG606829 | MG606484 | MG606149 | n/a |
| *S. montosa* sp. nov. | 143 | FMNH 261977 | HKV 64837 | Pichrada District, Mondolkiri Province, Cambodia | 12.5217 | 107.5536 | MG607163 | MG606830 | MG606485 | MG606150 | MG605840 |
| *S. montosa* sp. nov. | 144 | FMNH 261978 | HKV 64884 | Pichrada District, Mondolkiri Province, Cambodia | 12.4969 | 107.4925 | MG607164 | MG606831 | MG606486 | MG606151 | n/a |
| *S. montosa* sp. nov. | 145 | FMNH 262843 | HKV 65439 | Ta Veng District, Ratanakiri Province, Cambodia | 14.1879 | 107.2934 | MG607165 | MG606832 | MG606487 | MG606152 | MG605841 |
| *S. montosa* sp. nov. | 146 | FMNH 262844 | HKV 65452 | Ta Veng District, Ratanakiri Province, Cambodia | 14.1879 | 107.2934 | MG607166 | MG606833 | MG606488 | MG606153 | MG605842 |
| *S. montosa* sp. nov. | 147 | FMNH 262845 | HKV 65457 | Ta Veng District, Ratanakiri Province, Cambodia | 14.2001 | 107.3083 | MG607167 | MG606834 | MG606489 | MG606154 | MG605843 |
| *S. montosa* sp. nov. | 148 | FMNH 262846 | HKV 65458 | Ta Veng District, Ratanakiri Province, Cambodia | 14.2001 | 107.3083 | MG607168 | MG606835 | MG606490 | MG606155 | n/a |
| *S. montosa* sp. nov. | 149 | FMNH 262847 | HKV 65468 | Ta Veng District, Ratanakiri Province, Cambodia | 14.1879 | 107.2934 | MG607169 | MG606836 | MG606491 | MG606156 | MG605844 |
| *S. montosa* sp. nov. | 150 | FMNH 262848 | HKV 65469 | Ta Veng District, Ratanakiri Province, Cambodia | 14.1879 | 107.2934 | MG607170 | MG606837 | MG606492 | MG606157 | MG605845 |
| *S. montosa* sp. nov. | 151 | FMNH 262849 | HKV 65470 | Ta Veng District, Ratanakiri Province, Cambodia | 14.1879 | 107.2934 | MG607171 | MG606838 | MG606493 | MG606158 | MG605846 |
| *S. montosa* sp. nov. | 152 | FMNH 262850 | HKV 65486 | Ta Veng District, Ratanakiri Province, Cambodia | 14.1879 | 107.2934 | MG607172 | MG606839 | MG606494 | MG606159 | MG605847 |
| *S. montosa* sp. nov. | 153 | FMNH 262851 | HKV 65511 | Siem Pang District, Stung Treng Province, Cambodia | 14.2941 | 106.6124 | MG607173 | MG606840 | MG606495 | MG606160 | MG605848 |
| *S. montosa* sp. nov. | 154 | FMNH 262852 | HKV 65519 | Siem Pang District, Stung Treng Province, Cambodia | 14.3194 | 106.5938 | MG607174 | MG606841 | MG606496 | MG606161 | MG605849 |
| *S. montosa* sp. nov. | 155 | FMNH 262853 | HKV 65520 | Siem Pang District, Stung Treng Province, Cambodia | 14.3194 | 106.5938 | MG607175 | MG606842 | MG606497 | MG606162 | MG605850 |
| *S. montosa* sp. nov. | 156 | FMNH 262854 | HKV 65538 | Siem Pang District, Stung Treng Province, Cambodia | 14.2638 | 106.5294 | MG607176 | MG606843 | MG606498 | MG606163 | MG605851 |
| *S. montosa* sp. nov. | 157 | FMNH 262855 | HKV 65539 | Siem Pang District, Stung Treng Province, Cambodia | 14.3079 | 106.5507 | MG607177 | MG606844 | MG606499 | MG606164 | MG605852 |
| *S. montosa* sp. nov. | 158 | FMNH 262857 | HKV 65541 | Siem Pang District, Stung Treng Province, Cambodia | 14.3079 | 106.5507 | MG607178 | MG606845 | MG606500 | MG606165 | MG605853 |
| *S. montosa* sp. nov. | 159 | FMNH 262858 | HKV 65818 | O'Rang District, Mondolkiri Province, Cambodia | 12.3161 | 107.0997 | MG607180 | MG606847 | MG606502 | MG606166 | n/a |
| *S. montosa* sp. nov. | 160 | FMNH 262859 | HKV 65819 | O'Rang District, Mondolkiri Province, Cambodia | 12.3161 | 107.0997 | MG607181 | MG606848 | MG606503 | MG606167 | MG605855 |
| *S. montosa* sp. nov. | 161 | FMNH 262860 | HKV 65820 | O'Rang District, Mondolkiri Province, Cambodia | 12.3161 | 107.0997 | MG607182 | MG606849 | MG606504 | MG606168 | MG605856 |
| *S. montosa* sp. nov. | 162 | FMNH 262861 | HKV 65834 | O'Rang District, Mondolkiri Province, Cambodia | 12.3161 | 107.0997 | MG607183 | MG606850 | MG606505 | MG606169 | MG605857 |
| *S. montosa* sp. nov. | 163 | FMNH 262862 | HKV 65849 | O'Rang District, Mondolkiri Province, Cambodia | 12.3264 | 107.0925 | MG607184 | MG606851 | MG606506 | MG606170 | MG605858 |
| *S. montosa* sp. nov. | 164 | FMNH 262863 | HKV 65874 | O'Rang District, Mondolkiri Province, Cambodia | 12.2919 | 107.0517 | MG607185 | MG606852 | MG606507 | MG606171 | MG605859 |
| *S. montosa* sp. nov. | 165 | FMNH 262864 | HKV 65890 | O'Rang District, Mondolkiri Province, Cambodia | 12.2917 | 107.0517 | MG607186 | MG606853 | MG606508 | MG606172 | MG605860 |
| *S. montosa* sp. nov. | 166 | FMNH 262865 | HKV 65935 | Keo Seima District, Mondolkiri Province, Cambodia | 12.2794 | 106.9434 | MG607187 | MG606854 | MG606509 | MG606173 | MG605861 |
| *S. montosa* sp. nov. | 167 | FMNH 262866 | HKV 65936 | Keo Seima District, Mondolkiri Province, Cambodia | 12.2794 | 106.9434 | MG607188 | MG606855 | MG606510 | MG606174 | n/a |
| *S. montosa* sp. nov. | 168 | FMNH 262867 | HKV 65937 | Keo Seima District, Mondolkiri Province, Cambodia | 12.2794 | 106.9434 | MG607189 | MG606856 | MG606511 | MG606175 | MG605862 |
| *S. montosa* sp. nov. | 169 | FMNH 262868 | HKV 65938 | Keo Seima District, Mondolkiri Province, Cambodia | 12.2794 | 106.9434 | MG607190 | MG606857 | MG606512 | MG606176 | n/a |
| *S. montosa* sp. nov. | 170 | FMNH 262869 | HKV 65939 | Keo Seima District, Mondolkiri Province, Cambodia | 12.2794 | 106.9434 | MG607191 | MG606858 | MG606513 | MG606177 | n/a |
| *S. montosa* sp. nov. | 171 | FMNH 271413 | BLS 10702 | Boualapha District, Khammouan Province, Laos | 17.6253 | 105.7173 | MG607017 | MG606683 | MG606337 | MG606002 | MG605734 |
| *S. montosa* sp. nov. | 172 | FMNH 271414 | BLS 10703 | Boualapha District, Khammouan Province, Laos | 17.6253 | 105.7173 | MG607018 | MG606684 | MG606338 | MG606003 | MG605735 |
| *S. montosa* sp. nov. | 173 | FMNH 271415 | BLS 10704 | Boualapha District, Khammouan Province, Laos | 17.6253 | 105.7173 | MG607019 | MG606685 | MG606339 | MG606004 | MG605736 |
| *S. montosa* sp. nov. | 174 | NCSM 76387 | BLS 12492 | Vilabouli District, Savannakhet Province, Laos | 16.9685 | 105.8102 | MG607293 | MG606949 | MG606616 | MG606282 | MG605949 |
| *S. montosa* sp. nov. | 175 | NCSM 76392 | BLS 12777 | Vilabouli District, Savannakhet Province, Laos | 16.9718 | 106.1666 | MG607294 | MG606950 | MG606617 | MG606283 | MG605950 |
| *S. montosa* sp. nov. | 176 | NCSM 76395 | BLS 12807 | Vilabouli District, Savannakhet Province, Laos | 17.0445 | 106.1262 | MG607295 | MG606951 | MG606618 | MG606284 | MG605951 |
| *S. montosa* sp. nov. | 177 | NCSM 76398 | BLS 12825 | Vilabouli District, Savannakhet Province, Laos | 17.0118 | 106.2201 | MG607296 | MG606952 | MG606619 | MG606285 | MG605952 |
| *S. montosa* sp. nov. | 178 | NCSM 77403 | BLS 11912 | Lac Duong District, Lam Dong Province, Vietnam | 12.1622 | 108.6650 | MG607021 | MG606687 | MG606341 | MG606241 | MG605908 |
| *S. mortenseni* | 179 | FMNH 255404 | HKV 62582 | Mounlapamok District, Champasak Province, Laos | 14.1333 | 105.3667 | MG607062 | MG606725 | MG606381 | MG606045 | n/a |
| *S. mortenseni* | 180 | FMNH 255434 | HKV 63396 | Thaphabat District, Bolikhamxay Province, Laos | 18.4500 | 103.1667 | MG607349 | MG606749 | MG606405 | MG606079 | MG605797 |
| *S. mortenseni* | 181 | FMNH 255435 | HKV 63397 | Thaphabat District, Bolikhamxay Province, Laos | 18.4500 | 103.1667 | MG607350 | MG606750 | MG606406 | MG606080 | MG605798 |
| *S. mortenseni* | 182 | FMNH 255436 | HKV 63404 | Thaphabat District, Bolikhamxay Province, Laos | 18.4500 | 103.1667 | MG607351 | MG606751 | MG606407 | MG606081 | MG605799 |
| *S. mortenseni* | 183 | FMNH 255437 | HKV 63477 | Mounlapamok District, Champasak Province, Laos | 14.1167 | 105.4833 | MG607086 | MG606752 | MG606408 | MG606082 | n/a |
| *S. mortenseni* | 184 | FMNH 255438 | HKV 63478 | Mounlapamok District, Champasak Province, Laos | 14.1167 | 105.4833 | MG607087 | MG606753 | MG606409 | MG606083 | MG605800 |
| *S. mortenseni* | 185 | FMNH 255439 | HKV 63479 | Mounlapamok District, Champasak Province, Laos | 14.1167 | 105.4833 | MG607088 | MG606754 | MG606410 | MG606084 | MG605801 |
| *S. mortenseni* | 186 | FMNH 255440 | HKV 63490 | Mounlapamok District, Champasak Province, Laos | 14.1333 | 105.3667 | MG607089 | MG606755 | MG606411 | MG606085 | MG605802 |
| *S. mortenseni* | 187 | FMNH 255441 | HKV 63491 | Mounlapamok District, Champasak Province, Laos | 14.1333 | 105.3667 | MG607090 | MG606756 | MG606412 | MG606086 | n/a |
| *S. mortenseni* | 188 | FMNH 255442 | HKV 63492 | Mounlapamok District, Champasak Province, Laos | 14.1333 | 105.3667 | MG607091 | MG606757 | MG606413 | MG606087 | MG605803 |
| *S. mortenseni* | 189 | FMNH 257304 | HKV 63761 | Siem Reap District, Siem Reap Province, Cambodia | 13.4350 | 103.8936 | MG607096 | MG606762 | MG606418 | MG606092 | MG605806 |
| *S. mortenseni* | 190 | FMNH 257305 | HKV 63763 | Siem Reap District, Siem Reap Province, Cambodia | 13.4350 | 103.8936 | MG607097 | MG606763 | MG606419 | MG606093 | n/a |
| *S. mortenseni* | 191 | FMNH 257306 | HKV 63765 | Siem Reap District, Siem Reap Province, Cambodia | 13.4350 | 103.8936 | MG607098 | MG606764 | MG606420 | MG606094 | MG605807 |
| *S. mortenseni* | 192 | FMNH 257307 | HKV 63722 | Bante Sre District, Siem Reap Province, Cambodia | 13.4350 | 103.8936 | MG607092 | MG606758 | MG606414 | MG606088 | n/a |
| *S. mortenseni* | 193 | FMNH 257308 | HKV 63723 | Bante Sre District, Siem Reap Province, Cambodia | 13.4350 | 103.8936 | MG607093 | MG606759 | MG606415 | MG606089 | MG605804 |
| *S. mortenseni* | 194 | FMNH 257309 | HKV 63724 | Bante Sre Distric, Siem Reap Province, Cambodia | 13.4350 | 103.8936 | MG607094 | MG606760 | MG606416 | MG606090 | MG605805 |
| *S. mortenseni* | 195 | FMNH 257310 | HKV 63725 | Bante Sre District, Siem Reap Province, Cambodia | 13.5672 | 104.1019 | MG607095 | MG606761 | MG606417 | MG606091 | n/a |
| *S. mortenseni* | 196 | FMNH 261949 | HKV 64184 | Kampot District, Kampot Province, Cambodia | 10.6314 | 104.0425 | MG607117 | MG606784 | MG606440 | MG606113 | MG605821 |
| *S. mortenseni* | 197 | FMNH 261950 | HKV 64185 | Kampot District, Kampot Province, Cambodia | 10.6314 | 104.0425 | MG607118 | MG606785 | MG606441 | MG606114 | n/a |
| *S. mortenseni* | 198 | FMNH 261951 | HKV 64186 | Kampot District, Kampot Province, Cambodia | 10.6264 | 104.0250 | MG607119 | MG606786 | MG606442 | MG606115 | MG605822 |
| *S. mortenseni* | 199 | FMNH 261952 | HKV 64474 | Kampot District, Kampot Province, Cambodia | 10.6314 | 104.0425 | MG607134 | MG606801 | MG606456 | MG606126 | n/a |
| *S. mortenseni* | 200 | FMNH 261953 | HKV 64475 | Kampot District, Kampot Province, Cambodia | 10.6314 | 104.0425 | MG607135 | MG606802 | MG606457 | MG606127 | n/a |
| *S. mortenseni* | 201 | FMNH 261954 | HKV 64476 | Kampot District, Kampot Province, Cambodia | 10.6314 | 104.0425 | MG607136 | MG606803 | MG606458 | MG606128 | n/a |
| *S. mortenseni* | 202 | FMNH 261955 | HKV 64477 | Kampot District, Kampot Province, Cambodia | 10.6314 | 104.0425 | MG607137 | MG606804 | MG606459 | MG606129 | n/a |
| *S. mortenseni* | 203 | FMNH 261956 | HKV 64504 | Kampot District, Kampot Province, Cambodia | 10.6314 | 104.0425 | MG607138 | MG606805 | MG606460 | MG606130 | MG605830 |
| *S. mortenseni* | 204 | FMNH 261957 | HKV 64505 | Kampot District, Kampot Province, Cambodia | 10.6578 | 104.0519 | MG607139 | MG606806 | MG606461 | MG606131 | MG605831 |
| *S. mortenseni* | 205 | FMNH 261958 | HKV 64510 | Kampot District, Kampot Province, Cambodia | 10.6314 | 104.0425 | MG607140 | MG606807 | MG606462 | MG606132 | n/a |
| *S. mortenseni* | 206 | FMNH 261959 | HKV 64511 | Kampot District, Kampot Province, Cambodia | 10.6219 | 104.0478 | MG607141 | MG606808 | MG606463 | MG606133 | n/a |
| *S. mortenseni* | 207 | FMNH 261960 | HKV 64512 | Kampot District, Kampot Province, Cambodia | 10.6219 | 104.0478 | MG607142 | MG606809 | MG606464 | MG606134 | n/a |
| *S. mortenseni* | 208 | FMNH 261961 | HKV 64596 | Kampot District, Kampot Province, Cambodia | **10.6314** | **104.0425** | MG607146 | MG606813 | MG606468 | n/a | n/a |
| *S. mortenseni* | 209 | FMNH 261962 | HKV 64600 | Kampot District, Kampot Province, Cambodia | **10.6314** | **104.0425** | MG607147 | MG606814 | MG606469 | MG606136 | MG605832 |
| *S. mortenseni* | 210 | FMNH 261963 | HKV 64609 | Phnom Sruoch District, Kampong Speu Province, Cambodia | 11.3667 | 104.1078 | MG607148 | MG606815 | MG606470 | MG606137 | MG605833 |
| *S. mortenseni* | 211 | FMNH 261964 | HKV 64610 | Phnom Sruoch District, Kampong Speu Province, Cambodia | 11.3667 | 104.1078 | MG607149 | MG606816 | MG606471 | MG606138 | MG605834 |
| *S. mortenseni* | 212 | FMNH 261965 | HKV 64640 | Phnom Sruoch District, Kampong Speu Province, Cambodia | 11.3667 | 104.1078 | MG607150 | MG606817 | MG606472 | MG606139 | MG605835 |
| *S. mortenseni* | 213 | FMNH 261966 | HKV 64641 | Phnom Sruoch District, Kampong Speu Province, Cambodia | 11.3108 | 104.0783 | MG607151 | MG606818 | MG606473 | MG606140 | n/a |
| *S. mortenseni* | 214 | FMNH 261967 | HKV 64642 | Phnom Sruoch District, Kampong Speu Province, Cambodia | 11.3108 | 104.0783 | MG607152 | MG606819 | MG606474 | n/a | MG605836 |
| *S. mortenseni* | 215 | FMNH 261968 | HKV 64643 | Phnom Sruoch District, Kampong Speu Province, Cambodia | 11.3667 | 104.1078 | MG607153 | MG606820 | MG606475 | MG606141 | n/a |
| *S. mortenseni* | 216 | FMNH 261969 | HKV 64683 | Phnom Sruoch District, Kampong Speu Province, Cambodia | 11.3667 | 104.1078 | MG607155 | MG606822 | MG606477 | MG606142 | n/a |
| *S. mortenseni* | 217 | FMNH 261970 | HKV 64684 | Phnom Sruoch District, Kampong Speu Province, Cambodia | 11.3108 | 104.0783 | MG607156 | MG606823 | MG606478 | MG606143 | n/a |
| *S. mortenseni* | 218 | FMNH 261980 | HKV 64564 | Kampot District, Kampot Province, Cambodia | 10.6803 | 104.1003 | MG607143 | MG606810 | MG606465 | MG606135 | n/a |
| *S. mortenseni* | 219 | FMNH 262634 | HKV 64565 | Kampot District, Kampot Province, Cambodia | 10.6803 | 104.1003 | MG607144 | MG606811 | MG606466 | MG605994 | MG605728 |
| *S. mortenseni* | 220 | FMNH 262635 | HKV 64566 | Kampot District, Kampot Province, Cambodia | 10.6803 | 104.1003 | MG607145 | MG606812 | MG606467 | MG605995 | MG605729 |
| *S. mortenseni* | 221 | FMNH 262636 | HKV 64682 | Phnom Sruoch District, Kampong Speu Province, Cambodia | 11.3667 | 104.1078 | MG607154 | MG606821 | MG606476 | MG605996 | n/a |
| *S. mortenseni* | 222 | FMNH 263299 | HKV 33049 | Thmar Biang District, Koh Kong Province, Cambodia | 11.9526 | 103.2908 | MG607023 | MG606689 | MG606343 | MG606006 | n/a |
| *S. mortenseni* | 223 | FMNH 263318 | HKV 33110 | Thmar Biang District, Koh Kong Province, Cambodia | 11.6756 | 103.7252 | MG607024 | MG606690 | MG606344 | MG606007 | MG605737 |
| *S. mortenseni* | 224 | FMNH 266290 | HKV 66296 | Boong Klar District, Nong Khai Province, Thailand | 18.2419 | 103.9624 | MG607199 | MG606866 | MG606521 | MG606185 | MG605870 |
| *S. mortenseni* | 225 | FMNH 266293 | HKV 66332 | Boong Klar District, Nong Khai Province, Thailand | 18.2320 | 103.9565 | MG607200 | MG606867 | MG606522 | MG606186 | MG605871 |
| *S. mortenseni* | 226 | FMNH 266295 | HKV 66334 | Boong Klar District, Nong Khai Province, Thailand | 18.2320 | 103.9565 | MG607201 | MG606868 | MG606523 | MG606187 | MG605872 |
| *S. mortenseni* | 227 | FMNH 266296 | HKV 66335 | Boong Klar District, Nong Khai Province, Thailand | 18.2320 | 103.9565 | MG607202 | MG606869 | MG606524 | MG606188 | n/a |
| *S. mortenseni* | 228 | FMNH 266299 | HKV 66338 | Boong Klar District, Nong Khai Province, Thailand | 18.2320 | 103.9565 | MG607203 | MG606870 | MG606525 | MG606189 | MG605873 |
| *S. mortenseni* | 229 | FMNH 266301 | HKV 66340 | Boong Klar District, Nong Khai Province, Thailand | 18.2320 | 103.9565 | MG607204 | MG606871 | MG606526 | MG606190 | MG605874 |
| *S. mortenseni* | 230 | FMNH 266303 | HKV 66342 | Boong Klar District, Nong Khai Province, Thailand | 18.2320 | 103.9565 | MG607205 | MG606872 | MG606527 | MG606191 | MG605875 |
| *S. mortenseni* | 231 | FMNH 266305 | HKV 66344 | Boong Klar District, Nong Khai Province, Thailand | 18.2643 | 103.9061 | MG607206 | MG606873 | MG606528 | MG606192 | MG605876 |
| *S. mortenseni* | 232 | FMNH 266306 | HKV 66345 | Boong Klar District, Nong Khai Province, Thailand | 18.2643 | 103.9061 | MG607207 | MG606874 | MG606529 | MG606193 | n/a |
| *S. mortenseni* | 233 | FMNH 266308 | HKV 66414 | Na Chaloey District, Ubon Ratchathani Province, Thailand | 14.4348 | 105.2536 | MG607208 | MG606875 | MG606530 | MG606194 | MG605877 |
| *S. mortenseni* | 234 | FMNH 266309 | HKV 66448 | Na Chaloey District, Ubon Ratchathani Province, Thailand | 14.4424 | 105.2731 | MG607209 | MG606876 | MG606531 | MG606195 | MG605878 |
| *S. mortenseni* | 235 | FMNH 266310 | HKV 66449 | Na Chaloey District, Ubon Ratchathani Province, Thailand | 14.4424 | 105.2731 | MG607210 | MG606877 | MG606532 | MG606196 | n/a |
| *S. mortenseni* | 236 | FMNH 266311 | HKV 66450 | Na Chaloey District, Ubon Ratchathani Province, Thailand | 14.4424 | 105.2731 | MG607211 | MG606878 | MG606533 | MG606197 | n/a |
| *S. mortenseni* | 237 | FMNH 266312 | HKV 66451 | Na Chaloey District, Ubon Ratchathani Province, Thailand | 14.4424 | 105.2731 | MG607212 | MG606879 | MG606534 | MG606198 | MG605879 |
| *S. mortenseni* | 238 | FMNH 266314 | HKV 66453 | Na Chaloey District, Ubon Ratchathani Province, Thailand | 14.4424 | 105.2731 | MG607213 | MG606880 | MG606535 | MG606199 | n/a |
| *S. mortenseni* | 239 | FMNH 266316 | HKV 66465 | Na Chaloey District, Ubon Ratchathani Province, Thailand | 14.4326 | 105.2567 | MG607214 | MG606881 | MG606536 | MG606200 | n/a |
| *S. mortenseni* | 240 | FMNH 266317 | HKV 66466 | Nam Yuen District, Ubon Ratchathani Province, Thailand | 14.4353 | 105.1049 | MG607215 | MG606882 | MG606537 | MG606201 | n/a |
| *S. mortenseni* | 241 | FMNH 266319 | HKV 66480 | Na Chaloey District, Ubon Ratchathani Province, Thailand | 14.4378 | 105.2801 | MG607216 | MG606883 | MG606538 | MG606202 | n/a |
| *S. mortenseni* | 242 | FMNH 266320 | HKV 66509 | Boontharik District, Ubon Ratchatani Province, Thailand | 14.4419 | 105.3075 | MG607217 | MG606884 | MG606539 | MG606203 | MG605880 |
| *S. mortenseni* | 243 | FMNH 266321 | HKV 66512 | Sa Kaeo District, Sa Kaeo Province, Thailand | 14.0399 | 102.2657 | MG607218 | MG606885 | MG606540 | MG606204 | n/a |
| *S. mortenseni* | 244 | FMNH 266323 | HKV 66514 | Sa Kaeo District, Sa Kaeo Province, Thailand | 14.0399 | 102.2657 | MG607219 | MG606886 | MG606541 | MG606205 | n/a |
| *S. mortenseni* | 245 | FMNH 266324 | HKV 66515 | Sa Kaeo District, Sa Kaeo Province, Thailand | 14.0399 | 102.2657 | MG607220 | MG606887 | MG606542 | MG606206 | MG605881 |
| *S. mortenseni* | 246 | FMNH 266326 | HKV 66563 | Sa Kaeo District, Sa Kaeo Province, Thailand | 14.1277 | 102.2592 | MG607221 | MG606888 | MG606543 | MG606207 | MG605882 |
| *S. mortenseni* | 247 | FMNH 266328 | HKV 66565 | Sa Kaeo District, Sa Kaeo Province, Thailand | 14.1277 | 102.2592 | MG607222 | MG606889 | MG606544 | MG606208 | MG605883 |
| *S. mortenseni* | 248 | FMNH 266329 | HKV 66572 | Sa Kaeo District, Sa Kaeo Province, Thailand | 13.9953 | 102.2065 | MG607223 | MG606890 | MG606545 | MG606209 | MG605884 |
| *S. mortenseni* | 249 | FMNH 266330 | HKV 66573 | Sa Kaeo District, Sa Kaeo Province, Thailand | 13.9953 | 102.2065 | MG607224 | MG606891 | MG606546 | MG606210 | MG605885 |
| *S. mortenseni* | 250 | FMNH 266331 | HKV 66576 | Sa Kaeo District, Sa Kaeo Province, Thailand | 13.9953 | 102.2065 | MG607225 | MG606892 | MG606547 | MG606211 | n/a |
| *S. mortenseni* | 251 | FMNH 266333 | HKV 66578 | Sa Kaeo District, Sa Kaeo Province, Thailand | 13.9953 | 102.2065 | MG607226 | MG606893 | MG606548 | MG606212 | MG605886 |
| *S. mortenseni* | 252 | FMNH 266334 | HKV 66600 | Sa Kaeo District, Sa Kaeo Province, Thailand | 14.0933 | 102.3078 | MG607227 | MG606894 | MG606549 | MG606213 | MG605887 |
| *S. mortenseni* | 253 | KU 328164 | DSM 1142 | Wang Nam Kaeo District, Nakhon Ratchasima Province, Thailand | 14.5000 | 101.9200 | MG607275 | MG606942 | MG606598 | MG606264 | MG605931 |
| *S. mortenseni* | 254 | KU 328166 | DSM 1309 | Wang Nam Kaeo District, Nakhon Ratchasima Province, Thailand | 14.5000 | 101.9200 | MG607276 | MG606943 | MG606599 | MG606265 | MG605932 |
| *S. mortenseni* | 255 | KU 328167 | DSM 1376 | Wang Nam Kaeo District, Nakhon Ratchasima Province, Thailand | 14.5000 | 101.9200 | MG607277 | MG606944 | MG606600 | MG606266 | MG605933 |
| *S. mortenseni* | 256 | NCSM 80910 | BLS 15865 | Viengthong District, Bolikhamxay Province, Laos | 18.4182 | 104.4012 | MG607309 | MG606965 | MG606632 | MG606298 | MG605965 |
| *S. mortenseni* | 257 | NCSM 80911 | BLS 15867 | Viengthong District, Bolikhamxay Province, Laos | 18.4182 | 104.4012 | MG607310 | MG606966 | MG606633 | MG606299 | MG605966 |
| *S. mortenseni* | 258 | NCSM 80912 | BLS 15870 | Viengthong District, Bolikhamxay Province, Laos | 18.4259 | 104.3926 | MG607311 | MG606967 | MG606634 | MG606300 | MG605967 |
| *S. mortenseni* | 259 | NCSM 80913 | BLS 15871 | Viengthong District, Bolikhamxay Province, Laos | 18.4259 | 104.3926 | MG607312 | MG606968 | MG606635 | MG606301 | MG605968 |
| *S. mortenseni* | 260 | NCSM 80914 | BLS 15872 | Viengthong District, Bolikhamxay Province, Laos | 18.4259 | 104.3926 | MG607313 | MG606969 | MG606636 | MG606302 | MG605969 |
| *S. mortenseni* | 261 | NCSM 80915 | BLS 15874 | Viengthong District, Bolikhamxay Province, Laos | 18.4259 | 104.3926 | MG607314 | MG606970 | MG606637 | MG606303 | MG605970 |
| *S. mortenseni* | 262 | NCSM 80916 | BLS 15950 | Viengthong District, Bolikhamxay Province, Laos | 18.4057 | 104.4215 | MG607315 | MG606971 | MG606638 | MG606304 | MG605971 |
| *S. nigrovittata* | 263 | CAS 230598 | JBS 13640 | Taunggyi District, Shan State, Myanmar | 21.0402 | 96.3904 | MG607262 | MG606929 | MG606585 | MG606251 | MG605918 |
| *S. nigrovittata* | 264 | CAS 232371 | JBS 16892 | Myitkyina District, Kachin State, Myanmar | 26.6865 | 96.2367 | MG607266 | MG606933 | MG606589 | MG606255 | MG605922 |
| *S. nigrovittata* | 265 | CAS 245392 | CAS-MHS-28860 | Khandi District, Sagaing State, Myanmar | 26.1264 | 95.5399 | MG607271 | MG606938 | MG606594 | MG606260 | MG605927 |
| *S. nigrovittata* | 266 | FMNH 255429 | HKV 63341 | Viengthong District, Huaphahn Province, Laos | 20.2333 | 103.2667 | MG607081 | MG606744 | MG606400 | MG606074 | MG605795 |
| *S. nigrovittata* | 267 | FMNH 255430 | HKV 63342 | Viengthong District, Huaphahn Province, Laos | 20.2333 | 103.2667 | MG607082 | MG606745 | MG606401 | MG606075 | MG605796 |
| *S. nigrovittata* | 268 | FMNH 255431 | HKV 63343 | Viengthong District, Huaphahn Province, Laos | 20.2333 | 103.2667 | MG607083 | MG606746 | MG606402 | MG606076 | n/a |
| *S. nigrovittata* | 269 | FMNH 255432 | HKV 63344 | Viengthong District, Huaphahn Province, Laos | 20.2333 | 103.2667 | MG607084 | MG606747 | MG606403 | MG606077 | n/a |
| *S. nigrovittata* | 270 | FMNH 255433 | HKV 63345 | Viengthong District, Huaphahn Province, Laos | 20.2333 | 103.2667 | MG607085 | MG606748 | MG606404 | MG606078 | n/a |
| *S. nigrovittata* | 271 | FMNH 258122 | HKV 64208 | Phongsaly District, Phongsaly Province, Laos | 22.0939 | 102.2139 | MG607120 | MG606787 | MG606443 | MG606116 | MG605823 |
| *S. nigrovittata* | 272 | FMNH 258123 | HKV 64209 | Phongsaly District, Phongsaly Province, Laos | 20.0939 | 102.2139 | MG607121 | MG606788 | MG606444 | MG606117 | MG605824 |
| *S. nigrovittata* | 273 | FMNH 258124 | HKV 64210 | Phongsaly District, Phongsaly Province, Laos | 22.0939 | 102.2139 | MG607122 | MG606789 | MG606445 | MG606118 | n/a |
| *S. nigrovittata* | 274 | FMNH 258125 | HKV 64224 | Phongsaly District, Phongsaly Province, Laos | 22.0939 | 102.2139 | MG607124 | MG606791 | MG606447 | MG606120 | MG605825 |
| *S. nigrovittata* | 275 | FMNH 258126 | HKV 64257 | Phongsaly District, Phongsaly Province, Laos | 22.0964 | 102.2189 | MG607127 | MG606794 | MG606450 | MG606122 | n/a |
| *S. nigrovittata* | 276 | FMNH 258127 | HKV 64279 | Phongsaly District, Phongsaly Province, Laos | 22.1511 | 102.2053 | MG607128 | MG606795 | MG606451 | MG606123 | MG605827 |
| *S. nigrovittata* | 277 | FMNH 258128 | HKV 64323 | Phongsaly District, Phongsaly Province, Laos | 22.0919 | 102.1053 | MG607130 | MG606797 | MG606453 | MG606125 | MG605829 |
| *S. nigrovittata* | 278 | FMNH 258204 | HKV 64231 | Phongsaly District, Phongsaly Province, Laos | 22.0939 | 102.2139 | MG607126 | MG606793 | MG606449 | n/a | n/a |
| *S. nigrovittata* | 279 | FMNH 258267 | HKV 64127 | Phu Phiang District, Xaignabouli Province, Laos | 18.9022 | 101.5750 | MG607112 | MG606778 | MG606434 | MG606108 | MG605816 |
| *S. nigrovittata* | 280 | FMNH 258268 | HKV 64222 | Phongsaly District, Phongsaly Province, Laos | 20.0939 | 102.2139 | MG607123 | MG606790 | MG606446 | MG606119 | n/a |
| *S. nigrovittata* | 281 | FMNH 258329 | HKV 64225 | Phongsaly District, Phongsaly Province, Laos | 22.0939 | 102.2139 | MG607125 | MG606792 | MG606448 | MG606121 | MG605826 |
| *S. nigrovittata* | 282 | FMNH 258330 | HKV 64318 | Phongsaly District, Phongsaly Province, Laos | 22.1608 | 102.1853 | MG607129 | MG606796 | MG606452 | MG606124 | n/a |
| *S. nigrovittata* | 283 | FMNH 258533 | HKV 64132 | Phu Phiang District, Xaignabouli Province, Laos | 18.9022 | 101.5750 | MG607113 | MG606779 | MG606435 | MG606109 | MG605817 |
| *S. nigrovittata* | 284 | FMNH 258534 | HKV 64152 | Phu Phiang District, Xaignabouli Province, Laos | 18.9022 | 101.5750 | MG607114 | MG606780 | MG606436 | n/a | n/a |
| *S. nigrovittata* | 285 | FMNH 258535 | HKV 64153 | Phu Phiang District, Xaignabouli Province, Laos | 18.9022 | 101.5750 | MG607115 | MG606781 | MG606437 | MG606110 | MG605818 |
| *S. nigrovittata* | 286 | FMNH 258536 | HKV 64154 | Phu Phiang District, Xaignabouli Province, Laos | 18.9022 | 101.5750 | MG607116 | MG606782 | MG606438 | MG606111 | MG605819 |
| *S. nigrovittata* | 287 | FMNH 258537 | HKV 64155 | Phu Phiang District, Xaignabouli Province, Laos | 18.9022 | 101.5750 | MG607352 | MG606783 | MG606439 | MG606112 | MG605820 |
| *S. nigrovittata* | 288 | FMNH 263444 | THNSM 4450 | Hua Hin District, Prachuap Kirikhan Province, Thailand | 12.5378 | 99.4614 | MG607316 | MG606972 | MG606639 | MG606305 | n/a |
| *S. nigrovittata* | 289 | FMNH 263445 | THNSM 4464 | Hua Hin District, Prachuap Kirikhan Province, Thailand | 12.5378 | 99.4614 | MG607317 | MG606973 | MG606640 | MG606306 | n/a |
| *S. nigrovittata* | 290 | FMNH 263446 | THNSM 4477 | Hua Hin District, Prachuap Kirikhan Province, Thailand | 12.5378 | 99.4614 | MG607318 | MG606974 | MG606641 | MG606307 | n/a |
| *S. nigrovittata* | 291 | FMNH 263447 | THNSM 4496 | Hua Hin District, Prachuap Kirikhan Province, Thailand | 12.5378 | 99.4614 | MG607319 | MG606975 | MG606642 | MG606308 | n/a |
| *S. nigrovittata* | 292 | FMNH 263448 | THNSM 4497 | Hua Hin District, Prachuap Kirikhan Province, Thailand | 12.5378 | 99.4614 | MG607320 | MG606976 | MG606643 | MG606309 | MG605972 |
| *S. nigrovittata* | 293 | FMNH 266280 | HKV 65998 | Phu Rua District, Loei Province, Thailand | 17.3341 | 101.5090 | MG607192 | MG606859 | MG606514 | MG606178 | MG605863 |
| *S. nigrovittata* | 294 | FMNH 266281 | HKV 66211 | Phu Rua District, Loei Province, Thailand | 17.2802 | 101.5188 | MG607193 | MG606860 | MG606515 | MG606179 | MG605864 |
| *S. nigrovittata* | 295 | FMNH 266283 | HKV 66225 | Phu Rua District, Loei Province, Thailand | 17.2590 | 101.5063 | MG607194 | MG606861 | MG606516 | MG606180 | MG605865 |
| *S. nigrovittata* | 296 | FMNH 266284 | HKV 66226 | Phu Rua District, Loei Province, Thailand | 17.2590 | 101.5063 | MG607195 | MG606862 | MG606517 | MG606181 | MG605866 |
| *S. nigrovittata* | 297 | FMNH 266285 | HKV 66251 | Phu Rua District, Loei Province, Thailand | 17.3452 | 101.5078 | MG607196 | MG606863 | MG606518 | MG606182 | MG605867 |
| *S. nigrovittata* | 298 | FMNH 266287 | HKV 66281 | Phu Rua District, Loei Province, Thailand | 17.3515 | 101.5037 | MG607197 | MG606864 | MG606519 | MG606183 | MG605868 |
| *S. nigrovittata* | 299 | FMNH 266288 | HKV 66282 | Phu Rua District, Loei Province, Thailand | 17.3515 | 101.5037 | MG607198 | MG606865 | MG606520 | MG606184 | MG605869 |
| *S. nigrovittata* | 300 | FMNH 268374 | HKV 66620 | Bang Saphan District, Prachuap Kirikhan Province, Thailand | **11.5026** | **99.4730** | MG607228 | MG606895 | MG606550 | MG606214 | n/a |
| *S. nigrovittata* | 301 | FMNH 268375 | HKV 66628 | Bang Saphan District, Prachuap Kirikhan Province, Thailand | **11.5026** | **99.4730** | MG607229 | MG606896 | MG606551 | MG606215 | MG605888 |
| *S. nigrovittata* | 302 | FMNH 268376 | HKV 66632 | Bang Saphan District, Prachuap Kirikhan Province, Thailand | **11.5026** | **99.4730** | MG607230 | MG606897 | MG606552 | MG606216 | MG605889 |
| *S. nigrovittata* | 303 | FMNH 268377 | HKV 66633 | Bang Saphan District, Prachuap Kirikhan Province, Thailand | **11.5026** | **99.4730** | MG607231 | MG606898 | MG606553 | MG606217 | MG605890 |
| *S. nigrovittata* | 304 | FMNH 268378 | HKV 66634 | Bang Saphan District, Prachuap Kirikhan Province, Thailand | **11.5026** | **99.4730** | MG607232 | MG606899 | MG606554 | MG606218 | MG605891 |
| *S. nigrovittata* | 305 | FMNH 268379 | HKV 66636 | Bang Saphan District, Prachuap Kirikhan Province, Thailand | **11.5026** | **99.4730** | MG607233 | MG606900 | MG606555 | MG606219 | MG605892 |
| *S. nigrovittata* | 306 | FMNH 268380 | HKV 66650 | Bang Saphan District, Prachuap Kirikhan Province, Thailand | **11.5026** | **99.4730** | MG607234 | n/a | MG606556 | MG606220 | MG605893 |
| *S. nigrovittata* | 307 | FMNH 268381 | HKV 66655 | Bang Saphan District, Prachuap Kirikhan Province, Thailand | **11.5026** | **99.4730** | MG607235 | MG606901 | MG606557 | MG606221 | MG605894 |
| *S. nigrovittata* | 308 | FMNH 268382 | HKV 66656 | Bang Saphan District, Prachuap Kirikhan Province, Thailand | **11.5026** | **99.4730** | MG607236 | MG606902 | MG606558 | MG606222 | MG605895 |
| *S. nigrovittata* | 309 | FMNH 268383 | HKV 66657 | Bang Saphan District, Prachuap Kirikhan Province, Thailand | **11.5026** | **99.4730** | MG607237 | MG606903 | MG606559 | MG606223 | MG605896 |
| *S. nigrovittata* | 310 | FMNH 268758 | RFI 50804 | Lang Saka District, Nakhon Si Thammarat Province, Thailand | **8.4947** | **99.7406** | MG607025 | MG606691 | MG606345 | MG606009 | MG605739 |
| *S. nigrovittata* | 311 | FMNH 268759 | RFI 50820 | Lang Saka District, Nakhon Si Thammarat Province, Thailand | **8.3700** | **99.7025** | MG607027 | MG606693 | MG606347 | MG606011 | MG605741 |
| *S. nigrovittata* | 312 | FMNH 268760 | RFI 50824 | Lang Saka District, Nakhon Si Thammarat Province, Thailand | **8.3700** | **99.7025** | MG607028 | MG606694 | MG606348 | MG606012 | MG605742 |
| *S. nigrovittata* | 313 | FMNH 268761 | RFI 50829 | Lang Saka District, Nakhon Si Thammarat Province, Thailand | **8.4947** | **99.7406** | MG607030 | MG606696 | MG606350 | MG606014 | MG605744 |
| *S. nigrovittata* | 314 | FMNH 268762 | RFI 50856 | Lang Saka District, Nakhon Si Thammarat Province, Thailand | **8.4947** | **99.7406** | MG607031 | MG606697 | MG606351 | MG606015 | MG605745 |
| *S. nigrovittata* | 315 | FMNH 268963 | RFI 50817 | Lang Saka District, Nakhon Si Thammarat Province, Thailand | **8.4947** | **99.7406** | MG607026 | MG606692 | MG606346 | MG606010 | MG605740 |
| *S. nigrovittata* | 316 | FMNH 268964 | RFI 50825 | Lang Saka District, Nakhon Si Thammarat Province, Thailand | **8.3700** | **99.7025** | MG607029 | MG606695 | MG606349 | MG606013 | MG605743 |
| *S. nigrovittata* | 317 | FMNH 268965 | RFI 50868 | Lang Saka District, Nakhon Si Thammarat Province, Thailand | **8.4947** | **99.7406** | MG607032 | MG606698 | MG606352 | MG606016 | MG605746 |
| *S. nigrovittata* | 318 | FMNH 268966 | RFI 50872 | Lang Saka District, Nakhon Si Thammarat Province, Thailand | **8.4947** | **99.7406** | MG607033 | MG606699 | MG606353 | MG606017 | MG605747 |
| *S. nigrovittata* | 319 | FMNH 268967 | RFI 50874 | Lang Saka District, Nakhon Si Thammarat Province, Thailand | **8.3700** | **99.7025** | MG607034 | MG606700 | MG606354 | MG606018 | MG605748 |
| *S. nigrovittata* | 320 | FMNH 268968 | RFI 50875 | Lang Saka District, Nakhon Si Thammarat Province, Thailand | **8.3700** | **99.7025** | MG607035 | MG606701 | MG606355 | MG606019 | MG605749 |
| *S. nigrovittata* | 321 | FMNH 271354 | BLS 10517 | Vieng Phou Kha District, Luang Namtha Province, Laos | 20.8689 | 101.0553 | MG607013 | MG606680 | MG606333 | MG605999 | MG605732 |
| *S. nigrovittata* | 322 | FMNH 271358 | BLS 10602 | Vieng Phou Kha District, Luang Namtha Province, Laos | 20.8689 | 101.0553 | MG607015 | MG606681 | MG606335 | MG606000 | MG605733 |
| *S. nigrovittata* | 323 | FMNH 271373 | BLS 10516 | Vieng Phou Kha District, Luang Namtha Province, Laos | 20.8689 | 101.0553 | MG607012 | MG606679 | MG606332 | MG605998 | MG605731 |
| *S. nigrovittata* | 324 | FMNH 271375 | BLS 10601 | Vieng Phou Kha District, Luang Namtha Province, Laos | 20.8689 | 101.0553 | MG607014 | n/a | MG606334 | n/a | n/a |
| *S. nigrovittata* | 325 | FMNH 271409 | BLS 10515 | Vieng Phou Kha District, Luang Namtha Province, Laos | 20.8689 | 101.0553 | MG607011 | MG606678 | MG606331 | MG605997 | MG605730 |
| *S. nigrovittata* | 326 | KU 331596 | CWL 748 | Dien Bien Province, Vietnam | 22.3866 | 102.2395 | MG607278 | MG606945 | MG606601 | MG606267 | MG605934 |
| *S. nigrovittata* | 327 | KU 331600 | CWL 803 | Dien Bien Province, Vietnam | 22.1667 | 102.5667 | MG607279 | MG606946 | MG606602 | MG606268 | MG605935 |
| *S. nigrovittata* | 328 | KU 331603 | CWL 825 | Dien Bien Province, Vietnam | 22.1667 | 102.5667 | MG607280 | MG606947 | MG606603 | MG606269 | MG605936 |
| *S. nigrovittata* | 329 | KU 331610 | CWL 904 | Dien Bien Province, Vietnam | 22.1667 | 102.5667 | MG607281 | MG606948 | MG606604 | MG606270 | MG605937 |
| *S. nigrovittata* | 330 | NCSM 79393 | BLS 15051 | Luang Phabang District, Luang Phabang Province, Laos | 19.7491 | 101.9917 | MG607298 | MG606954 | MG606621 | MG606287 | MG605954 |
| *S. nigrovittata* | 331 | NCSM 79394 | BLS 15086 | Luang Phabang District, Luang Phabang Province, Laos | 19.7491 | 101.9917 | MG607299 | MG606955 | MG606622 | MG606288 | MG605955 |
| *S. nigrovittata* | 332 | NCSM 79396 | BLS 15088 | Luang Phabang District, Luang Phabang Province, Laos | 19.7491 | 101.9917 | MG607300 | MG606956 | MG606623 | MG606289 | MG605956 |
| *S. nigrovittata* | 333 | NCSM 79406 | BLS 15207 | Xaignabouli District, Xaignabouli Province, Laos | 19.0304 | 101.7623 | MG607301 | MG606957 | MG606624 | MG606290 | MG605957 |
| *S. nigrovittata* | 334 | NCSM 79409 | BLS 15261 | Paklay District, Xaignabouli Province, Laos | 18.8259 | 101.8409 | MG607302 | MG606958 | MG606625 | MG606291 | MG605958 |
| *S. nigrovittata* | 335 | NCSM 79411 | BLS 15328 | Paklay District, Xaignabouli Province, Laos | 18.5139 | 101.6590 | MG607303 | MG606959 | MG606626 | MG606292 | MG605959 |
| *S. nigrovittata* | 336 | NCSM 79921 | BLS 14648 | Xaysomboun District, Vientiane Province, Laos | 18.9655 | 102.8486 | MG607304 | MG606960 | MG606627 | MG606293 | MG605960 |
| *S. nigrovittata* | 337 | NCSM 79924 | BLS 15413 | Xaysomboun District, Vientiane Province, Laos | 19.0005 | 102.8945 | MG607305 | MG606961 | MG606628 | MG606294 | MG605961 |
| *S. nigrovittata* | 338 | NCSM 79926 | BLS 15497 | Xaysomboun District, Vientiane Province, Laos | 19.0899 | 102.8960 | MG607306 | MG606962 | MG606629 | MG606295 | MG605962 |
| *S. nigrovittata* | 339 | NCSM 79928 | BLS 15528 | Xaysomboun District, Vientiane Province, Laos | 19.0909 | 102.8935 | MG607307 | MG606963 | MG606630 | MG606296 | MG605963 |
| *S. nigrovittata* | 340 | NCSM 79931 | BLS 15553 | Xaysomboun District, Vientiane Province, Laos | 19.0899 | 102.8960 | MG607308 | MG606964 | MG606631 | MG606297 | MG605964 |
| *S. nigrovittata* | 341 | NCSM 80642 | BLS 14663 | Boun Tai District, Phongsaly Province, Laos | 21.3366 | 101.8732 | MG607255 | MG606921 | MG606577 | MG606243 | MG605911 |
| *S. nigrovittata* | 342 | NCSM 80644 | BLS 14683 | Boun Tai District, Phongsaly Province, Laos | 21.3329 | 101.8821 | MG607256 | MG606922 | MG606578 | MG606244 | MG605912 |
| *S. nigrovittata* | 343 | NCSM 80645 | BLS 14684 | Boun Tai District, Phongsaly Province, Laos | 21.3329 | 101.8821 | MG607257 | MG606923 | MG606579 | MG606245 | MG605913 |
| *S. nigrovittata* | 344 | NCSM 80654 | BLS 15690 | Boun Tai District, Phongsaly Province, Laos | 21.3344 | 101.8414 | MG607259 | MG606925 | MG606581 | MG606247 | MG605915 |
| *S. nigrovittata* | 345 | NCSM 80658 | BLS 15720 | Boun Tai District, Phongsaly Province, Laos | 21.3292 | 101.8773 | MG607260 | MG606926 | MG606582 | MG606248 | MG605916 |
| *S. nigrovittata* | 346 | NUOL 00006 | BLS 15674 | Boun Tai District, Phongsaly Province, Laos | 21.3274 | 101.8894 | MG607258 | MG606924 | MG606580 | MG606246 | MG605914 |
| *S. nigrovittata* | 347 | THNHM 1050 | THNSM 4701 | Lang Saka District, Nakhon Si Thammarat Province, Thailand | **8.3689** | **99.7347** | MG607321 | MG606977 | MG606644 | MG606310 | MG605973 |
| *S. nigrovittata* | 348 | THNHM uncataloged | HKV 65806 | Bang Saphan District, Prachuap Kirikhan Province, Thailand | 12.7961 | 99.4561 | MG607179 | MG606846 | MG606501 | n/a | MG605854 |
| *S. roberti* sp. nov. | 349 | CAS 229665 | JBS 15681 | Dewei District, Tanitharyi State, Myanmar | 13.8613 | 98.2883 | n/a | MG606927 | MG606583 | MG606249 | n/a |
| *S. roberti* sp. nov. | 350 | CAS 229796 | JBS 15960 | Dewei District, Tanitharyi State, Myanmar | 13.8453 | 98.4585 | MG607261 | MG606928 | MG606584 | MG606250 | MG605917 |
| *S. roberti* sp. nov. | 351 | CAS 243913 | CAS-MHS-28562 | Dewei District, Tanitharyi State, Myanmar | 14.7475 | 98.2213 | MG607270 | MG606937 | MG606593 | MG606259 | MG605926 |
