# Supplementary material for: Hidden species diversity in Sylvirana nigrovittata (Amphibia: Ranidae) highlights the importance of taxonomic revisions in biodiversity conservation
Source: PLoS One. 2018 Mar 14;13(3):e0192766. doi: 10.1371/journal.pone.0192766 (PMC5851555; doi:10.1371/journal.pone.0192766)
Supplement: S2 Table — (DOCX) [file pone.0192766.s002.docx]

**S2 Table. Samples of *Sylvirana* used in morphological analyses.** Bolded latitude and longitude indicate estimates by the authors based on stated collection localities. All other coordinates provided by respective museum.

| **Species** | **Voucher** | **Locality** | **Latitude** | **Longitude** |  |
| --- | --- | --- | --- | --- | --- |
| *annamitica* sp. nov. | BMNH 1928.6.29.7 | Khammouan, Laos | **18.3016** | **105.0879** |  |
| *annamitica* sp. nov. | BMNH 1928.6.29.7 | Khammouan, Laos | **18.3016** | **105.0879** |  |
| *annamitica* sp. nov. | FMNH 256533 | Khammouan, Laos | 17.9500 | 105.5667 |  |
| *annamitica* sp. nov. | FMNH 256535 | Khammouan, Laos | 17.9500 | 105.5667 |  |
| *annamitica* sp. nov. | FMNH 256538 | Khammouan, Laos | 17.9500 | 105.5667 |  |
| *annamitica* sp. nov. | FMNH 256540 | Khammouan, Laos | 17.9500 | 105.5667 |  |
| *annamitica* sp. nov. | AMNH A-161285 | Ha Tinh, Vietnam | **18.0667** | **106.0333** |  |
| *annamitica* sp. nov. | AMNH A-161286 | Ha Tinh, Vietnam | **18.0667** | **106.0333** |  |
| *annamitica* sp. nov. | AMNH A-161288 | Ha Tinh, Vietnam | **18.0667** | **106.0333** |  |
| *annamitica* sp. nov. | AMNH A-161290 | Ha Tinh, Vietnam | **18.0667** | **106.0333** |  |
| *annamitica* sp. nov. | AMNH A-161295 | Ha Tinh, Vietnam | 18.3647 | 105.2203 |  |
| *annamitica* sp. nov. | AMNH A-161297 | Ha Tinh, Vietnam | 18.3481 | 105.2439 |  |
| *annamitica* sp. nov. | FMNH 255629 | Nghe An, Vietnam | 18.9333 | 104.7500 |  |
| *annamitica* sp. nov. | AMNH A-181997 | Quang Nam, Vietnam | 15.3439 | 107.7358 |  |
| *annamitica* sp. nov. | NCSM 79174 | Quang Nam, Vietnam | 15.6591 | 107.6015 |  |
| *annamitica* sp. nov. | AMNH A-169308 | Thua Tien Hue, Vietnam | 16.2606 | 107.4439 |  |
| *annamitica* sp. nov. | FMNH 256539 | Khammouan, Laos | 17.9667 | 105.5667 |  |
| *annamitica* sp. nov. | AMNH A-161280 | Ha Tinh, Vietnam | 18.0667 | 106.0333 |  |
| *annamitica* sp. nov. | AMNH A-161292 | Ha Tinh, Vietnam | 18.3667 | 105.2167 |  |
| *annamitica* sp. nov. | AMNH A-161461 | Bac Kan, Vietnam | 22.4003 | 105.6317 |  |
| *annamitica* sp. nov. | ROM 27786 | Vinh Phu, Vietnam | 21.4542 | 105.6414 |  |
| *lacrima* sp. nov. | CAS 234899 | Chin, Myanmar | 20.9786 | 93.9677 |  |
| *lacrima* sp. nov. | CAS 234923 | Chin, Myanmar | 21.3868 | 94.0567 |  |
| *lacrima* sp. nov. | CAS 234925 | Chin, Myanmar | 21.3868 | 94.0567 |  |
| *lacrima* sp. nov. | CAS 234926 | Chin, Myanmar | 21.3868 | 94.0567 |  |
| *lacrima* sp. nov. | CAS 234952 | Chin, Myanmar | 21.6014 | 94.0532 |  |
| *lacrima* sp. nov. | CAS 234990 | Chin, Myanmar | 21.7807 | 93.7482 |  |
| *lacrima* sp. nov. | CAS 234993 | Chin, Myanmar | 21.7807 | 93.7482 |  |
| *lacrima* sp. nov. | CAS 234994 | Chin, Myanmar | 21.7807 | 93.7482 |  |
| *lacrima* sp. nov. | CAS 235078 | Chin, Myanmar | 21.6124 | 93.8948 |  |
| *lacrima* sp. nov. | CAS 235096 | Chin, Myanmar | 21.5953 | 93.9363 |  |
| *lacrima* sp. nov. | CAS 235165 | Chin, Myanmar | 21.2715 | 93.7523 |  |
| *lacrima* sp. nov. | CAS 235166 | Chin, Myanmar | 21.2715 | 93.7523 |  |
| *malayana* sp. nov. | CAS 247472 | Tanitharyi, Myanmar | 10.3720 | 98.6086 |  |
| *malayana* sp. nov. | CAS 247751 | Tanitharyi, Myanmar | 10.3713 | 98.6270 |  |
| *malayana* sp. nov. | CAS 247856 | Tanitharyi, Myanmar | 10.3698 | 98.6313 |  |
| *malayana* sp. nov. | CAS 247866 | Tanitharyi, Myanmar | 10.3794 | 98.6092 |  |
| *malayana* sp. nov. | BMNH 1937.3.4.1 | Pahang, Malaysia | **3.7800** | **102.7297** |  |
| *malayana* sp. nov. | BMNH 1974.3566 | Kedah, Malaysia | **6.3759** | **100.5220** |  |
| *malayana* sp. nov. | BMNH 1974.3567 | Kedah, Malaysia | **6.3759** | **100.5220** |  |
| *malayana* sp. nov. | BMNH 1974.3569 | Kedah, Malaysia | **6.3759** | **100.5220** |  |
| *malayana* sp. nov. | BMNH 1974.3570 | Kedah, Malaysia | **6.3759** | **100.5220** |  |
| *malayana* sp. nov. | BMNH 1974.3572 | Kedah, Malaysia | **6.3759** | **100.5220** |  |
| *malayana* sp. nov. | BMNH 1974.3574 | Kedah, Malaysia | **6.3759** | **100.5220** |  |
| *malayana* sp. nov. | BMNH 1974.3576 | Kedah, Malaysia | **6.3759** | **100.5220** |  |
| *malayana* sp. nov. | BMNH 1974.3578 | Kedah, Malaysia | **6.3759** | **100.5220** |  |
| *malayana* sp. nov. | LSUHC 5854 | Perak, Malaysia | 5.4910 | 101.6042 |  |
| *malayana* sp. nov. | LSUHC 5855 | Perak, Malaysia | 5.4910 | 101.6042 |  |
| *malayana* sp. nov. | LSUHC 5856 | Perak, Malaysia | 5.4910 | 101.6042 |  |
| *malayana* sp. nov. | FMNH 268763 | Krabi, Thailand | **8.2683** | **98.9205** |  |
| *malayana* sp. nov. | FMNH 268764 | Krabi, Thailand | **8.2683** | **98.9205** |  |
| *malayana* sp. nov. | FMNH 268768 | Krabi, Thailand | **7.8873** | **99.2901** |  |
| *malayana* sp. nov. | FMNH 268766 | Krabi, Thailand | **9.4792** | **98.9400** |  |
| *malayana* sp. nov. | FMNH 268767 | Krabi, Thailand | ***9.4792*** | ***98.9400*** |  |
| *malayana* sp. nov. | FMNH 268384 | Surat Thani, Thailand | **10.1159** | **98.8372** |  |
| *malayana* sp. nov. | FMNH 268387 | Surat Thani, Thailand | **9.5230** | **98.8278** |  |
| *malayana* sp. nov. | FMNH 268388 | Surat Thani, Thailand | **9.5230** | **98.8278** |  |
| *malayana* sp. nov. | FMNH 268392 | Surat Thani, Thailand | **9.5230** | **98.8278** |  |
| *malayana* sp. nov. | FMNH 268769 | Surat Thani, Thailand | **8.9716** | **98.6393** |  |
| *malayana* sp. nov. | FMNH 268770 | Surat Thani, Thailand | **8.9716** | **98.6393** |  |
| *malayana* sp. nov. | FMNH 268771 | Surat Thani, Thailand | **8.9716** | **98.6393** |  |
| *malayana* sp. nov. | FMNH 268772 | Surat Thani, Thailand | **8.9716** | **98.6393** |  |
| *montosa* sp. nov. | AMS R184961 | Lam Dong, Vietnam | 12.182611 | 108.680028 |  |
| *montosa* sp. nov. | BMNH 1921.4.1.224 | Lam Dong, Vietnam | **11.8274** | **108.6903** |  |
| *montosa* sp. nov. | BMNH 1921.4.1.225 | Lam Dong, Vietnam | **11.8274** | **108.6903** |  |
| *montosa* sp. nov. | FMNH 253792 | Gia-Lai, Vietnam | 14.3333 | 108.6000 |  |
| *montosa* sp. nov. | FMNH 253793 | Gia-Lai, Vietnam | 14.3333 | 108.6000 |  |
| *montosa* sp. nov. | FMNH 253794 | Gia-Lai, Vietnam | 14.3333 | 108.6000 |  |
| *montosa* sp. nov. | FMNH 253795 | Gia-Lai, Vietnam | 14.3333 | 108.6000 |  |
| *montosa* sp. nov. | FMNH 253796 | Gia-Lai, Vietnam | 14.3333 | 108.6000 |  |
| *montosa* sp. nov. | FMNH 254752 | Bolikhamxay, Laos | **18.1948** | **104.9681** |  |
| *montosa* sp. nov. | FMNH 255409 | Khammouan, Laos | 17.5667 | 105.8333 |  |
| *montosa* sp. nov. | FMNH 255410 | Khammouan, Laos | 17.5667 | 105.8333 |  |
| *montosa* sp. nov. | FMNH 255412 | Khammouan, Laos | 17.5667 | 105.8333 |  |
| *montosa* sp. nov. | FMNH 255413 | Khammouan, Laos | 17.5667 | 105.8333 |  |
| *montosa* sp. nov. | FMNH 255414 | Khammouan, Laos | 17.5667 | 105.8333 |  |
| *montosa* sp. nov. | FMNH 255415 | Khammouan, Laos | 17.5000 | 105.8500 |  |
| *montosa* sp. nov. | FMNH 255416 | Khammouan, Laos | 17.3833 | 105.7500 |  |
| *montosa* sp. nov. | FMNH 255417 | Khammouan, Laos | 17.3833 | 105.7500 |  |
| *montosa* sp. nov. | FMNH 255418 | Khammouan, Laos | 17.3333 | 105.6833 |  |
| *montosa* sp. nov. | FMNH 255419 | Khammouan, Laos | 17.2833 | 105.6833 |  |
| *montosa* sp. nov. | FMNH 255420 | Khammouan, Laos | 17.8500 | 104.8667 |  |
| *montosa* sp. nov. | FMNH 255421 | Khammouan, Laos | 17.8500 | 104.8667 |  |
| *montosa* sp. nov. | FMNH 255422 | Khammouan, Laos | 17.8500 | 104.8667 |  |
| *montosa* sp. nov. | FMNH 255423 | Khammouan, Laos | 17.8500 | 104.8667 |  |
| *montosa* sp. nov. | FMNH 255424 | Khammouan, Laos | 17.8500 | 104.8667 |  |
| *montosa* sp. nov. | FMNH 255425 | Khammouan, Laos | 17.8500 | 104.8667 |  |
| *montosa* sp. nov. | FMNH 255426 | Khammouan, Laos | 17.8500 | 104.8667 |  |
| *montosa* sp. nov. | FMNH 255427 | Khammouan, Laos | 17.8833 | 104.9167 |  |
| *montosa* sp. nov. | FMNH 255428 | Khammouan, Laos | 17.5500 | 104.8667 |  |
| *montosa* sp. nov. | FMNH 258116 | Champasak, Laos | 15.0769 | 106.1375 |  |
| *montosa* sp. nov. | FMNH 258117 | Champasak, Laos | 15.0467 | 106.1792 |  |
| *montosa* sp. nov. | FMNH 258118 | Champasak, Laos | 15.0467 | 106.1792 |  |
| *montosa* sp. nov. | FMNH 258119 | Champasak, Laos | 15.0467 | 106.1792 |  |
| *montosa* sp. nov. | FMNH 258120 | Champasak, Laos | 15.0467 | 106.1792 |  |
| *montosa* sp. nov. | FMNH 258121 | Champasak, Laos | 15.0467 | 106.1792 |  |
| *montosa* sp. nov. | FMNH 258141 | Savannakhet, Laos | 16.1358 | 105.9178 |  |
| *montosa* sp. nov. | FMNH 258142 | Savannakhet, Laos | 16.1358 | 105.9178 |  |
| *montosa* sp. nov. | FMNH 258143 | Savannakhet, Laos | 16.1358 | 105.9178 |  |
| *montosa* sp. nov. | FMNH 258198 | Champasak, Laos | 15.0467 | 106.1792 |  |
| *montosa* sp. nov. | FMNH 258200 | Champasak, Laos | 15.0467 | 106.1792 |  |
| *montosa* sp. nov. | FMNH 258201 | Champasak, Laos | 15.0467 | 106.1792 |  |
| *montosa* sp. nov. | FMNH 258202 | Champasak, Laos | 15.0467 | 106.1792 |  |
| *montosa* sp. nov. | FMNH 261971 | Mondolkiri, Cambodia | 12.5378 | 107.5333 |  |
| *montosa* sp. nov. | FMNH 261972 | Mondolkiri, Cambodia | 12.5378 | 107.5333 |  |
| *montosa* sp. nov. | FMNH 261973 | Mondolkiri, Cambodia | 12.5378 | 107.5333 |  |
| *montosa* sp. nov. | FMNH 261975 | Mondolkiri, Cambodia | 12.5378 | 107.5333 |  |
| *montosa* sp. nov. | FMNH 261978 | Mondolkiri, Cambodia | 12.4969 | 107.4925 |  |
| *montosa* sp. nov. | FMNH 262843 | Ratanakiri, Cambodia | 14.1879 | 107.2934 |  |
| *montosa* sp. nov. | FMNH 262844 | Ratanakiri, Cambodia | 14.1879 | 107.2934 |  |
| *montosa* sp. nov. | FMNH 262845 | Ratanakiri, Cambodia | 14.2001 | 107.3083 |  |
| *montosa* sp. nov. | FMNH 262846 | Ratanakiri, Cambodia | 14.2001 | 107.3083 |  |
| *montosa* sp. nov. | FMNH 262848 | Ratanakiri, Cambodia | 14.1879 | 107.2934 |  |
| *montosa* sp. nov. | FMNH 262849 | Ratanakiri, Cambodia | 14.1879 | 107.2934 |  |
| *montosa* sp. nov. | FMNH 262850 | Ratanakiri, Cambodia | 14.1879 | 107.2934 |  |
| *montosa* sp. nov. | FMNH 262851 | Stung Treng, Cambodia | 14.2941 | 106.6124 |  |
| *montosa* sp. nov. | FMNH 262857 | Stung Treng, Cambodia | 14.3079 | 106.5589 |  |
| *montosa* sp. nov. | FMNH 262861 | Mondolkiri, Cambodia | 12.3161 | 107.0997 |  |
| *montosa* sp. nov. | FMNH 262866 | Mondolkiri, Cambodia | 12.2794 | 106.9434 |  |
| *montosa* sp. nov. | FMNH 262867 | Mondolkiri, Cambodia | 12.2794 | 106.9434 |  |
| *montosa* sp. nov. | FMNH 262868 | Mondolkiri, Cambodia | 12.2794 | 106.9434 |  |
| *montosa* sp. nov. | FMNH 262869 | Mondolkiri, Cambodia | 12.2794 | 106.9434 |  |
| *montosa* sp. nov. | FMNH 271365 | Khammouan, Laos | 17.6253 | 105.7173 |  |
| *montosa* sp. nov. | FMNH 271413 | Khammouan, Laos | 17.6253 | 105.7173 |  |
| *montosa* sp. nov. | FMNH 271415 | Khammouan, Laos | 17.6253 | 105.7173 |  |
| *montosa* sp. nov. | FMNH 271417 | Khammouan, Laos | 17.6253 | 105.7173 |  |
| *montosa* sp. nov. | FMNH 271418 | Khammouan, Laos | 17.6253 | 105.7173 |  |
| *montosa* sp. nov. | FMNH 271419 | Khammouan, Laos | 17.6253 | 105.7173 |  |
| *montosa* sp. nov. | NCSM 76382 | Savannakhet, Laos | 17.0443 | 106.1220 |  |
| *montosa* sp. nov. | NCSM 76384 | Savannakhet, Laos | 17.0445 | 106.1262 |  |
| *montosa* sp. nov. | NCSM 76386 | Savannakhet, Laos | 17.0430 | 106.1250 |  |
| *montosa* sp. nov. | NCSM 76387 | Savannakhet, Laos | 16.9685 | 105.8102 |  |
| *montosa* sp. nov. | NCSM 76388 | Savannakhet, Laos | 16.9587 | 106.0683 |  |
| *montosa* sp. nov. | NCSM 76389 | Savannakhet, Laos | 16.9421 | 106.0585 |  |
| *montosa* sp. nov. | NCSM 76390 | Savannakhet, Laos | 16.9421 | 106.0585 |  |
| *montosa* sp. nov. | NCSM 76391 | Savannakhet, Laos | 16.9718 | 106.1666 |  |
| *montosa* sp. nov. | NCSM 76392 | Savannakhet, Laos | 16.9718 | 106.1666 |  |
| *montosa* sp. nov. | NCSM 76393 | Savannakhet, Laos | 16.9718 | 106.1666 |  |
| *montosa* sp. nov. | NCSM 76394 | Savannakhet, Laos | 16.9765 | 106.1721 |  |
| *montosa* sp. nov. | NCSM 76395 | Savannakhet, Laos | 17.0445 | 106.1262 |  |
| *montosa* sp. nov. | NCSM 76396 | Savannakhet, Laos | 17.0445 | 106.1262 |  |
| *montosa* sp. nov. | NCSM 76398 | Savannakhet, Laos | 17.0118 | 106.2201 |  |
| *montosa* sp. nov. | NCSM 76399 | Savannakhet, Laos | 17.0214 | 106.2904 |  |
| *montosa* sp. nov. | NCSM 76400 | Savannakhet, Laos | 17.0214 | 106.2904 |  |
| *montosa* sp. nov. | NCSM 76403 | Savannakhet, Laos | 17.0216 | 106.2890 |  |
| *montosa* sp. nov. | NCSM 76404 | Savannakhet, Laos | 17.0205 | 106.2921 |  |
| *montosa* sp. nov. | NCSM 76405 | Savannakhet, Laos | 17.0205 | 106.2921 |  |
| *montosa* sp. nov. | NCSM 76406 | Savannakhet, Laos | 17.0214 | 106.2904 |  |
| *montosa* sp. nov. | NCSM 76408 | Savannakhet, Laos | 17.0412 | 106.1289 |  |
| *montosa* sp. nov. | NCSM 77403 | Lam Dong, Vietnam | 12.1622 | 108.6650 |  |
| *mortenseni* | BMNH 1915.8.14.14 | Trat, Thailand | 12.0589 | 102.3332 |  |
| *mortenseni* | BMNH 1919.3.28.1 | Trat, Thailand | **12.0589** | **102.3332** |  |
| *mortenseni* | BMNH 1919.3.28.2 | Trat, Thailand | **12.0589** | **102.3332** |  |
| *mortenseni* | BMNH 1947.2.2.51 | Trat, Thailand | **12.0589** | **102.3332** |  |
| *mortenseni* | FMNH 255434 | Bolikhamxay, Laos | 18.4500 | 103.1667 |  |
| *mortenseni* | FMNH 255435 | Bolikhamxay, Laos | 18.4500 | 103.1667 |  |
| *mortenseni* | FMNH 255437 | Champasak, Laos | 14.1167 | 105.4833 |  |
| *mortenseni* | FMNH 255438 | Champasak, Laos | 14.1167 | 105.4833 |  |
| *mortenseni* | FMNH 255439 | Champasak, Laos | 14.1167 | 105.4833 |  |
| *mortenseni* | FMNH 255440 | Champasak, Laos | 14.1333 | 105.3667 |  |
| *mortenseni* | FMNH 255441 | Champasak, Laos | 14.1333 | 105.3667 |  |
| *mortenseni* | FMNH 255442 | Champasak, Laos | 14.1333 | 105.3667 |  |
| *mortenseni* | FMNH 257304 | Siem Reap, Cambodia | 13.4350 | 103.8936 |  |
| *mortenseni* | FMNH 257305 | Siem Reap, Cambodia | 13.4350 | 103.8936 |  |
| *mortenseni* | FMNH 257307 | Siem Reap, Cambodia | 13.5672 | 104.1019 |  |
| *mortenseni* | FMNH 257308 | Siem Reap, Cambodia | 13.5672 | 104.1019 |  |
| *mortenseni* | FMNH 257309 | Siem Reap, Cambodia | 13.5672 | 104.1019 |  |
| *mortenseni* | FMNH 261949 | Kampot, Cambodia | 10.8793 | 104.0250 |  |
| *mortenseni* | FMNH 261950 | Kampot, Cambodia | 10.8793 | 104.0250 |  |
| *mortenseni* | FMNH 261953 | Kampot, Cambodia | 10.6314 | 104.0425 |  |
| *mortenseni* | FMNH 261954 | Kampot, Cambodia | 10.6314 | 104.0425 |  |
| *mortenseni* | FMNH 261956 | Kampot, Cambodia | 10.6578 | 104.0519 |  |
| *mortenseni* | FMNH 261958 | Kampot, Cambodia | 10.6219 | 104.0478 |  |
| *mortenseni* | FMNH 261962 | Kampot, Cambodia | **10.8793** | **104.0544** |  |
| *mortenseni* | FMNH 261963 | Kampong Speu, Cambodia | 11.3667 | 104.1078 |  |
| *mortenseni* | FMNH 261964 | Kampong Speu, Cambodia | 11.3667 | 104.1078 |  |
| *mortenseni* | FMNH 261965 | Kampong Speu, Cambodia | 11.3667 | 104.1078 |  |
| *mortenseni* | FMNH 261968 | Kampong Speu, Cambodia | 11.3667 | 104.1078 |  |
| *mortenseni* | FMNH 261969 | Kampong Speu, Cambodia | 11.3667 | 104.1078 |  |
| *mortenseni* | FMNH 261980 | Kampot, Cambodia | 10.6803 | 104.1003 |  |
| *mortenseni* | FMNH 263299 | Koh Kong, Cambodia | 11.9526 | 103.2908 |  |
| *mortenseni* | FMNH 263318 | Koh Kong, Cambodia | 11.6756 | 103.7252 |  |
| *mortenseni* | FMNH 266293 | Nong Khai, Thailand | 18.2320 | 103.9565 |  |
| *mortenseni* | FMNH 266298 | Nong Khai, Thailand | 18.2320 | 103.9565 |  |
| *mortenseni* | FMNH 266301 | Nong Khai, Thailand | 18.2320 | 103.9565 |  |
| *mortenseni* | FMNH 266302 | Nong Khai, Thailand | 18.2320 | 103.9565 |  |
| *mortenseni* | FMNH 266308 | Ubon Ratchatani, Thailand | 14.4348 | 105.2536 |  |
| *mortenseni* | FMNH 266311 | Ubon Ratchatani, Thailand | 14.4424 | 105.2731 |  |
| *mortenseni* | FMNH 266313 | Ubon Ratchatani, Thailand | 14.4424 | 105.2731 |  |
| *mortenseni* | FMNH 266314 | Ubon Ratchatani, Thailand | 14.4424 | 105.2731 |  |
| *mortenseni* | FMNH 266316 | Ubon Ratchatani, Thailand | 14.4326 | 105.2567 |  |
| *mortenseni* | FMNH 266323 | Sa Kaeo, Thailand | 14.0399 | 102.2657 |  |
| *mortenseni* | FMNH 266326 | Sa Kaeo, Thailand | 14.1277 | 102.2592 |  |
| *mortenseni* | FMNH 266327 | Sa Kaeo, Thailand | 14.1277 | 102.2592 |  |
| *mortenseni* | FMNH 266328 | Sa Kaeo, Thailand | 14.1277 | 102.2592 |  |
| *mortenseni* | FMNH 266330 | Sa Kaeo, Thailand | 13.9953 | 102.2065 |  |
| *mortenseni* | FMNH 266331 | Sa Kaeo, Thailand | 13.9953 | 102.2065 |  |
| *mortenseni* | FMNH 266332 | Sa Kaeo, Thailand | 13.9953 | 102.2065 |  |
| *mortenseni* | FMNH 266334 | Sa Kaeo, Thailand | 14.0933 | 102.3078 |  |
| *mortenseni* | KU 328164 | Nakhon Ratchasima, Thailand | 14.9806 | 102.1000 |  |
| *mortenseni* | KU 328166 | Nakhon Ratchasima, Thailand | 14.9806 | 102.1000 |  |
| *mortenseni* | KU 328167 | Nakhon Ratchasima, Thailand | 14.9806 | 102.1000 |  |
| *mortenseni* | NCSM 77825 | Bolikhamxay, Laos | 18.0333 | 102.6333 |  |
| *mortenseni* | NCSM 77826 | Bolikhamxay, Laos | 18.0333 | 102.6372 |  |
| *mortenseni* | NCSM 78597 | Bolikhamxay, Laos | 18.3950 | 103.0715 |  |
| *mortenseni* | NCSM 78598 | Bolikhamxay, Laos | 18.3950 | 103.0715 |  |
| *mortenseni* | NCSM 79566 | Kampong Speu, Cambodia | 12.0053 | 104.1377 |  |
| *mortenseni* | NCSM 79567 | Kampong Speu, Cambodia | 12.0053 | 104.1377 |  |
| *mortenseni* | NCSM 79568 | Kampong Speu, Cambodia | 12.0016 | 104.1382 |  |
| *mortenseni* | NCSM 79569 | Kampong Speu, Cambodia | 12.0016 | 104.1382 |  |
| *mortenseni* | NCSM 79570 | Kampong Speu, Cambodia | 12.0030 | 104.1382 |  |
| *mortenseni* | NCSM 79619 | Bolikhamxay, Laos | 18.2372 | 103.0425 |  |
| *mortenseni* | NCSM 79620 | Bolikhamxay, Laos | 18.2372 | 103.0425 |  |
| *mortenseni* | NCSM 80249 | Pursat, Cambodia | 12.2073 | 103.0660 |  |
| *mortenseni* | NCSM 80250 | Pursat, Cambodia | 12.2073 | 103.0660 |  |
| *mortenseni* | NCSM 80251 | Pursat, Cambodia | 12.1977 | 103.0528 |  |
| *mortenseni* | NCSM 80910 | Bolikhamxay, Laos | 18.4182 | 104.4012 |  |
| *mortenseni* | NCSM 80911 | Bolikhamxay, Laos | 18.4182 | 104.4012 |  |
| *mortenseni* | NCSM 80912 | Bolikhamxay, Laos | 18.4259 | 104.3926 |  |
| *mortenseni* | NCSM 80913 | Bolikhamxay, Laos | 18.4259 | 104.3926 |  |
| *mortenseni* | NCSM 80914 | Bolikhamxay, Laos | 18.4259 | 104.3926 |  |
| *mortenseni* | NCSM 80915 | Bolikhamxay, Laos | 18.4259 | 104.3926 |  |
| *mortenseni* | NCSM 80916 | Bolikhamxay, Laos | 18.4057 | 104.4215 |  |
| *mortenseni* | ZMUC R072735 | Trat, Thailand | **12.0589** | **102.3332** |  |
| *nigrovittata* | BMNH 1894.5.21.3 | Shan, Myanmar | **20.0000** | **96.5000** |  |
| *nigrovittata* | BMNH 1894.5.21.4 | Shan, Myanmar | **20.0187** | **96.4306** |  |
| *nigrovittata* | BMNH 1929.12.1.2 | Myitkina, Myanmar | 25.2667 | 96.3567 |  |
| *nigrovittata* | BMNH 1947.2.2.99 | Thanintharyi, Myanmar | **12.4389** | **98.6087** |  |
| *nigrovittata* | CAS 210643 | Shan, Myanmar | 20.6983 | 96.5046 |  |
| *nigrovittata* | CAS 230598 | Shan, Myanmar | 21.4069 | 96.3904 |  |
| *nigrovittata* | CAS 232177 | Sagaing, Myanmar | 25.3148 | 95.5285 |  |
| *nigrovittata* | CAS 232201 | Sagaing, Myanmar | 25.3224 | 95.5305 |  |
| *nigrovittata* | CAS 232268 | Sagaing, Myanmar | 25.4750 | 95.6193 |  |
| *nigrovittata* | CAS 232272 | Sagaing, Myanmar | 25.4800 | 95.6196 |  |
| *nigrovittata* | CAS 232277 | Sagaing, Myanmar | 25.4734 | 95.6241 |  |
| *nigrovittata* | CAS 232355 | Kachin, Myanmar | 26.7034 | 96.1949 |  |
| *nigrovittata* | CAS 232371 | Kachin, Myanmar | 26.6865 | 96.2367 |  |
| *nigrovittata* | CAS 232569 | Kachin, Myanmar | 25.1816 | 96.2871 |  |
| *nigrovittata* | CAS 232625 | Kachin, Myanmar | 25.0190 | 96.2266 |  |
| *nigrovittata* | CAS 241148 | Kachin, Myanmar | 25.1341 | 96.4242 |  |
| *nigrovittata* | CAS 241187 | Kachin, Myanmar | 25.2607 | 96.3309 |  |
| *nigrovittata* | CAS 241210 | Kachin, Myanmar | 25.2547 | 96.3457 |  |
| *nigrovittata* | CAS 245392 | Sagaing, Myanmar | 26.1264 | 95.5399 |  |
| *nigrovittata* | FMNH 255429 | Huaphahn, Laos | 20.2333 | 103.2667 |  |
| *nigrovittata* | FMNH 255430 | Huaphahn, Laos | 20.2333 | 103.2667 |  |
| *nigrovittata* | FMNH 255431 | Huaphahn, Laos | 20.2333 | 103.2667 |  |
| *nigrovittata* | FMNH 255432 | Huaphahn, Laos | 20.2333 | 103.2667 |  |
| *nigrovittata* | FMNH 255433 | Huaphahn, Laos | 20.2333 | 103.2667 |  |
| *nigrovittata* | FMNH 258122 | Phongsaly, Laos | 22.0939 | 102.2139 |  |
| *nigrovittata* | FMNH 258125 | Phongsaly, Laos | 22.0939 | 102.2139 |  |
| *nigrovittata* | FMNH 258126 | Phongsaly, Laos | 22.0964 | 102.2189 |  |
| *nigrovittata* | FMNH 258128 | Phongsaly, Laos | 22.0919 | 102.1053 |  |
| *nigrovittata* | FMNH 258204 | Phongsaly, Laos | 22.0939 | 102.2139 |  |
| *nigrovittata* | FMNH 258329 | Phongsaly, Laos | 22.0939 | 102.2139 |  |
| *nigrovittata* | FMNH 258330 | Phongsaly, Laos | 22.1608 | 102.1853 |  |
| *nigrovittata* | FMNH 258534 | Sayaboury, Laos | 18.9022 | 101.5750 |  |
| *nigrovittata* | FMNH 258535 | Sayaboury, Laos | 18.9022 | 101.5750 |  |
| *nigrovittata* | FMNH 261979 | Vientiane, Laos | 18.1325 | 101.4917 |  |
| *nigrovittata* | FMNH 263444 | Prachuap Kirikhan, Thailand | 12.5378 | 99.4614 |  |
| *nigrovittata* | FMNH 263446 | Prachuap Kirikhan, Thailand | 12.5378 | 99.4614 |  |
| *nigrovittata* | FMNH 263447 | Prachuap Kirikhan, Thailand | 12.5378 | 99.4614 |  |
| *nigrovittata* | FMNH 263448 | Prachuap Kirikhan, Thailand | 12.5378 | 99.4614 |  |
| *nigrovittata* | FMNH 266280 | Loei, Thailand | 17.3341 | 101.5090 |  |
| *nigrovittata* | FMNH 266281 | Loei, Thailand | 17.2802 | 101.5186 |  |
| *nigrovittata* | FMNH 266287 | Loei, Thailand | 17.3515 | 101.5037 |  |
| *nigrovittata* | FMNH 266288 | Loei, Thailand | 17.3515 | 101.5037 |  |
| *nigrovittata* | FMNH 268374 | Prachuap Kirikhan, Thailand | **11.5957** | **99.5313** |  |
| *nigrovittata* | FMNH 268380 | Prachuap Kirikhan, Thailand | **11.5957** | **99.5313** |  |
| *nigrovittata* | FMNH 268381 | Prachuap Kirikhan, Thailand | **11.5957** | **99.5313** |  |
| *nigrovittata* | FMNH 268382 | Prachuap Kirikhan, Thailand | **11.5957** | **99.5313** |  |
| *nigrovittata* | FMNH 268760 | Nakhon Si Thammarat, Thailand | **8.4945** | **99.7361** |  |
| *nigrovittata* | FMNH 268761 | Nakhon Si Thammarat, Thailand | **8.4945** | **99.7361** |  |
| *nigrovittata* | FMNH 268762 | Nakhon Si Thammarat, Thailand | **8.4945** | **99.7361** |  |
| *nigrovittata* | FMNH 271354 | Luang Namtha, Laos | 20.8689 | 101.0553 |  |
| *nigrovittata* | FMNH 271358 | Luang Namtha, Laos | 20.8689 | 101.0553 |  |
| *nigrovittata* | FMNH 271359 | Luang Namtha, Laos | 20.8689 | 101.0553 |  |
| *nigrovittata* | FMNH 271371 | Luang Namtha, Laos | 20.8689 | 101.0553 |  |
| *nigrovittata* | FMNH 271374 | Luang Namtha, Laos | 20.8689 | 101.0553 |  |
| *nigrovittata* | FMNH 271375 | Luang Namtha, Laos | 20.8689 | 101.0553 |  |
| *nigrovittata* | FMNH 271409 | Luang Namtha, Laos | 20.8689 | 101.0553 |  |
| *nigrovittata* | FMNH 271410 | Luang Namtha, Laos | 20.8689 | 101.0553 |  |
| *nigrovittata* | KU 331597 | Dien Bien, Vietnam | 22.3866 | 102.2395 |  |
| *nigrovittata* | KU 331598 | Dien Bien, Vietnam | 22.3866 | 102.2395 |  |
| *nigrovittata* | KU 331599 | Dien Bien, Vietnam | 22.3866 | 102.2395 |  |
| *nigrovittata* | KU 331600 | Dien Bien, Vietnam | 22.3866 | 102.2395 |  |
| *nigrovittata* | KU 331602 | Dien Bien, Vietnam | 22.3866 | 102.2395 |  |
| *nigrovittata* | KU 331603 | Dien Bien, Vietnam | 22.3866 | 102.2395 |  |
| *nigrovittata* | KU 331604 | Dien Bien, Vietnam | 22.3866 | 102.2395 |  |
| *nigrovittata* | KU 331605 | Dien Bien, Vietnam | 22.3866 | 102.2395 |  |
| *nigrovittata* | KU 331606 | Dien Bien, Vietnam | 22.3866 | 102.2395 |  |
| *nigrovittata* | KU 331607 | Dien Bien, Vietnam | 22.3866 | 102.2395 |  |
| *nigrovittata* | KU 331608 | Dien Bien, Vietnam | 22.3866 | 102.2395 |  |
| *nigrovittata* | KU 331609 | Dien Bien, Vietnam | 22.3806 | 102.2389 |  |
| *nigrovittata* | KU 331610 | Dien Bien, Vietnam | 22.3806 | 102.2389 |  |
| *nigrovittata* | NCSM 77822 | Huaphahn, Laos | 20.2325 | 103.2108 |  |
| *nigrovittata* | NCSM 77823 | Huaphahn, Laos | 20.2325 | 103.2108 |  |
| *nigrovittata* | NCSM 77824 | Huaphahn, Laos | 20.1879 | 103.2653 |  |
| *nigrovittata* | NCSM 79101 | Xaignabouli, Laos | 18.9550 | 101.7847 |  |
| *nigrovittata* | NCSM 79387 | Luang Phabang, Laos | 19.7491 | 101.9917 |  |
| *nigrovittata* | NCSM 79388 | Luang Phabang, Laos | 19.7491 | 101.9917 |  |
| *nigrovittata* | NCSM 79389 | Luang Phabang, Laos | 19.7491 | 101.9917 |  |
| *nigrovittata* | NCSM 79390 | Luang Phabang, Laos | 19.7491 | 101.9917 |  |
| *nigrovittata* | NCSM 79391 | Luang Phabang, Laos | 19.7491 | 101.9917 |  |
| *nigrovittata* | NCSM 79392 | Luang Phabang, Laos | 19.7491 | 101.9917 |  |
| *nigrovittata* | NCSM 79393 | Luang Phabang, Laos | 19.7491 | 101.9917 |  |
| *nigrovittata* | NCSM 79394 | Luang Phabang, Laos | 19.7491 | 101.9917 |  |
| *nigrovittata* | NCSM 79395 | Luang Phabang, Laos | 19.7491 | 101.9917 |  |
| *nigrovittata* | NCSM 79396 | Luang Phabang, Laos | 19.7491 | 101.9917 |  |
| *nigrovittata* | NCSM 79397 | Luang Phabang, Laos | 19.7491 | 101.9917 |  |
| *nigrovittata* | NCSM 79398 | Xaignabouli, Laos | 19.5276 | 101.8083 |  |
| *nigrovittata* | NCSM 79399 | Xaignabouli, Laos | 19.5276 | 101.8083 |  |
| *nigrovittata* | NCSM 79400 | Xaignabouli, Laos | 19.5276 | 101.8083 |  |
| *nigrovittata* | NCSM 79401 | Xaignabouli, Laos | 19.4378 | 101.8288 |  |
| *nigrovittata* | NCSM 79402 | Luang Phabang, Laos | 19.2454 | 101.8411 |  |
| *nigrovittata* | NCSM 79403 | Luang Phabang, Laos | 19.2454 | 101.8411 |  |
| *nigrovittata* | NCSM 79404 | Luang Phabang, Laos | 19.2451 | 101.8422 |  |
| *nigrovittata* | NCSM 79405 | Luang Phabang, Laos | 19.2451 | 101.8422 |  |
| *nigrovittata* | NCSM 79406 | Xaignabouli, Laos | 19.0304 | 101.7623 |  |
| *nigrovittata* | NCSM 79407 | Xaignabouli, Laos | 19.0304 | 101.7623 |  |
| *nigrovittata* | NCSM 79408 | Xaignabouli, Laos | 18.9527 | 101.7390 |  |
| *nigrovittata* | NCSM 79409 | Xaignabouli, Laos | 18.8259 | 101.8409 |  |
| *nigrovittata* | NCSM 79410 | Xaignabouli, Laos | 18.8259 | 101.8409 |  |
| *nigrovittata* | NCSM 79411 | Xaignabouli, Laos | 18.5139 | 101.6590 |  |
| *nigrovittata* | NCSM 79412 | Xaignabouli, Laos | 18.5139 | 101.6590 |  |
| *nigrovittata* | NCSM 79413 | Vientiane, Laos | 18.4100 | 101.6035 |  |
| *nigrovittata* | NCSM 79920 | Vientiane, Laos | 18.9655 | 102.8486 |  |
| *nigrovittata* | NCSM 79921 | Vientiane, Laos | 18.9655 | 102.8486 |  |
| *nigrovittata* | NCSM 79922 | Vientiane, Laos | 18.9655 | 102.8486 |  |
| *nigrovittata* | NCSM 79923 | Vientiane, Laos | 18.9655 | 102.8486 |  |
| *nigrovittata* | NCSM 79924 | Vientiane, Laos | 19.0005 | 102.8945 |  |
| *nigrovittata* | NCSM 79925 | Vientiane, Laos | 19.0899 | 102.8960 |  |
| *nigrovittata* | NCSM 79926 | Vientiane, Laos | 19.0899 | 102.8960 |  |
| *nigrovittata* | NCSM 79927 | Vientiane, Laos | 19.0899 | 102.8960 |  |
| *nigrovittata* | NCSM 79928 | Vientiane, Laos | 19.0909 | 102.8935 |  |
| *nigrovittata* | NCSM 79929 | Vientiane, Laos | 19.0899 | 102.8960 |  |
| *nigrovittata* | NCSM 79931 | Vientiane, Laos | 19.0899 | 102.8960 |  |
| *nigrovittata* | NCSM 79932 | Vientiane, Laos | 19.1590 | 102.8772 |  |
| *nigrovittata* | NCSM 79933 | Vientiane, Laos | 19.1590 | 102.8772 |  |
| *nigrovittata* | NCSM 79934 | Vientiane, Laos | 19.1575 | 102.8755 |  |
| *nigrovittata* | NCSM 80154 | Vientiane, Laos | 19.1792 | 102.9061 |  |
| *nigrovittata* | NCSM 80645 | Phongsaly, Laos | 21.3329 | 101.8821 |  |
| *nigrovittata* | NCSM 80646 | Phongsaly, Laos | 21.3329 | 101.8821 |  |
| *nigrovittata* | NCSM 80647 | Phongsaly, Laos | 21.3329 | 101.8821 |  |
| *nigrovittata* | NCSM 80649 | Phongsaly, Laos | 21.3329 | 101.8821 |  |
| *nigrovittata* | NCSM 80650 | Phongsaly, Laos | 21.3339 | 101.8828 |  |
| *nigrovittata* | NCSM 80651 | Phongsaly, Laos | 21.3274 | 101.8894 |  |
| *nigrovittata* | NCSM 80652 | Phongsaly, Laos | 21.3382 | 101.8523 |  |
| *nigrovittata* | NCSM 80653 | Phongsaly, Laos | 21.3344 | 101.8414 |  |
| *nigrovittata* | NCSM 80654 | Phongsaly, Laos | 21.3344 | 101.8414 |  |
| *nigrovittata* | NCSM 80655 | Phongsaly, Laos | 21.3922 | 101.8773 |  |
| *nigrovittata* | NCSM 80658 | Phongsaly, Laos | 21.3292 | 101.8773 |  |
| *nigrovittata* | NCSM 80659 | Phongsaly, Laos | 21.3308 | 101.8942 |  |
| *nigrovittata* | NCSM 80660 | Phongsaly, Laos | 21.3313 | 101.8947 |  |
| *nigrovittata* | NCSM 80661 | Phongsaly, Laos | 21.3302 | 101.8935 |  |
| *nigrovittata* | NCSM 80662 | Phongsaly, Laos | 21.3386 | 101.8598 |  |
| *nigrovittata* | NCSM 80663 | Phongsaly, Laos | 21.3386 | 101.8598 |  |
| *nigrovittata* | NCSM 80664 | Phongsaly, Laos | 21.3386 | 101.8598 |  |
| *nigrovittata* | NCSM 80665 | Phongsaly, Laos | 21.3404 | 101.8592 |  |
| *nigrovittata* | NCSM 80666 | Phongsaly, Laos | 21.3404 | 101.8592 |  |
| *nigrovittata* | NCSM 80668 | Phongsaly, Laos | 21.3286 | 101.9009 |  |
| *nigrovittata* | NCSM 80669 | Phongsaly, Laos | 21.3286 | 101.9009 |  |
| *nigrovittata* | NUOL 00005 | Phongsaly, Laos | 21.3320 | 101.8780 |  |
| *nigrovittata* | NUOL 00006 | Phongsaly, Laos | 21.3274 | 101.8894 |  |
| *nigrovittata* | NUOL 00007 | Phongsaly, Laos | 21.3349 | 101.8411 |  |
| *nigrovittata* | NUOL 00008 | Phongsaly, Laos | 21.3308 | 101.8942 |  |
| *nigrovittata* | NUOL 00009 | Phongsaly, Laos | 21.3308 | 101.8946 |  |
| *nigrovittata* | NUOL 00010 | Phongsaly, Laos | 21.3386 | 101.8598 |  |
| *roberti* sp. nov. | CAS 229665 | Tanitharyi, Myanmar | 13.8613 | 98.2883 |  |
| *roberti* sp. nov. | CAS 229796 | Tanitharyi, Myanmar | 13.8453 | 98.4585 |  |
| *roberti* sp. nov. | CAS 243705 | Tanitharyi, Myanmar | 14.5388 | 98.1819 |  |
| *roberti* sp. nov. | CAS 243750 | Tanitharyi, Myanmar | 14.5052 | 98.1943 |  |
| *roberti* sp. nov. | CAS 243792 | Tanitharyi, Myanmar | 14.7323 | 98.2493 |  |
| *roberti* sp. nov. | CAS 243850 | Tanitharyi, Myanmar | 14.7356 | 98.2525 |  |
| *roberti* sp. nov. | CAS 243889 | Tanitharyi, Myanmar | 14.7326 | 98.2514 |  |
| *roberti* sp. nov. | CAS 243897 | Tanitharyi, Myanmar | 14.7329 | 98.2428 |  |
| *roberti* sp. nov. | CAS 243898 | Tanitharyi, Myanmar | 14.7329 | 98.2428 |  |
| *roberti* sp. nov. | CAS 243899 | Tanitharyi, Myanmar | 14.7390 | 98.2404 |  |
| *roberti* sp. nov. | CAS 243902 | Tanitharyi, Myanmar | 14.7379 | 98.2401 |  |
| *roberti* sp. nov. | CAS 243913 | Tanitharyi, Myanmar | 14.7475 | 98.2213 |  |
|  |  |  |  |  |  |
| **Specimens used to calculate species morphology values, but not used in DFA due to lack of samples both sequenced & measured** | | | | | |
| *lacrima* sp. nov. | CAS 235167 | Chin, Myanmar | 21.2715 | 93.7523 |  |
| *lacrima* sp. nov. | CAS 234991 | Chin, Myanmar | 21.7807 | 93.7482 |  |
| *lacrima* sp. nov. | CAS 235087 | Chin, Myanmar | 21.5953 | 93.9363 |  |
| *lacrima* sp. nov. | CAS 235088 | Chin, Myanmar | 21.5953 | 93.9363 |  |
| *lacrima* sp. nov. | CAS 235090 | Chin, Myanmar | 21.5953 | 93.9363 |  |
| *lacrima* sp. nov. | CAS 235091 | Chin, Myanmar | 21.5953 | 93.9363 |  |
| *roberti* sp. nov. | CAS 229678 | Tanitharyi, Myanmar | 13.8613 | 98.2883 |  |
